# Exploring Nitric Oxide (NO)-Releasing Celecoxib Derivatives as Modulators of Radioresponse in Pheochromocytoma Cells

**DOI:** 10.3390/molecules27196587

**Published:** 2022-10-05

**Authors:** Florian Brandt, Martin Ullrich, Verena Seifert, Cathleen Haase-Kohn, Susan Richter, Torsten Kniess, Jens Pietzsch, Markus Laube

**Affiliations:** 1 Department of Radiopharmaceutical and Chemical Biology, Institute of Radiopharmaceutical Cancer Research, Helmholtz-Zentrum Dresden-Rossendorf, Bautzner Landstrasse 400, 01328 Dresden, Germany; 2 Faculty of Chemistry and Food Chemistry, School of Science, Technische Universität Dresden, Mommsenstrasse 4, 01062 Dresden, Germany; 3 Institute of Clinical Chemistry and Laboratory Medicine, University Hospital Carl Gustav Carus at the Technische Universität Dresden, Fetscherstraße 74, 01307 Dresden, Germany

**Keywords:** cancer, endoradionuclide therapy, NO donors, pheochromocytoma, (pseudo)hypoxia, radiation therapy, selective cyclooxygenase-2 inhibitors

## Abstract

COX-2 can be considered as a clinically relevant molecular target for adjuvant, in particular radiosensitizing treatments. In this regard, using selective COX-2 inhibitors, e.g., in combination with radiotherapy or endoradiotherapy, represents an interesting treatment option. Based on our own findings that nitric oxide (NO)-releasing and celecoxib-derived COX-2 inhibitors (COXIBs) showed promising radiosensitizing effects in vitro, we herein present the development of a series of eight novel NO-COXIBs differing in the peripheral substitution pattern and their chemical and in vitro characterization. COX-1 and COX-2 inhibition potency was found to be comparable to the lead NO-COXIBs, and NO-releasing properties were demonstrated to be mainly influenced by the substituent in 4-position of the pyrazole (Cl vs. H). Introduction of the *N*-propionamide at the sulfamoyl residue as a potential prodrug strategy lowered lipophilicity markedly and abolished COX inhibition while NO-releasing properties were not markedly influenced. NO-COXIBs were tested in vitro for a combination with single-dose external X-ray irradiation as well as [^177^Lu]LuCl_3_ treatment in HIF2α-positive mouse pheochromocytoma (MPC-HIF2a) tumor spheroids. When applied directly before X-ray irradiation or ^177^Lu treatment, NO-COXIBs showed radioprotective effects, as did celecoxib, which was used as a control. Radiosensitizing effects were observed when applied shortly after X-ray irradiation. Overall, the NO-COXIBs were found to be more radioprotective compared with celecoxib, which does not warrant further preclinical studies with the NO-COXIBs for the treatment of pheochromocytoma. However, evaluation as radioprotective agents for healthy tissues could be considered for the NO-COXIBs developed here, especially when used directly before irradiation.

## 1. Introduction

Adrenal pheochromocytomas and extra adrenal paragangliomas (PPGLs) are rare catecholamine-producing tumors of chromaffin cell origin that form solid organ metastases mainly in lymph nodes, bones, lungs and the liver [1,2]. Recommended treatment options for metastatic PPGLs include, e.g., surgery, different combinations of chemotherapy, endoradiotherapy using [^131^I]metaiodobenzylguanidine and external radiation therapy, but they are often considered as palliative. Somatostatin type 2 receptor (SSTR2)-targeting endoradiotherapy using particularly [^177^Lu]Lu-DOTA-(Tyr^3^)octreotate ([^177^Lu]Lu-DOTA-TATE) offers potential for treating metastatic PPGLs [3,4]. Because of response rates usually in the range of 30% and 60% [5], this approach is likely to benefit from combination with adjuvant, for example, radiosensitization therapy [6]. In this regard, we previously demonstrated moderate to high cyclooxygenase 2 (COX-2) gene expression and immunoreactivity in about 60% of human PPGLs, indicating that COX-2 can be considered as a clinically relevant molecular target for adjuvant, in particular radiosensitizing treatments using selective COX-2 inhibitors, e.g., in combination with ^177^Lu-DOTA-TATE endoradiotherapy [7].

Cyclooxygenase-2 converts arachidonic acid to prostaglandin H_2_, and by that, produces the key intermediate in the synthesis of prostanoids and thromboxane A_2_ [8]. While COX-2 gene expression is nearly absent in most healthy tissues, it can be induced by pro-inflammatory mediators during acute and chronic inflammatory states as well as in tumor progression and metastasis, exemplified by malignant melanoma [9,10]. In addition, COX-2 is discussed to be responsible for poor response of various types of cancer in radiotherapy [11]. COX-2 exists beside its constitutively expressed isoform COX-1, which shares the same reaction specificity but is mainly involved in homeostatic processes and protection of gastric mucosa. In principle, the analgesic, anti-pyretic, and anti-inflammatory action of COX inhibitors that are classified in nonsteroidal anti-inflammatory drugs (NSAIDs) or selective COX-2 inhibitors (COXIBs) can be attributed to the inhibition of COX-2, while the gastrointestinal toxicity after long-term use especially of NSAIDs results from the concomitant inhibition of COX-1 [8,9,12,13]. Currently, only selected COXIBs such as celecoxib, etoricoxib, or the prodrug parecoxib are on the market due to an increased risk for cardiovascular incidents such as stroke and heart attacks described after long-term treatment with, e.g., rofecoxib or valdecoxib. In this context, ongoing efforts are made to identify COXIBs with optimized pharmacological properties, among them are molecular hybrids, such as dual inhibitors targeting simultaneously the COX and LOX pathway [14,15] or, additionally, releasing gasotransmitters such as H_2_S [16] or, as targeted herein, nitric oxide (NO**^•^**) [17,18,19,20,21,22,23,24,25,26,27].

**Figure 1:**
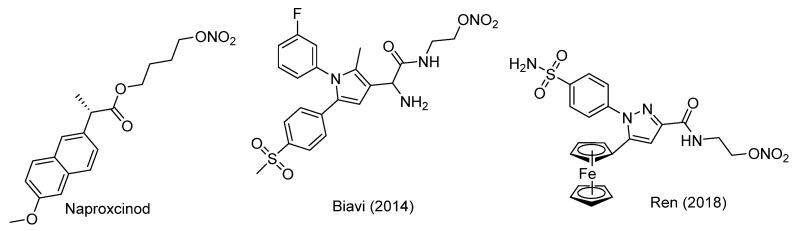
Molecular structures of COX inhibitors with NO^•^ releasing functionalities [39,44,45].

The free radical and endogenous signaling molecule nitric oxide (NO^•^) is produced by three NO synthases: the endothelial (eNOS), the neuronal (nNOS), and the inducible NO synthase (iNOS) [28]. Under physiological conditions, NO^•^ is involved in the stimulation of mucus secretion from gastric cells and the regulation of vascular tone by acting as a vascular smooth muscle relaxant [29,30,31,32]. Some pathologies have been correlated with high levels of NO^•^ produced by iNOS such as septic shock or inflammatory events, whereas others have been found to be connected to reduced levels of NO^•^ such as endothelial dysfunction. For treatment of the latter, clinically approved drugs exist such as glyceryl nitrate or isosorbide dinitrate. With regard to the treatment using COX inhibitors, the concomitant release of NO^•^ is hypothesized to counteract both gastrointestinal and cardiovascular side effects [31,33,34,35]. Starting from naproxcinod [36,37], a variety of novel compounds now known as COX-inhibiting nitric oxide donors (CINODs) or nitric oxide-releasing COXIBs (NO-COXIBs) have been developed [31]. As a common principle for this class, known NSAIDs or COXIBs were functionalized directly or indirectly via a linker structure to an nitric oxide releasing moiety, e.g., nitro ester [38,39,40,41], diazeniumdiolate “NONO” [23], furoxane [40], or sulfohydroxame [41,42] functionalities (Figure 1). NO^•^ shares many actions of cytoprotective prostaglandins, as it stimulates mucus secretion from gastric cells. In this context, NO^•^ has been proven to be protective against NSAID-induced gastropathy [35,43].

**Scheme 1:**
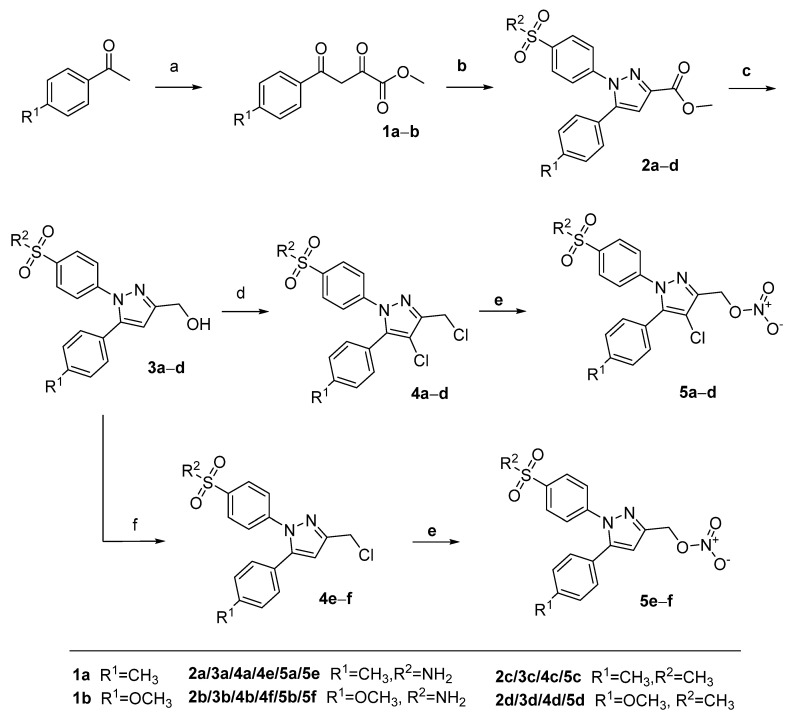
Synthetic route toward NO^•^-releasing COXIBs **5a**–**d**. Reagents and conditions: (**a**) Dimethyloxalate, NaOMe, MeOH, 10 °C > 45 °C, 83% yield for **1b**; (**b**) 4-(R^1^SO_2_)PhNHNH_2_, MeOH, r.t. 71–95% yield; (**c**) LiAlH_4_, THF, r.t., 79–98% yield; (**d**) SOCl_2_, 80 °C, 36–41% yield; (**e**) AgNO_3_, MeCN, 80 °C, 84–86% yield; (**f**) SOCl_2_, DCE, r.t., 46% yield for **4f**.

NO^•^ has been shown to be involved in the cellular response to ionizing radiation leading to the activation of a number of stress proteins such as JNK or MAPK. Upregulation of iNOS is observed after irradiation in a variety of tumor cells and tissues, and it is regarded as one source of endogenous NO^•^ in this context. While low levels of NO^•^ are discussed to be cytoprotective, for higher levels of NO^•^ or reactive nitrogen species, cell damaging effects are discussed [46]. NO^•^ acts as reaction partner for the formation of other highly reactive oxygen/nitrogen species, such as peroxynitrite (ONOO^−^) [47], thereby, e.g., contributing to the fixation of radiation-induced DNA lesions even in highly radioresistant hypoxic tumor areas [48]. From our point of view, the latter property combined with COX inhibition would build an interesting platform for the development of adjuvants for radiation therapy. In this context, we recently developed two novel NO^•^-releasing celecoxib derivatives (**5a** and **5e**, Scheme 1) [49] and evaluated their radiosensitizing properties in comparison to a COX-2 selective celecoxib analog (**3a**, Scheme 1) in vitro [50]. In “COX-2-positive” (A2058) and “COX-2- negative” (Mel-Juso) human melanoma cell lines showing upregulated COX-2 expression under experimental hypoxic conditions and after irradiation, NO-COXIBs **5a** and **5e** showed more potent radiosensitizing efficacy compared to the COXIB **3a** under normoxic and hypoxic conditions at low applied inhibitor concentrations of 10 µM. For example, by administration of **5a**, the required radiation dose for 10% survival could be reduced from 6.6 (DMSO control) to 5.2 Gy for A2058 cells and from 4.2 (DMSO control) to 3.2 Gy for Mel-Juso cells [49].

**Figure 2:**
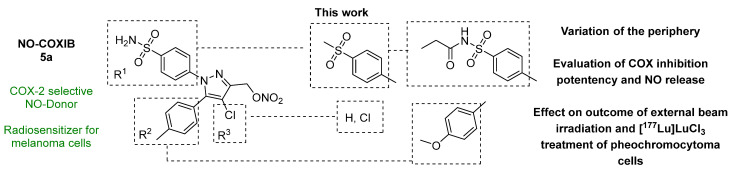
Aim of this work.

To extend the knowledge about the structure-activity relationship on this substance class, we synthesized eight novel derivatives based on the NO-COXIBs **5a** bearing either a 4-tolyl- or 4-methoxyphenyl ring at position 5 of the pyrazole core as well as a methylsulfonyl, an aminosulfonyl or an *N*-propionylsulfonamide group (Figure 2). COX inhibition and NO**^•^** release was measured, and radiosensitizing effects were investigated in vitro. COX-1 and COX-2 inhibition potency was found to be comparable to the lead NO-COXIBs, and NO-releasing properties were found to be mainly influenced by the substituent in the 4-position of the pyrazole. NO-COXIBs showed similar but throughout more radioprotective effects compared to celecoxib.

## 2. Results and Discussion

### 2.1. Synthesis of NO^•^-Releasing COXIBs

Nitroesters **5a**–**d** were synthesized following the synthetic route previously described by us with minor modifications to increase the yield (Scheme 1) [49]. Starting from the respective 4-methyl- or 4-methoxy-substituted acetophenone derivative, the 2,4-dioxobutyrate ester derivatives **1a**–**b** were synthesized by condensation with dimethyl oxalate after deprotonation with sodium methoxide. The pyrazole based heterocycles **2a**–**d** were generated by reaction of **1a**–**b** with the 4-sulfonyl-substituted phenyl hydrazines as commonly applied for the synthesis of celecoxib derivatives. Subsequent reduction of the methyl ester functionality using lithium aluminum hydride in tetrahydrofuran at room temperature furnished the alcohols **3a**–**d**. Conversion to the 3-chloromethyl-4-chloropyrazoles **4a**–**d** was achieved by reaction with neat thionyl chloride. For this reaction, we prolonged the reaction time from 1 h to 8 h compared to our previously published method [49] because for **4a**–**d** after 1 h the mono-chlorinated side product was still observed in considerable amounts. Of note, it was found that the reaction of **3a** and **3b** with thionyl chloride in dichloroethane at 80 °C favored the conversion to the dichloro-substituted products, while stirring at room temperature in dichloroethane exclusively resulted in the formation of 3-chloromethyl-pyrazoles **4e**–**f**. Conversion to the nitroester derivatives **5a**–**d** was achieved by application of silver nitrate in acetonitrile (for discussion of NMR spectra, see Appendix A).

**Scheme 2:**
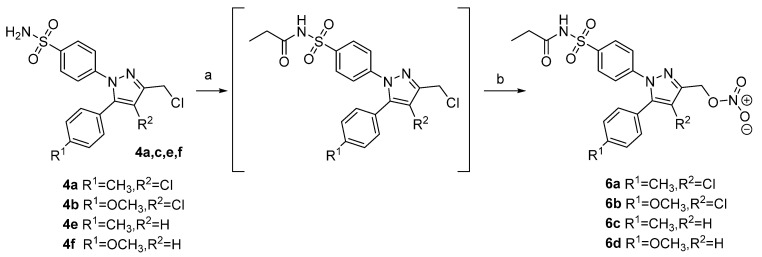
Synthetic route toward propionamides of selected NO^•^-releasing COXIBs **6a**–**d**. Reagents and conditions: (**a**) propionyl chloride, silica sulfate catalyst, CHCl_3_, 60 °C; (**b**) AgNO_3_, MeCN, 80 °C, 36–60% yield over two steps.

With the aim to synthesize more soluble derivatives of the envisaged NO-COXIBs, the sulfonamide derivatives **4a**, **4b**, **4e,** and **4f** were converted to the respective *N*-propionamides followed by conversion of the chloromethyl group into the nitroesters **6a-d** (Scheme 2). For the *N*-propionamide synthesis, sulfuric acid on silica gel was utilized as “dry immobilized” acid as previously described by Massah et al. [51]. This furnished the propionamides as crude products, which were directly reacted with silver nitrate to give the final products **6a**–**d**.

### 2.2. COX Inhibition

COX inhibition (Table 1) was evaluated in vitro using purified ovine COX-1 and recombinant human COX-2 enzyme in a commercially available “COX Fluorescent Inhibitor Screening Assay KIT” (item no. 700100, Cayman Chemical, Ann Arbor, MI, USA). Celecoxib served as a reference and inhibition data concerning **4a**, **4e**, **5a** and **5e** were previously reported by us and is herein discussed for comparison. 

The chloromethyl-substituted celecoxib-derivatives **4a**–**f,** except of **4b**, were found to be selective COX-2 inhibitors with IC_50_(COX-2) ranging between 0.22 and 1.27 µM, while no considerable COX-1 inhibitory potency was found (IC_50_ > 100 µM). Only **4b** showed a COX-1 inhibitory potency (IC_50_ = 0.29 µM) in this subset of compounds and hence a rather non-selective COX inhibition profile. The conversion of the chloromethyl group to the nitroester group of **5a**–**f** was well tolerated and did not markedly influence the inhibitory profile. Compounds **5a**–**f** showed half-maximal inhibition of COX-2 in the range of 0.28-2.12 µM and, in a direct comparison to their respective chloro-analogs, only 2–3 times lower COX-2 inhibitory potency. Only **5b** showed considerable COX-1 inhibition potency while the others did not inhibit COX-1. Both **4b** and **5b** contain an methoxy- and sulfonamide-group at their respective phenyl rings as well as the chloro-substituent at the pyrazole core so that both compounds are electron rich derivatives of this class. In this regard, antioxidative properties that principally disturb the fluorescence-based assay by giving apparent inhibition might be considered in this regard but must be further evaluated. 

The conversion of sulfonamide-residues to their respective *N*-propionamide analogs **6a**–**d** abolished COX inhibitory potency against COX-1 and COX-2. This finding is in line with the general importance of amino- and methyl sulfonyl group for COX-2 selective binding [52,53,54] as well as other literature reports for the synthesis of *N*-propionamide based prodrugs [55,56,57] of cyclooxygenase-inhibitors.

In summary, COX inhibitory potency was found to be consistent with the previously determined inhibition pattern [49], but unfortunately no markedly improved COX-2 inhibition was achieved by the herein performed chemical modifications.

### 2.3. LogD_pH7.4, HPLC_

The lipophilicity of all synthesized compounds was determined as log*D*_7.4, HPLC_ value by an HPLC method originally described by Donovan and Pescatore [58]. Compounds **4a**–**f** and **5a**–**f** were found to be characterized by log*D*_7.4, HPLC_ values in the range of 3.30 to 4.04 while *N*-propionamides **6a**–**d** exerted markedly lower lipophilicity in the range between 1.55 and 2.27. In a direct comparison, Δlog*D*_7.4, HPLC_ between the sulfonamides and their *N*-propionamides were found to be between 1.5 and 1.8, which is comparable to our previous study focusing on (dihydro)pyrrolo[3,2,1-*hi*]indole-based COX inhibitors [55].

### 2.4. NO^•^-Release

**Figure 3:**
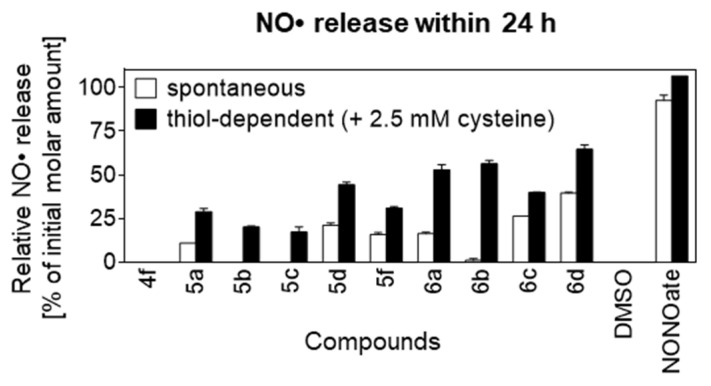
Spontaneous and thiol-dependent NO^•^ release from NO-COXIBs; measured using fluorometric Griess assay (*n* = 2); relative release indicates the percentage of soluble nitrogen species (nitrate + nitrite) detected in the incubation buffer after 24 h compared to the initial molar amount of compounds; NO^•^ release of NONOate (positive control) exceeded the maximum detection limit; data presented as mean ± range.

The nitric oxide releasing properties of the nitroester-derivatives **5a**–**d**, **5f**, and **6a**–**d** were evaluated using a fluorometric Griess assay in the presence and absence of cysteine to determine thiol-dependent and spontaneous release of nitric oxide (Figure 3).

In both blank (DMSO) and negative control (**4f**), release of NO^•^ was not detectable, while the positive control NONOate showed nearly quantitative NO^•^ release in the spontaneous experimental setup. In the thiol-dependent experimental setup, NO^•^ release of NONOate exceeded the assay’s detection limit. For all nitroester derivatives, the thiol dependent NO**^•^**-release was found to be higher than the spontaneous NO^•^ release, which is consistent with a favored thiol-dependent reaction. The chloro-pyrazoles **5b** and **5c** as well as its derived *N*-propionamide **6b** furnished no detectable spontaneous NO^•^ release after 24 h while thiol-dependent release was found to be at 20%, 17% and 56%, respectively. In comparison, the structurally more heterogenous group of **5a**, **5d**, **5f**, and **6a** showed spontaneous NO^•^ release in the range of 10–22%, which was approximately half as high compared to the respective thiol-dependent release ranging from 29–53%. Finally, the *N*-propionamide based pyrazoles **6c** and **6d** sharing a pyrazole with unsubstituted 4-position showed highest spontaneous NO^•^ release of 26% and 40%, respectively, which reached approximately 2/3 compared to the thiol-dependent release of 40% and 65%, respectively.

**Figure 4:**
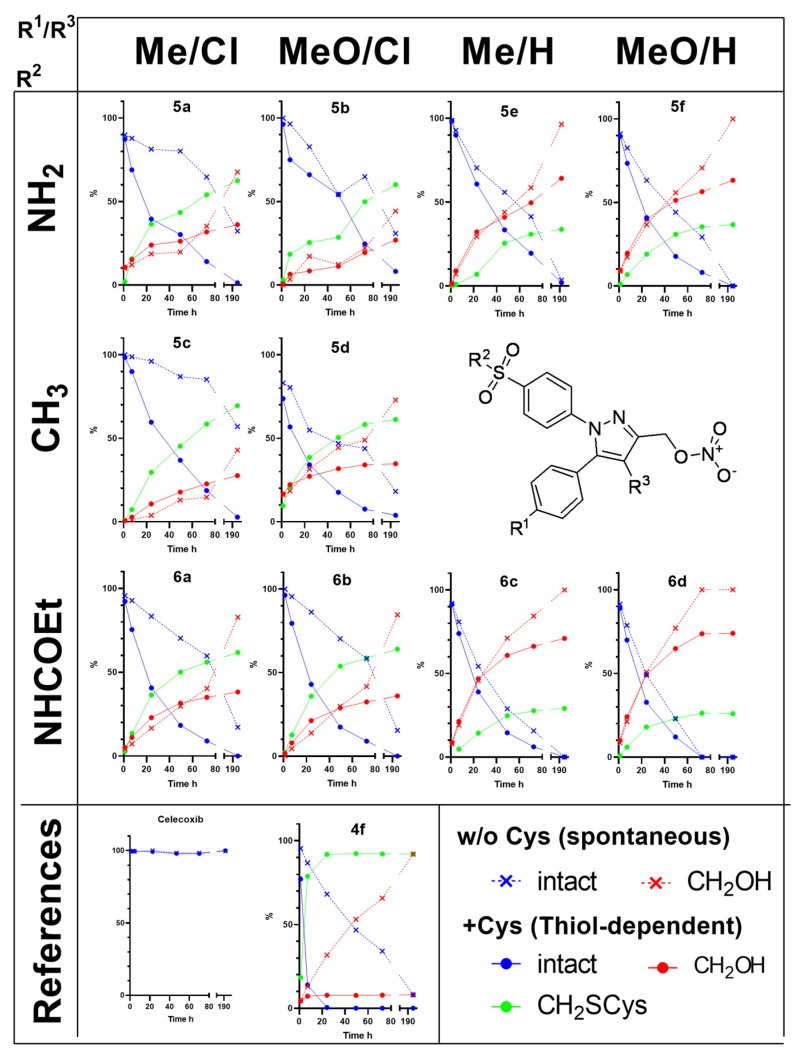
Spontaneous and thiol-dependent conversion of NO-COXIBs followed by HPLC-UV (λ = 254 nm); Y-axis shows relative fraction of intact compound and the respective conversion products, i.e., the hydroxy derivative (CH_2_OH) and the cysteine adduct (CH_2_Cys) detected in the incubation buffer at the indicated time points in the presence or absence of cysteine.

The time-dependent degradation behavior of compounds **4f**, **5a**–**f**, and **6a**–**d** as well as celecoxib was followed by UPLC analysis over a time course of 8 days (Figure 4). For that, compounds were incubated at 37 °C in the presence or absence of cysteine in the assay buffer used for the Griess assay. Samples were withdrawn at distinct time points (1, 5, 23, 47, 70, and 191 h), diluted into a mixture of acetonitrile/water, and analyzed.

Furthermore, HPLC-HRMS was performed after 24 h to obtain more detailed information about the identity of the degradation products. For all investigated compounds, the spontaneous NO^•^ release conditions resulted in the conversion to a more polar product corresponding to the respective alcohol formally resulting from the hydrolysis of the nitroester bond. In comparison, the thiol-dependent release condition resulted in the formation of this and another even more polar product that could be identified to be the *S*-cysteinyl,*S’*-pyrazolylmethyl thioether.

In the presence of cysteine, only **6d** and the chloromethyl-derivative **4f** were quantitatively consumed after 70 h while for the other nitroester derivatives still between 6–25% were found to be intact. In the absence of cysteine, only **6d** was consumed after that time while the amount of intact substance varied to a higher extend, i.e., between 16–85%. For the most stable compound **5c**, still 57% of the compound was found to be intact after 8 days (192 h). Following the time-dependent degradation by UPLC revealed, especially for the substitution pattern in the 4-position of the pyrazole, a pronounced susceptibility to undergo either spontaneous or thiol dependent transformation. While chloro-substituted pyrazoles preferentially reacted to the cysteinyl derivatives and were converted to a lesser extent spontaneously to the hydroxy derivatives, the ratio clearly turned when the 4-position of the pyrazole was unsubstituted. In these cases, spontaneous conversion to the hydroxy derivatives was dominant, and the formation of the cysteinyl-substituted product was diminished. Of note, because the spontaneous conversion can be regarded as the inherent instability of the compounds to undergo hydrolysis of the nitroester group, a stabilizing effect can be deduced from the chloro-substitution at the pyrazole.

### 2.5. Effects on Sensitivity of Tumor Spheroids to External Beam (X-Ray) Irradiation and Lu-177

During acute response to irradiation, inhibition of COX-2 has been reported to suppress DNA repair mechanisms, to inhibit metastatic behavior and to induce apoptosis [13]. Herein, the radiosensitizing properties of NO-COXIBs were tested in vitro on genetically modified HIF2α-expressing mouse pheochromocytoma (MPC-HIF2a) tumor spheroids. In these pseudohypoxic MPC-HIF2α cells, basal expression of the *Ptgs2* gene encoding COX-2 was further stimulated under hypoxic conditions compared to the “empty vector” control cell line MPC-EV (Supplementary Material Figure S67). In MPC-HIF2α spheroids, stabilization of HIF2α in regions of intrinsic hypoxia has been reported to promote a radiation resistant phenotype via yet unknown downstream mechanisms [59]. In various cancer cells, direct upregulation of COX-2 through HIF2α has been reported [60,61].

As a first pilot study, growth responses of MPC-HIF2α spheroids were investigated after two different radiation treatments such as single-dose external beam irradiation (X-ray) and incubation with [^177^Lu]LuCl_3_, respectively. NO-COXIBs were tested at 10 µM for additional effects on radiation treatments against a vehicle-treated control (w/o) and celecoxib (**Ce**). All compounds were applied at a non-toxic concentration that showed no growth attenuation on non-irradiated spheroids (Supplementary Material Table S2).

**Figure 5:**
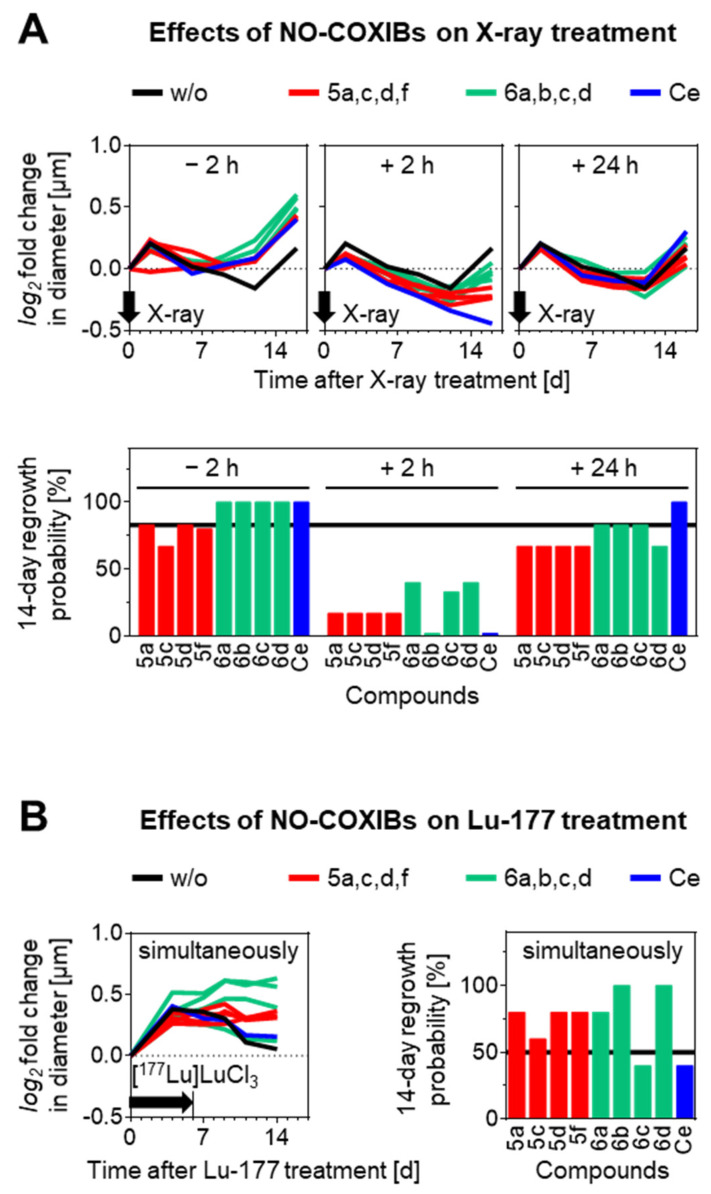
Effects of NO-COXIBs on radiation treatments of MPC-HIF2α tumor spheroids. (**A**) Growth responses to X-ray treatment with a radiation dose of 15 Gy in combination with 10 µM NO-COXIBs added to the medium at indicated time points before or after irradiation. (**B**) Growth responses to [^177^Lu]LuCl_3_ treatment with an initial activity concentration of 0.25 MBq/mL in combination with NO-COXIBs; log_2_ fold changes in diameter indicate the number of diameter doublings compared to treatment start; 14-day regrowth probability indicates the fraction of spheroids able to regrow within 14 days after treatment start; (black arrows) time point or duration of radiation treatment; (w/o) vehicle controls are shown as black lines; (Ce) celecoxib.

In combination with X-ray treatment at a single dose of 15 Gy, NO-COXIBs showed both radioprotective or radiosensitizing effects on tumor spheroids, depending on the time of administration before or after irradiation (Figure 5A and Supplementary Material Figure S68). The log_2_-fold change in spheroid diameter is given as a standardized parameter for spheroid growth, which is positive for growing, near zero for stagnating and negative for shrinking spheroids. Furthermore, regrowth probabilities are given as a parameter to compare how many of the overall studied spheroids in one group recovered within 14 days. Radioprotective effects occurred when compounds were added 2 h before irradiation. In this setup, all compounds accelerated the increase in spheroid size during the regrowth phase compared to controls (Figure 5A, upper panel). Regrowth probabilities of 67–100% under NO-COXIB treatment remained however similar to controls (83%). Radiosensitizing effects occurred when compounds were added 2 h post irradiation. In this setup, all compounds decelerated the increase in spheroid size during the regrowth phase compared to controls. Celecoxib and **6b** most effectively reduced the regrowth probability (0%) followed by **5a**,**c**,**d**,**f** (17%) and **6a**,**c**,**d** (33–40%) compared to controls (83%). Compounds showed no additional effects on spheroid regrowth when added 24 h after irradiation. Overall, the tested NO-COXIBs were found to be more radioprotective than celecoxib in MPC-HIF2α spheroids in response to external beam irradiation, and no significant correlation to COX inhibition potency could be delineated.

Radionuclide therapy is a fast-evolving treatment modality gaining more importance in treating cancer [62,63,64]. For treatment of pheochromocytomas and paragangliomas, in particular, somatostatin type 2 receptor-targeted radionuclide therapy with [^177^Lu]Lu-(Tyr^3^)octreotate is currently investigated in clinical controlled trials (e.g., NCT04029428, NCT03923257, NCT03206060). To assess the additional effects of NO-COXIBs on the outcome of Lu-177 therapy, a simplified in vitro setup was used here, where MPC-HIF2α tumor spheroids were treated with 10 µM of the compounds and simultaneously 0.25 MBq of [^177^Lu]LuCl_3_ for six days. In this setup, most of the NO-COXIBs showed a trend toward radioprotective effects (Figure 5B, Supplementary Material Figure S67). In combination with Lu-177 treatment, compounds **5a**,**c**,**d**,**f**, and **6a**,**c**,**d** prevented spheroids from shrinking in size, whereas **6c** and celecoxib had almost no additional effect on growth response compared to controls. Consistent with this, compounds **6b** and **6d** most effectively increased the regrowth probability of spheroids (100%), followed by **5a**,**c**,**d**,**f** (60–80%), whereas **6c** and celecoxib showed no considerable effects (40%) compared to controls (50%). Overall, the tested NO-COXIBs, except of **6c**, were found to be more radioprotective than celecoxib in MPC-HIF2a spheroids in response to [^177^Lu]LuCl_3_ treatment, and no significant correlation to COX inhibition potency could be delineated.

In principle, these results are in line with other reports demonstrating that application of selective COX-2 inhibitors can have either radioprotective or radiosensitizing effects on irradiated cells depending on a myriad of factors such as cell type, basal and inducible COX-2 levels, modality of irradiation and, the timing of complementary COXIB treatment [13,65,66]. Celecoxib as the main reference compound used in this study was for example shown to enhance radiosensitivity of normoxic and hypoxic glioblastoma cells [67], nasopharyngeal carcinoma cells [68], lung cancer cells (NSCLC) [69], human colorectal carcinoma cells [70] as well as to be radioprotective for human lymphocytes [71] or melanoma (MelJuso) cells [50]. Accordingly, preclinical and clinical studies were performed to unravel the potential application of COXIBs [72] for radioprotection, e.g., to mitigate radiation-induced fibrosis in mice [73] or to reduce the severity of oral mucositis in human [74], as well as for radiosensitization, e.g., during the treatment in FaDu squamous cell carcinoma in mice [75].

Altogether, the NO-COXIBs introduced in this study showed the same tendency as celecoxib, exerting both radioprotective and radiosensitizing effects on MPC-HIF2α spheroids when applied shortly before or after irradiation. However, the effects of NO-COXIBs did not exceed those of celecoxib in terms of radiosensitization. This contrasts the bifunctional effect of **5a** (referred to as compound **5** in our previous report [50]) on clonogenic cell survival under normoxia and hypoxia in melanoma cells showing high (A2058) and low (MelJuso) COX-2 expression. In this monolayer model, in both cell lines, radiosensitizing effects were observed when **5a** was applied at a 10 µM concentration shortly before irradiation and radiosensitivity was enhanced by the additional NO^•^-release compared to celecoxib. Of note, while irradiation upregulated COX-2 expression and no influence of celecoxib or NO-COXIBs was observed on COX-2 protein synthesis in melanoma cells, also in this model, radiosensitization was found to be rather COX-2 independent [50]. Recently, we also demonstrated for COX-2-knockout A2058 melanoma cells (A2058-COX2 KO) that COX-2 independent pathways are most likely involved in the response to X-ray during concomitant treatment with celecoxib and rofecoxib at 1 or 10 µM treatment dose. As an example, radiosensitization was only observed after treatment with 10 µM of celecoxib while all other settings resulted in radioprotection of A2058 and A2058-COX2 KO cells [12]. Principally, a variety of COX-2 independent effects have been described for radiosensitization by celecoxib ranging from cell cycle arrest and prominent autophagy under hypoxic conditions by ER stress loading [67], G2-M phase arrest and apoptosis induction [68], blocking of vasculogenic mimicry through inhibiting off-targets aminopeptidase N (APN) and integrin alpha-V (ITAV) [69] to upregulation of BCCIP (BRCA2 and CDKN1A interacting protein) [70]. Herein, we found celecoxib to exhibit radioprotective effects on MPC-HIF2α spheroids when present during irradiation treatment and to exert radiosensitizing effects only when incubated 2 h after irradiation. Celecoxib did not show any scavenging effects on OH^•^, O_2_^•−^, and OCl^-^ as shown previously by us [76] so that radioprotection exerted when given before irradiation probably does not rely on antioxidant effects. NO-COXIBs showed in comparison to celecoxib more radioprotective effects when applied 2 h before or after irradiation treatment, but no considerable effect when applied after 24 h. In addition, marked radioprotective effects in comparison to celecoxib were observed in combination with [^177^Lu]LuCl_3_ treatment. Generally, the inflammatory state mediated by COX-2 can be active for several days [65,72], whereas DNA damage repair and induction of apoptotic pathways is completed within a few hours after radiation-induced damage [77]. Furthermore inhibition of COX-2 with celecoxib was reported to inhibit the recovery from irradiation-induced injury e.g., by modulating homologous recombination [13,70]. Hence, COX-2 dependent effects are probable factors to modulate the acute radioresponse but as found herein are not the main drivers within MPC-HIF2α cells. In contrast, the time dependency of the treatment was found to prominently influencing the outcome for external beam radiation. Exposure of cells or tissue to ionizing radiation causes damage of DNA, proteins, and lipid membranes directly and indirectly by reactive oxygen as well as reactive nitrogen species, resulting from the radiolysis of water [78]. While radiation-induced cell and protein damage such as DNA double-strand breaks occur in both external beam irradiation and [^177^Lu]LuCl_3_ treatment, less frequent occurrence is expected for the low dose rate of Lu-177 exposure because a lower dose (estimated 2 Gy) is delivered over six days compared to irradiation where 15 Gy was delivered within minutes. Hence, impairment of cellular repair mechanisms by the COX-2-inhibiting properties of the compounds may be less relevant compared to the combination with the high dose rate external beam irradiation. Furthermore, the relatively slow kinetics of NO^•^ release from the NO-COXIBs match with the relatively slow-dose delivery from Lu-177 exposure, indicating that the radioprotective effects of the compounds result from NO^•^ release rather than from COX-2 inhibition.

## 3. Experimental Section

### 3.1. Materials and Methods

All reagents and solvents were commercially available and used without further purification. Column chromatography was performed using silica gel (mesh size 40–63 µm). Dry column vacuum chromatography (DCVC) followed the procedure described by Pedersen and Rosenbohm [79]. Melting points were determined with a melting point apparatus (Galen_TM_ III, Cambridge Instruments, London, UK; Testotherm testo 700, Titisee-Neustadt, Germany; heater: Leica) and are uncorrected. Thin-layer chromatography (TLC) was carried out on Merck silica gel F-254 aluminum plates (Merck KGaA, Darmstadt, Germany), while visualization was performed using UV light (254 nm/366 nm; Benda, UV lamp NU-4). Nuclear magnetic resonance spectra (NMR) were recorded on a 400 MHz spectrometer (Unity INOVA 400 MHz, Varian (now Agilent Technologies, Santa Clara, CA, USA)). The chemical shifts are reported relative to the signal of the residual solvent for ^1^H and ^13^C spectra as internal standard. Copies of NMR spectra are given in Supplementary Material Figures S1–S55.

Analytical HPLC was carried out on the following systems: (System 1) column Luna C18 (Phenomenex Inc., Torrance, CA, USA, 250 × 4.6 mm, 5 µm, 100 Å), Agilent 1200 HPLC (Agilent Technologies, Santa Clara, CA, USA): pump G1311A, auto sampler G1329A, column oven G1316A, degasser G1322A, UV detector G1315D, γ detector Gabi Star (Raytest Isotopenmeßgeräte GmbH, Straubenhardt, Germany), flow rate = 1 mL/min, MeCN/H_2_O + 0.1% TFA 70/30 (*v/v*) gradient 5-95: *t*_0min_ 5/95–*t*_3.0min_ 5/95–*t*_28.0min_ 95/5–*t*_34.0min_ 95/5–*t*_35.0min_ 5/95–*t*_40.0min_ 5/95 or isocratic 70%: *t*_0min_ 70/30–*t*_8.0min_ 70/30–*t*_9.0min_ 90/10–*t*_15.0min_ 90/10–*t*_16.0min_ 70/30–*t*_18.0min_ 70/30; (System 2) column C-18 Jupiter Proteo (Phenomenex Inc., Torrance, CA, USA; 250 × 4.6 mm, 4 µm, 90 Å), Shimadzu prominence modular HPLC system (Shimadzu Corporation, Kyoto, Japan): degasser DGU-20A_5R_, 2x pump LC-20AR, autosampler SIL-20AC_HT_, column oven CTO-20AC with column switching valve, diode array detector SPD-M20A, fluorescence detector RF-20A, and fraction collector FRC-10A, communication bus module CBM-20A, flow rate = 1 mL/min, gradient 0.1% TFA in MeCN/0.1% TFA in water: *t*_0min_ 45/55–*t*_12.5min_ 85/15–*t*_15min_ 95/5–*t*_17.5min_ 95/5–*t*_22.5min_ 45/55–*t*_25min_ 45/55); (System 3) column Aquity UPLC^®^ BEH C18 column (Waters Corporation, Milford, MA, USA, 100 × 2.1 mm, 1.7 µm, 130 Å), UPLC *I*-Class (Waters Corporation, Milford, MA, USA): binary gradient pump BSM, autosampler FTN, column manager CM, and diode array detector PDAeλ coupled to Waters Xevo TQ-S, flow rate 0.4 mL/min, eluent: (A): 0.1% acetic acid in MeCN/MeOH 1/1/ (B): 0.1% acetic acid in H_2_O; bypass mode or gradient: *t*_0min_ 45/55–*t*_0.5min_ 45/55–*t*_5.5min_ 95/5–*t*_7.0min_ 95/5–*t*_8.0min_ 45/55–*t*_8.5min_ 45/55). Preparative HPLC was performed on system 4: column: Varian Dynamax Microsorb (250 × 21.4 mm, 5 µm, 100 Å), Varian Prepstar binary gradient pump system equipped with UV detector (Prostar, Varian (now Agilent Technologies, Santa Clara, CA, USA)), flow rate 10 mL/min, gradient as specified for the purification of the respective compound. The products were monitored at λ = 254 nm unless otherwise specified. The results of logD_7.4HPLC_ value determination are given together with the experimental results, and the protocol is described separately after that section.

Low resolution mass spectrometry (MS) was performed with the mass spectrometer Xevo TQ-S (System 3) by using ESI (electrospray ionization). If indicated, low resolution mass spectrometry was performed using system 5: Advion expression CMS (Advion Inc., Ithaca, NY, USA) by injection of the sample into a constant flow of MeCN/formic acid (99.9/0.1 *v/v*) using electrospray ionization. High resolution mass spectra were obtained on *system 6*: column Kinetex XB-C18 column (Phenomenex Inc., Torrance, CA, USA; 100 mm × 2.1 mm, 2.6 µm, 100 Å), Agilent 1260 Infinity II HPLC (Agilent Technologies, Santa Clara, CA, USA; pump G7111B, autosampler G7129A, column oven G7116N, UV detector G7717C, coupled to UHD Accurate Mass Q-TOF LC MS G6538A (Agilent Technologies, Santa Clara, CA, USA), flow rate 0.5 mL/min, eluent: (A): 0.1% formic acid in MeCN/(B): 0.1% formic acid in H_2_O;bypass or gradient 5-95: *t*_0min_ 5/95–*t*_1.5min_ 5/95–*t*_10.5min_ 95/5–*t*_12.0min_ 95/5–*t*_12.5min_ 5/95–*t*_18min_ 5/95 then 2 min posttime).

### 3.2. Chemistry

Synthesis of starting material **1a** was performed as described in the literature [80], and analytical data were in accordance with published literature findings. The synthesis and characterization of compounds **2a**, **3a**, **4a**, **4e**, **5a**, and **5e** was recently described by us [49]. Selected results obtained within this study are described in the respective section below.

*Preparation of silica sulfate catalyst:* Sulfuric acid (96%, 3 mL) was added to silica gel (9.964 g) in diethyl ether (50 mL) in a round bottom flask. The suspension was mixed at room temperature at a rotary evaporator with a rotation speed of 150 rpm. All volatile components were removed under reduced pressure to yield the catalyst quantitively as a fine white powder.

*Methyl 2,4-dioxo-4-(4-methoxyphenyl)butanoate* (**1b**): Sodium methylate was freshly prepared by dropwise addition of methanol (5 mL) to sodium (0.936 g, 40.7 mmol, 1.22 equiv, stored over benzene) in a cooled flask, evaporation to dryness followed by addition of toluene (25 mL). Under nitrogen atmosphere, to the resulting suspension was added at 5–10 °C dimethyloxalate (4.4 g, 37.3 mmol, 1.11 equiv) followed by addition of 4-methoxyacetophenone (5.0 g, 33.3 mmol, 1.0 equiv) in toluene (5 mL) over a period of 30 min. The solution was allowed to stand overnight. The crude product was isolated by filtration and washing with toluene (2 × 20 mL). Water (20 mL) was added to the solid, and the suspension was acidified with 6 M HCl (15 mL). After extraction with EtOAc, the organic phase was separated, washed with brine (2x), and dried over sodium sulfate. Evaporation under reduced pressure gave the purified product **1b** as a yellow solid (6.99 g, 83.9%): mp: 96 °C; *R*_f_ (petroleum ether/EtOAc 90/10) = 0.20; mp: 81–83 °C; δ _H_ (400 MHz, DMSO-*d*_6_) 3.85 (s, 3 H, OCH_3_), 3.87 (s, 3 H, OCH_3_), 7.07–7.13 (m, 3 H, CH_phenyl_, CH), 8.08 (d, *J* 8.9, 2 H, CH_2,6_), *OH not resolved within the spectra; MS (ESI^+^, M calculated for C_12_H_12_O_5_ = 236.07) *m*/*z* (%): 525.9 (100) [2(*M-H)*+Fe(III)]^+^, 177.3 (93) [*M*-CO_2_Me]^+^; HPLC: *t*_R_ = 23.14 min (95%, system 1-gradient 5–95).

#### 3.2.1. General Synthetic Procedure A

Methyl 2,4-dioxo-4-(aryl)butanoate (4.47 mmol, 1.00 equiv) and 4-(sulfonyl)phenylhydrazine hydrochloride (5.29 mmol, 1.18 equiv) was dissolved in MeOH (52 mL) and heated to reflux for 3.5 h followed by stirring over night at room temperature. The solvent was removed under reduced pressure, and the crude product was stirred with petroleum ether/EtOAc 1/1 (20 mL) at 5 °C, filtered off and washed with a cooled solution of petroleum ether/EtOAc 1/1 (3 × 3 mL). If needed, further purification was performed as given below.

*Methyl 1-(4-sulfamoylphenyl)-5-(4-tolyl)-1H-pyrazole-3-carboxylate* (**2a**): Starting from **1a** (6.77 g, 27.05 mmol, 1.00 equiv) and 4-(aminosulfonyl) phenylhydrazine hydrochloride (6.45 g, 28.86 mmol, 1.07 equiv) following general procedure A and purification by column chromatography (*n*-hexane/EtOAc 60/40), **2a** was obtained as a colorless solid (7.62 g, 71%): mp: 166–168 °C; *R*_f_ (*n*-hexane/EtOAc 60/40) = 0.18; HPLC: *t*_R_ = 21.93 min (98%, system 1-gradient 5–95).

*Methyl 1-(4-sulfamoylphenyl)-5-(4-methoxyphenyl)-1H-pyrazole-3-carboxylate* (**2b**): Starting from **1b** (816 mg, 3.26 mmol, 1.00 equiv) and 4-(aminosulfonyl)phenylhydrazine hydrochloride (730 mg, 3.26 mmol, 1.00 equiv) following general procedure A, **2b** was obtained as a pale yellow solid (1.20 g, 95%): mp: 169–171 °C; *R*_f_ (petroleum ether/EtOAc 60/40) = 0.18; δ _H_ (400 MHz, DMSO-*d*_6_) 3.77 (s, 3 H, OCH_3_), 3.86 (s, 3 H, OCH_3_), 6.96 (d, *J* 8.9, 2 H, CH_phenyl_), 7.08 (s, 1 H, CH_pyrazole_), 7.22 (d, *J* 8.9, 2 H, CH_phenyl_), 7.50–7.54 (m, 4 H, 2xCH_phenyl_/SO_2_NH_2_), 7.87 (d, *J* 8.7, 2 H, CH_phenyl_).; δ _C_ (101 MHz, DMSO-*d*_6_) 51.9, 55.3, 109.7, 114.3, 120.8, 125.8, 126.8, 130.2, 141.5, 143.7, 143.8, 144.6, 159.8, 161.9; MS (ESI^+^, M calculated for C_18_H_17_N_3_O_5_S = 387.09) *m*/*z* (%): 356.1 (100) [*M*-OH]^+^, 388.2 (70) [*M*+H]^+^; HRMS (ESI/QTOF) m/z: [M+H]^+^ Calcd for C_18_H_18_N_3_O_5_S 388.0962, Found 388.0964; HPLC: *t*_R_ = 4.04 min (95%, system 1-70/30).

*Methyl 1-[4-(methylsulfonyl)phenyl]-5-(4-tolyl)-1H-pyrazole-3-carboxylate* (**2c**): Starting from **1a** (2.31 g, 10.3 mmol, 1.00 equiv) and 4-(methylsulfonyl)phenylhydrazine hydrochloride (2.60 g, 11.0 mmol, 1.06 equiv) following general procedure A, and column chromatographic purification (*n*-hexane/EtOAc 70/30), **2c** was obtained as a pale yellow solid (3.20 g, 80%): mp: 178–180 °C; *R*_f_ (petroleum ether/EtOAc 60/40) = 0.21; δ _H_ (400 MHz, DMSO-*d*_6_) 2.31 (s, 3 H, CH_3_), 3.28 (s, 3 H, CH_3_), 3.87 (s, 3 H, OCH_3_), 7.13 (s, 1 H, CH_pyrazole_), 7.19 (d, *J* 8.4, 2 H, CH_tolyl_), 7.22 (d, *J* 8.3, 2 H, CH_tolyl_), 7.59 (d, *J* 8.6, 2 H, CH_SO2-phenyl_), 8.00 (d, *J* 8.6, 2 H, CH_SO2-phenyl_); δ _C_ (101 MHz, DMSO-*d*_6_) 20.8, 43.3, 51.9, 110.1, 125.7, 126.0, 128.2, 128.7, 129.4, 138.9, 140.3, 142.9, 144.0, 144.8, 161.8; MS (ESI^+^, M calculated for C_19_H_18_N_2_O_4_S = 370.10) *m*/*z* (%): 339.1 (100) [*M*-OH]^+^, 371.1 (72) [*M* + H]^+^; HRMS (ESI/QTOF) m/z: [M + H]^+^ Calcd for C_19_H_19_N_2_O_4_S 371.1060, Found 371.1063; HPLC: *t*_R_ = 23.32 min (97%, system 1-gradient 5–95).

*Methyl 1-[4-(methylsulfonyl)phenyl]-5-(4-methoxyphenyl)-1H-pyrazole-3-carboxylate* (**2d**): Starting from **1b** (2.50 g, 10.0 mmol, 1.00 equiv) and 4-(methylsulfonyl)phenylhydrazine hydrochloride (2.79 g, 5.29 mmol, 1.18 equiv) following general procedure A and purification by column chromatography (petroleum ether/EtoAc 70/30), **2d** was obtained as a beige solid (3.47 g, 90%): mp: 158–159 °C; *R*_f_ (petroleum ether/EtOAc 60/40) = 0.19; δ _H_ (400 MHz, CD_3_CN) 3.08 (s, 3 H, CH_3_), 3.80 (s, 3 H, OCH_3_), 3.90 (s, 3 H, OCH_3_), 6.92 (d, *J* 8.8, 2 H, CH_phenyl_), 7.00 (s, 1 H, CH_pyrazole_), 7.21 (d, *J* 8.8, 2 H, CH_phenyl_), 7.54 (d, *J* 8.9, 2 H, CH_SO2-phenyl_), 7.92 (d, *J* 8.7, 2 H, CH_SO2-phenyl_); δ _C_ (101 MHz, CD_3_CN) 44.4, 52.6, 56.0, 110.8, 115.1, 122.2, 126.9, 129.3, 131.4, 141.2, 144.6, 145.6, 146.1, 161.3, 163.3;MS (ESI^+^, M calculated for C_19_H_18_N_2_O_5_S = 386.09) *m*/*z* (%): 355.0 (100) [*M*-OH]^+^, 387.1 (70) [*M* + H]^+^; HRMS (ESI/QTOF) m/z: [M+H]^+^ Calcd for C_19_H_19_N_2_O_5_S 387.1009, Found 387.1013; HPLC: *t*_R_ = 22.21 min (99%, system 1—gradient 5–95).

#### 3.2.2. General Synthetic Procedure B

Methyl 1-[4-(sulfonyl)phenyl]-5-(4-aryl)-1*H*-pyrazole-3-carboxylate (2.51 mmol, 1.0 equiv) was suspended in dry THF (50 mL) followed by portion wise addition of 2.4 M lithium aluminum hydride in THF (amounts as specified below). The mixture was stirred at room temperature for 30 min. After that, the reaction was quenched by addition of water and extracted with EtOAc (4 × 100 mL). The organic phase was combined and dried over sodium sulfate to give the crude product as used for general synthetic procedure D. Further purification was performed to obtain analytical samples as given below.

*4-[3-(Hydroxymethyl)-5-(4-tolyl-1H-pyrazol-1-yl]benzenesulfonamide* (**3a**): Synthesis of **3a** followed the synthesis described recently [49,80] with the modification described herein: Starting from **2a** (0.8 g, 2.15 mmol, 1.00 equiv) following general procedure B using 2.4 M LiAlH_4_ in THF (1.2 mL, 109 mg LiAlH_4_, 2.88 mmol, 1.33 equiv) and purification by column chromatography (petroleum ether/EtOAc 70/30) furnished **3a** as a product (690 mg, 93%, purity determined by HPLC 100%, spectroscopic data were in accordance with the literature).

*4-[3-(Hydroxymethyl)-5-(4-methoxyphenyl)-1H-pyrazol-1-yl]benzenesulfonamide* (**3b**): Starting from **2b** (1.94 g, 5.01 mmol, 1.00 equiv) following general procedure B using 2.4 M LiAlH_4_ in THF (3 mL, 273 mg LiAlH_4_, 7.20 mmol, 1.43 equiv) furnished **3b** as a crude product (1.75 g, 98%, purity determined by HPLC 98%). An analytical sample was obtained by dry column vacuum chromatography (CHCl_3_/MeOH 97.5/2.5): pale yellow solid, mp: 109–112 °C; *R*_f_ (CHCl_3_/MeOH 97.5/2.5) = 0.22; δ _H_ (400 MHz, DMSO-*d*_6_) 3.77 (s, 3 H, OCH_3_), 4.51 (d, *J* 5.7, 2 H, C*H*_2_OH), 5.22 (t, *J* 5.8, 1 H, CH_2_O*H*), 6.55 (s, 1 H, CH_pyrazole_), 6.96 (d, *J* 8.7, 2 H, CH_phenyl_), 7.19 (d, *J* 8.6, 2 H, CH_phenyl_), 7.39–7.45 (m, 4 H, CH_SO2-phenyl_/SO_2_NH_2_), 7.81 (d, *J* 8.5, 2 H, CH_SO2-phenyl_); MS (ESI^+^, M calculated for C_17_H_17_N_3_O_4_S = 359.09) *m*/*z* (%): 342.1 (100) [*M*-OH]^+^, 360.2 (36) [*M*+H]^+^; HRMS (ESI/QTOF) m/z: [M+H]^+^ Calcd for C_17_H_18_N_3_O_4_S 360.1013, Found 360.1019; HPLC: *t*_R_ = 3.15 min (98%, system 1-70/30).

*{1-[4-(Methylsulfonyl)phenyl]-5-(p-tolyl)-1H-pyrazol-3-yl}methanol* (**3c**): Starting from **2c** (1.30 g, 3.51 mmol, 1.00 equiv) following general procedure B using 2.4 M LiAlH_4_ in THF (1.95 mL, 177 mg LiAlH_4_, 4.68 mmol, 1.33 equiv) furnished **3c** as crude product (1.10 g, 87%, purity determined by HPLC 91%). An analytical sample was obtained by dry column vacuum chromatography (CHCl_3_/MeOH 97.5/2.5): pale yellow solid, mp: 127–130 °C); *R*_f_ (CHCl_3_/MeOH 97.5/2.5) = 0.23; δ _H_ (400 MHz, DMSO-*d*_6_) 2.32 (s, 3 H, CH_3_), 3.24 (s, 3 H, CH_3_), 4.52 (d, *J* 5.8, 2 H, C*H*_2_OH), 5.25 (td, *J* 5.8, 0.6, 1 H, CH_2_O*H*), 6.60 (s, 1 H, CH_pyrazole_), 7.16 (d, *J* 8.1, 2 H, CH_phenyl_), 7.22 (d, *J* 8.6, 2 H, CH_phenyl_), 7.48 (d, *J* 8.4, 1 H, CH_SO2-phenyl_), 7.93 (d, *J* 8.4, 1 H, CH_SO2-phenyl_); δ _C_ (101 MHz, DMSO-*d*_6_) 20.8, 43.4, 57.2, 107.8, 124.8, 127.0, 128.0, 128.4, 129.5, 138.3, 138.9, 143.5, 143.7, 155.0; MS (ESI^+^, M calculated for C_18_H_18_N_2_O_3_S = 342,10) *m*/*z* (%): 325.2 (100) [*M*-OH]^+^, 343.2 (37) [*M* + H]^+^; HRMS (ESI/QTOF) m/z: [M+H]^+^ Calcd for C_18_H_19_N_2_O_3_S 343.1111, Found 343.1119; HPLC: *t*_R_ = 3.66 min (91.4%, system 1-isocratic 70%).

*{5-(4-Methoxyphenyl)-1-[4-(methylsulfonyl)phenyl]-1H-pyrazol-3-yl}methanol* (**3d**): Starting from **2d** (1.30 g, 3.36 mmol, 1.00 equiv) following general procedure B using 2.4 M LiAlH_4_ in THF (1.65 mL, 150 mg LiAlH_4_, 3.96 mmol, 1.17 equiv) furnished **3d** as crude product (960 mg, 80%, purity determined by HPLC 97%). An analytical sample was obtained by dry column vacuum chromatography (CHCl_3_/MeOH 100/1): pale yellow solid; mp: 72.8–73.3 °C); *R*_f_ (CHCl_3_/MeOH 100/1) = 0.19; δ _H_ (400 MHz, DMSO-*d*_6_) 3.24 (s, 3 H, CH_3_), 3.77 (s, 3 H, OCH_3_), 4.52 (s, 2 H, C*H*_2_OH), 5.25 (s, 1 H, CH_2_O*H*), 6.57 (s, 1 H, CH_pyrazole_), 6.97 (d, *J* 8.7, 2 H, CH_phenyl_), 7.20 (d, *J* 8.7, 2 H, CH_phenyl_), 7.48 (d, *J* 8.6, 2 H, CH_SO2-phenyl_), 7.93 (d, *J* 8.7, 2 H, CH_SO2-phenyl_); δ _C_ (101 MHz, DMSO-*d*_6_) 43.4, 55.2, 57.2, 107.6, 114.4, 122.1, 124.8, 128.0, 129.9, 138.8, 143.6, 143.6, 155.0, 159.5; MS (ESI^+^, M calculated for C_18_H_18_N_2_O_4_S = 358,10) *m*/*z* (%): 359.1 (100) [*M*+H]^+^; HRMS (ESI/QTOF) m/z: [M+H]^+^ Calcd for C_18_H_19_N_2_O_4_S 359.1060, Found 359.1065; HPLC: *t*_R_ = 3.40 min (97.4%, system 1-70/30).

#### 3.2.3. General Synthetic Procedure C

Under Schlenk conditions, to (5-(4-aryl)-1-(4-(sulfonyl)phenyl)-1*H*-pyrazol-3-yl)methanol (0.84 mmol, 1.00 equiv) was added thionyl chloride (2.20 mL) and the mixture was heated at 80 °C for 8 h. After that, thionyl chloride was distilled off under reduced pressure into a cooling trap where it was afterward deactivated with water. During synthesis and deactivation process, the exhaust gas of the apparatus was passed via an oil filled bubble counter to an alkaline aqueous solution to neutralize acidic gases. If indicated, the crude product was dissolved in EtOAc, adsorbed on silica gel and purified by column chromatography as specified below.

*4-[4-Chloro-3-(chloromethyl)-5-(4-toly)-1H-pyrazol-1-yl]benzenesulfonamide* (**4a**): Synthesis of **4a** was recently described by us [49]. Log*D*_7.4 HPLC_: 3.61 (*t*_R_ = 20.58 ± 0.01 min).

*4-[4-Chloro-3-(chloromethyl)-5-(4-methoxyphenyl)-1H-pyrazol-1-yl]benzenesulfonamide* (**4b**): Starting from **3b** (300 mg, 0.83 mmol, 1.00 equiv) following general procedure C and purification by column chromatography (petroleum ether/EtoAc 75/25), **4b** was obtained as a white solid (129 mg, 38%): mp: 238–240 °C (GalenIII); *R*_f_ (petroleum ether/EtOAc 70/30) = 0.16; δ _H_ (400 MHz, DMSO-*d*_6_) 3.79 (s, 3 H, OCH_3_), 4.85 (s, 2 H, CH_2_), 7.03 (d, *J* 8.8, 2 H, CH_phenyl_), 7.26 (d, *J* 8.8, 2 H, CH_phenyl_), 7.42–7.48 (m, 4 H, CH_SO2-phenyl_/SO_2_NH_2_), 7.82 (d, *J* 8.7, 2 H, CH_SO2-phenyl_); δ _C_ (101 MHz, DMSO-*d*_6_) 36.4, 55.3, 109.8, 114.4, 118.9, 125.0, 126.7, 131.1, 140.1, 141.3, 143.2, 146.3, 160.0; MS (ESI^+^, M calculated for C_17_H_15_Cl_2_N_3_O_3_S, ^35^Cl = 411.02) *m*/*z* (%): 412.1 (100) [*M*+H, ^35^Cl/^35^Cl]^+^, 414.1 (63) [*M*+H, ^35^Cl/^37^Cl]^+^; HRMS (ESI/QTOF/80% MeCN) m/z: [M+H, ^35^Cl]^+^ Calcd for C_17_H_16_Cl_2_N_3_O_3_S 412.0284, Found 412.0284; HPLC: *t*_R_ = 5.87 min (95%, system 1-isocratic 70%); Log*D*_7.4 HPLC_: 3.53 (*t*_R_ = 20.41 ± 0.03 min).

*4-Chloro-3-(chloromethyl)-1-[4-(methylsulfonyl)phenyl]-5-(p-tolyl)-1H-pyrazole* (**4c**): Starting from **3c** (342 mg, 1.00 mmol, 1.00 equiv) following general procedure C, the crude **4c** was obtained as a white solid (160 mg, 41%, purity determined by HPLC 88%). An analytical sample was obtained by dry column vacuum chromatography (petroleum ether/EtOAc 80/20): yellow solid; mp: 176–179 °C); *R*_f_ (petroleum ether/EtOAc 80/20) = 0.22; *R*_f_ (petroleum ether/EtOAc 70/30) = 0.23; δ _H_ (400 MHz, DMSO-*d*_6_) 2.34 (s, 3 H, CH_3_), 3.25 (s, 3 H, CH_3_), 4.86 (s, 2 H, CH_2_), 7.23 (d, *J* 8.2, 2 H, CH_phenyl_), 7.29 (d, *J* 7.9, 2 H, CH_phenyl_), 7.51 (d, *J* 8.7, 2 H, CH_SO2-phenyl_), 7.94 (d, *J* 8.7, 2 H, CH_SO2-phenyl_); δ _C_ (101 MHz, DMSO-*d*_6_) 20.9, 36.3, 43.3, 110.3, 123.9, 125.2, 128.1, 129.6, 129.6, 139.5, 139.9, 140.4, 142.7, 146.7; MS (ESI^+^, M calculated for C_18_H_16_Cl_2_N_2_O_2_S, ^35^Cl = 394.03) *m*/*z* (%): 395.1 (100) [*M* + H, ^35^Cl/^35^Cl]^+^, 397.1 (67) [*M* + H, ^35^Cl/^37^Cl]^+^; HRMS (ESI/QTOF/80%MeCN) m/z: [M + H, ^35^Cl]^+^ Calcd for C_18_H_17_Cl_2_N_2_O_2_S 395.0383, Found 395.0384; HPLC: *t*_R_ = 9.62 min (94%, system 1—gradient 70–90); Log*D*_7.4 HPLC_: 3.90 (*t*_R_ = 22.18 ± 0.09 min).

*4-Chloro-3-(chloromethyl)-5-(4-methoxyphenyl)-1-[4-(methylsulfonyl)phenyl]-1H-pyrazole* (**4d**): Starting from **3d** (300 mg, 0.84 mmol, 1.00 equiv) following general procedure C and purification by column chromatography (petroleum ether/EtOAc 75/25), **4d** was obtained as a white solid (140 mg, 41%): mp: 234–236 °C (GalenIII); *R*_f_ (petroleum ether/EtOAc 70/30) = 0.23; δ _H_ (400 MHz, DMSO-*d*_6_) 3.25 (s, 3 H, CH_3_), 3.79 (s, 3 H, OCH_3_), 4.85 (s, 2 H, CH_2_), 7.04 (d, *J* 8.9, 2 H, CH_phenyl_), 7.28 (d, *J* 8.9, 2 H, CH_phenyl_), 7.51 (d, *J* 8.9, 2 H, CH_SO2-phenyl_), 7.95 (d, *J* 9.0, 2 H, CH_SO2-phenyl_); δ _C_ (101 MHz, DMSO-*d*_6_) 36.4, 43.3, 55.3, 110.1, 114.5, 118.8, 125.1, 128.1, 131.2, 139.8, 140.2, 142.7, 146.6, 160.1; MS (ESI^+^, M calculated for C_18_H_16_Cl_2_N_2_O_3_S, ^35^Cl = 410.03) *m*/*z* (%): 411.1 (100) [*M*+H, ^35^Cl/^35^Cl]^+^, 413.1 (95) [*M*+H, ^35^Cl/^37^Cl]^+^, 413.1 (33) [*M*+H, ^35^Cl/^37^Cl]^+^; HRMS (ESI/QTOF/80%MeCN) m/z: [M+H, ^35^Cl]^+^ Calcd for C_18_H_17_Cl_2_N_2_O_3_S 411.0332, Found 411.0334; HPLC: *t*_R_ = 7.53 min (97%; system 1—isocratic 70%); Log*D*_7.4 HPLC_: 3.84 (*t*_R_ = 21.89 ± 0.16 min).

*4-[3-(Chloromethyl)-5-(p-tolyl)-1H-pyrazol-1-yl]benzenesulfonamide* (**4e**): Synthesis of **4e** was recently described by us [49]. HPLC: *t*_R_ = 9.80 min (100%, System 2); HRMS (ESI/QTOF) m/z: [M+H, ^35^Cl]^+^ Calcd for C_17_H_17_ClN_3_O_2_S 362.0725, Found 362.0727; Log*D*_7.4 HPLC_: 3.14 (*t*_R_ = 18.78 ± 0.03 min).

*4-[3-(Chloromethyl)-5-(4-methoxyphenyl)-1H-pyrazol-1-yl]benzenesulfonamide* (**4f**): Under nitrogen atmosphere, thionyl chloride (2.0 mL, 27.6 mmol, 29.4 equiv) was added to a solution of **3b** (324 mg, 0.94 mmol, 1.00 equiv) in dichloroethane (5 mL) and the reaction mixture was stirred for 13 h at room temperature. The reaction was quenched by addition of saturated potassium hydroxide solution (5 mL), and the aqueous solution was extracted with a mixture of DCM/EtOAc (1/1). The crude product was adsorbed on silica gel and purified by column chromatography (*n*-hexane/EtOAc 65/35) giving **4f** as a pale yellow solid (166 mg, 46%): mp: 169–172 °C (GalenIII); *R*_f_ (*n*-hexane/EtOAc 5/5) = 0.30; δ _H_ (400 MHz, Chloroform-*d*) 3.83 (s, 3H, OCH_3_), 4.68 (s, 2H, CH_2_Cl), 4.89 (s, 2H, SO_2_NH_2_), 6.55 (s, 1H, H_pyrazole_), 6.88 (d, *J* 8.8, 2 H, H_phenyl_), 7.15 (d, *J* 8.8 Hz, 2 H, H_phenyl_), 7.44 (d, *J* 8.7, 2 H, CH_SO2-phenyl_), 7.87 (d, *J* 8.6, 2H, CH_SO2-phenyl_); δ _C_ (101 MHz, Chloroform-*d*) 38.8, 55.5, 108.3, 114.5, 122.0, 125.1, 127.6, 130.3, 140.4, 143.3, 144.9, 150.9, 160.3; MS (ESI^+^, M calculated for C_17_H_16_ClN_3_O_3_S, ^35^Cl = 377.06) *m*/*z* (%): 378.1 (100) [M+H]^+^; 342.0 (70) [M+H-Cl]^+^; HRMS (ESI/QTOF) m/z: [M+H, ^35^Cl]^+^ Calcd for C_17_H_17_ClN_3_O_3_S 378.0674, Found 378.0676; HPLC: *t*_R_ = 2.61 min (97%, system 3-gradient 45-95); Log*D*_7.4 HPLC_: 3.06 (*t*_R_ = 18.24 ± 0.03 min).

#### 3.2.4. General Synthetic Procedure D

Acetonitrile (2.80 mL) was added to 4-chloro-3-(chloromethyl)-5-(4-aryl)-1-[4-(sulfonyl)phenyl]-1*H*-pyrazole (0.25 mmol, 1.00 equiv) and silver nitrate (132 mg, 0.78 mmol, 3.07 mmol). The mixture was heated to 80 °C and stirred for 24 h under reflux.* Afterward, the mixture was filtered to remove precipitated silver chloride and the filter residue was washed with EtOAc (15 mL). Both eluates were combined, and the organic phase was washed with water (3 × 3 mL), dried over sodium sulfate and the crude product adsorbed on silica gel. Purification was performed by column chromatography (petroleum ether/EtOAc 72.5/27.5).

*For higher amounts of starting materials, the reaction should be monitored by HPLC, prolonged if necessary, and kept in the dark for the whole process.

*[4-Chloro-1-(4-sulfamoylphenyl)-5-(p-tolyl)-1H-pyrazol-3-yl]methyl nitrate* (**5a**). Synthesis of **5a** was recently described by us [49]. δ _H_ (400 MHz, Chloroform-*d*) 2.40 (s, 3 H, CH_3_), 4.81 (s, 2 H, SO_2_NH_2_), 5.61 (s, 2 H, CH_2_), 7.15 (d, 2 H, *J* 8.1, CH_tolyl_), 7.23 (d, 2 H, *J* 7.7, CH_tolyl_), 7.39 (d, 2 H, *J* 8.6, CH_SO2-phenyl_), 7.87 (d, 2 H, *J* 8.6, CH_SO2-phenyl_); δ _C_ (101 MHz, Chloroform-*d*) 21.6, 65.8, 112.5, 124.2, 124.8, 127.7, 129.7, 130.0, 140.3, 140.8, 141.0, 142.8, 143.0; HRMS (ESI/QTOF) m/z: [M+H, ^35^Cl]^+^ Calcd for C_17_H_16_ClN_4_O_5_S 423.0525, Found 423.0524; Log*D*_7.4 HPLC_: 3.76 (*t*_R_ = 21.54 ± 0.07 min).

*[4-Chloro-5-(4-methoxyphenyl)-1-(4-sulfamoylphenyl)-1H-pyrazol-3-yl]methyl nitrate* (**5b**): Starting from **4b** (109 mg, 0.26 mmol, 1.00 equiv) and silver nitrate (135 mg, 0.79 mmol, 3.00 mmol) following general procedure D, **5b** was obtained as a white solid (98 mg, 85%): mp: 173–174 °C (GalenIII); *R*_f_ (*n*-hexane/EtOAc 50/50) = 0.3; δ _H_ (400 MHz, DMSO-*d*_6_) 3.79 (s, 3 H, OCH_3_), 5.74 (s, 2 H, CH_2_), 7.03 (d, *J* 8.8, 2 H, CH_phenyl_), 7.26 (d, *J* 8.8, 2 H, CH_phenyl_), 7.42–7.49 (m, 4 H, CH_SO2-phenyl_/SO_2_NH_2_), 7.83 (d, *J* 8.7, 2 H, CH_SO2-phenyl_); δ _C_ (101 MHz, DMSO-*d*_6_) 55.3, 66.2, 110.4, 114.4, 118.7, 125.1, 126.7, 131.2, 140.3, 141.2, 142.2, 143.4, 160.1; MS (ESI^+^, M calculated for C_17_H_15_ClN_4_O_6_S, ^35^Cl = 438.04) *m*/*z* (%): 439.1 (100) [*M*+H, ^35^Cl]^+^, 393.1 (55) [*M*+H-NO_2_, ^35^Cl]^+^; HRMS (ESI/QTOF) m/z: [M+H, ^35^Cl]^+^ Calcd for C_17_H_16_ClN_4_O_6_S 439.0474, Found 439.0472; HPLC: *t*_R_ = 5.49 min (96%, system 1—isocratic 70%); Log*D*_7.4 HPLC_: 3.67 (*t*_R_ = 21.10 ± 0.01 min).

*{4-Chloro-1-[4-(methylsulfonyl)phenyl]-5-(p-tolyl)-1H-pyrazol-3-yl}methyl nitrate* (**5c**): Starting from **4c** (100 mg, 0.25 mmol, 1.00 equiv) following general procedure D, **5c** was obtained as a yellow solid (90 mg, 84%): mp: 113–115 °C (GalenIII); *R*_f_ (*n*-hexane/EtOAc 50/50) = 0.25; δ _H_ (400 MHz, DMSO-*d*_6_) 2.34 (s, 3 H, CH_3_), 3.25 (s, 3 H, CH_3_), 5.75 (s, 2 H, CH_2_), 7.23 (d, *J* 8.2, 2 H, CH_phenyl_), 7.29 (d, *J* 8.0, 2 H, CH_phenyl_), 7.51 (d, *J* 8.7, 2 H, CH_SO2-phenyl_), 7.95 (d, *J* 8.7, 2 H, CH_SO2-phenyl_); δ _C_ (101 MHz, DMSO-*d*_6_) 20.9, 43.2, 66.1, 110.8, 123.7, 125.3, 128.2, 129.6, 129.6, 139.6, 140.1, 140.5, 142.6, 142.6; MS (ESI^+^, M calculated for C_18_H_16_ClN_3_O_5_S, ^35^Cl = 421.05) *m*/*z* (%): 422.1 (100) [*M*+H, ^35^Cl]^+^, 376.1 (65) [*M*+H-NO_2_, ^35^Cl]^+^; HRMS (ESI/QTOF) m/z: [M+H, ^35^Cl]^+^ Calcd for C_18_H_17_ClN_3_O_5_S 422.0572, Found 422.0571; HPLC: *t*_R_ = 8.93 min (96.4%; system 1—isocratic 70%); Log*D*_7.4 HPLC_: 4.04 (*t*_R_ = 22.87 ± 0.11 min).

*{4-Chloro-5-(4-methoxyphenyl)-1-[4-(methylsulfonyl)phenyl]-1H-pyrazol-3-yl}methyl nitrate* (**5d**): Starting from **4d** (115 mg, 0.28 mmol, 1.00 equiv) and silver nitrate (148 mg, 0.87 mmol, 3.12 mmol) following general procedure D, **5d** was obtained as a yellow solid (105 mg, 86%): mp: 141–143 °C (GalenIII); *R*_f_ (*n*-hexane/EtOAc 50/50) = 0.35; δ _H_ (400 MHz, DMSO-*d*_6_) 3.25 (s, 3 H, SO_2_CH_3_), 3.79 (s, 3 H, OCH_3_), 5.74 (s, 2 H, CH_2_), 7.04 (d, *J* 8.9, 2 H, CH_phenyl_), 7.28 (d, *J* 8.9, 2 H, CH_phenyl_), 7.52 (d, *J* 8.9, 2 H, CH_SO2-phenyl_), 7.96 (d, *J* 8.9, 2 H, CH_SO2-phenyl_); δ _C_ (101 MHz, DMSO-*d*_6_) 43.3, 55.3, 66.2, 110.7, 114.5, 118.6, 125.2, 128.1, 131.2, 140.0, 140.4, 142.5, 142.6, 160.1; MS (ESI^+^, M calculated for C_18_H_16_ClN_3_O_6_S, ^35^Cl = 437.04) *m*/*z* (%): 438.1 (100) [*M*+H, ^35^Cl]^+^, 392.1 (60) [*M*+H-NO_2_, ^35^Cl]^+^; HRMS (ESI/QTOF/80%MeCN) m/z: [M+H, ^35^Cl]^+^ Calcd for C_18_H_17_ClN_3_O_6_S 438.0521, Found 438.0523; HPLC: *t*_R_ = 7.28 min (97%, system 1—isocratic 70%); Log*D*_7.4 HPLC_: 4.02 (*t*_R_ = 22.76 ± 0.18 min).

*[1-(4-Sulfamoylphenyl)-5-(p-tolyl)-1H-pyrazol-3-yl]methyl nitrate* (**5e**): Synthesis of **5e** was recently described by us [49]. HRMS (ESI/QTOF) m/z: [M+H]^+^ Calcd for C_17_H_17_N_4_O_5_S 389.0914, Found 389.0914; Log*D*_7.4 HPLC_: 3.40 (*t*_R_ = 19.82 ± 0.02 min).

*[5-(4-Methoxyphenyl)-1-(4-sulfamoylphenyl)-1H-pyrazol-3-yl]methyl nitrate* (**5f**): Starting from **4f** (66 mg, 0.17 mmol, 1.00 equiv) and silver nitrate (92 mg, 0.54 mmol, 3.17 equiv) and following general procedure D for synthesis resulted in the crude product, which was purified by semi-preparative HPLC (system 4, gradient with 0.1% TFA in MeCN/0.1% TFA in water: *t*_0min_ 50/50–*t*_5min_ 50/50–*t*_25min_ 70/30–*t*_27min_ 95/5–*t*_32min_ 95/5–*t*_34min_ 50/50–*t*_41min_ 50/50). After freeze drying, **5f** was obtained as a white solid (39 mg, 55%): mp: 135–141 °C (unstable crystal modification 66–68°, GalenIII); *R*_f_ (hexane/EtOAc 50/50) = 0.21; δ _H_ (400 MHz, Chloroform-*d*) 3.83 (s, 3 H, OCH_3_), 4.89 (s, 2 H, SO_2_NH_2_), 5.56 (s, 2 H, CH_2_), 6.56 (s, 1 H, CH_pyrazole_), 6.88 (d, *J* 8.8, 2 H, CH_phenyl_), 7.14 (d, *J* 8.8, 2 H, CH_phenyl_), 7.44 (d, *J* 8.9, 2 H, CH_SO2-phenyl_), 7.88 (d, *J* 9.0, 2 H, CH_SO2-phenyl_); δ _C_ (101 MHz, Chloroform-*d*) 55.5, 68.3, 108.9, 114.6, 121.7, 125.2, 127.6, 130.3, 140.7, 143.2, 145.1, 146.2, 160.4; MS (ESI^+^, M calculated for C_17_H_16_N_4_O_6_S, ^35^Cl = 404.08; system 5) *m*/*z* (%): 404.1 (100) [*M*+H, ^35^Cl]^+^, 359.0 (80) [*M*+H-NO_2_, ^35^Cl]^+^; HRMS (ESI/QTOF) m/z: [M+H]^+^ Calcd for C_17_H_17_N_4_O_6_S 405.0864, Found 405.0865; HPLC: *t*_R_ = 8.92 min (99%, system 2); Log*D*_7.4 HPLC_: 3.32 (*t*_R_ = 19.46 ± 0.08 min).

### 3.3. Synthesis of N-Propionyl-Substituted Compounds

*{4-Chloro-1-[4-(N-propionylsulfamoyl)phenyl]-5-(p-tolyl)-1H-pyrazol-3-yl}methyl nitrate* (**6a**): Propionyl chloride (40 µL, 0.42 mmol, 2.8 equiv) was added to a suspension of **4a** (59.8 mg, 0.15 mmol, 1.00 equiv) and silica sulfate catalyst (2.7 mg) in chloroform (2.2 mL). The mixture was heated to 70 °C and stirred for 24 h at this temperature with intermediate addition of further propionyl chloride (40 µL, 0.42 mmol, 2.80 equiv, after 5 h). After cooling and removal of the solvent under a stream of nitrogen, acetonitrile (4 mL) and silver nitrate (200 mg, 1.20 mmol, 8.00 equiv) was added and the resulting suspension was stirred at 75 °C for 24 h. After cooling and addition of acetonitrile (3 mL), the suspension was filtered through a syringe filter (0.2 µm). The crude product was purified by semi-preparative HPLC (system 4, gradient with 0.1% TFA in MeCN/0.1% TFA in water: *t*_0min_ 60/40–*t*_5min_ 60/40–*t*_25min_ 80/20–*t*_27min_ 95/5–*t*_32min_ 95/5–*t*_34min_ 60/40–*t*_41min_ 60/40). **6a** was obtained after freeze drying as a pale yellow solid (40.0 mg, 55%): mp: 69–72 °C (GalenIII); *R*_f_ (*n*-hexane/EtOAc 50/50) = 0.25 (tailing); δ _H_ (400 MHz, Chloroform-*d*) 1.08 (t, *J* 7.4, 3 H, C*H*_3_CH_2_), 2.28 (q, *J* 7.4, 2 H, CH_3_C*H*_2_), 2.41 (s, 3 H, CH_3_), 5.60 (s, 2 H, CH_2_ONO_2_), 7.15 (d, *J* 8.3, 2 H, CH_phenyl_), 7.23 (d, *J* 7.9, 2 H, CH_phenyl_), 7.42 (d, *J* 9.0, 2 H, CH_SO2-phenyl_), 8.01 (d, *J* 9.0, 2 H, CH_SO2-phenyl_), 8.15 (s, 1 H, NH); δ _C_ (101 MHz, Chloroform-*d*) 8.3, 21.6, 29.7, 65.7, 112.7, 124.1, 124.5, 129.6*, 130.0, 137.4, 140.4, 140.9, 143.2, 143.7, 171.1, *signal origins from two different carbon species that show coupling in HSQC spectrum to protons with a chemical shift of δ = 7.14 and 8.00 ppm, respectively; MS (ESI^-^, M calculated for C_20_H_19_ClN_4_O_6_S, ^35^Cl = 478.07; system 5) *m*/*z* (%): 477.1 (100) [*M*-H, ^35^Cl]^−^; HRMS (ESI/QTOF) m/z: [M+H, ^35^Cl]^+^ Calcd for C_20_H_20_ClN_4_O_6_S 479.0787, Found 479.0785; HPLC: *t*_R_ = 13.4 min (100%, system 2); Log*D*_7.4 HPLC_: 2.27 (*t*_R_ = 14.51 ± 0.01 min).

*{4-Chloro-5-(4-methoxyphenyl)-1-[4-(N-propionylsulfamoyl)phenyl]-1H-pyrazol-3-yl}methyl nitrate* (**6b**): Propionyl chloride (50 µL, 0.51 mmol, 4.3 equiv) was added to a suspension of **4b** (48.7 mg, 0.12 mmol, 1.00 equiv) and silica sulfate catalyst (8 mg) in chloroform (2.0 mL). The mixture was heated to 60 °C and stirred for 19 h at this temperature. After cooling and removal of the solvent under a stream of nitrogen, acetonitrile (3 mL) and silver nitrate (180 mg, 1.06 mmol, 8.8 equiv) were added, and the resulting suspension was stirred at 77 °C for 26 h. After cooling and addition of acetonitrile (2 mL), the suspension was filtered through a syringe filter (0.2 µm). The crude product was purified by semi-preparative HPLC (system 4, gradient with 0.1% TFA in MeCN/0.1% TFA in water: *t*_0min_ 60/40–*t*_5min_ 60/40–*t*_25min_ 80/20–*t*_27min_ 95/5–*t*_32min_ 95/5–*t*_34min_ 60/40–*t*_41min_ 60/40). **6b** was obtained after freeze drying as a pale yellow solid (35.3 mg, 60%): mp: 193-196 °C (GalenIII); *R*_f_ (*n*-hexane/EtOAc 50/50) = 0.15 (tailing); δ _H_ (400 MHz, Chloroform-*d*) 1.08 (t, *J* 7.4, 3 H, C*H*_3_CH_2_), 2.28 (q, *J* 7.4, 2 H, CH_3_C*H*_2_), 3.86 (s, 3 H, OCH_3_), 5.60 (s, 2 H, CH_2_ONO_2_), 6.94 (d, *J* 8.8, 2 H, CH_phenyl_), 7.19 (d, *J* 8.8, 2 H, CH_phenyl_), 7.43 (d, *J* 9.1, 2 H, CH_SO2-phenyl_), 8.02 (d, *J* 9.1, 2 H, CH_SO2-phenyl_), 8.08 (s, 1 H, NH); δ _C_ (101 MHz, Chloroform-*d*) 8.3, 29.7, 55.5, 65.7, 112.5, 114.8, 119.0, 124.5, 129.7, 131.2, 137.4, 140.7, 143.2, 143.8, 160.8, 171.1; MS (ESI^-^, M calculated for C_20_H_19_ClN_4_O_7_S, ^35^Cl = 494.07; system 5) *m*/*z* (%): 493.0 (100) [*M*-H, ^35^Cl]^−^; HRMS (ESI/QTOF) m/z: [M+H, ^35^Cl]^+^ Calcd for C_20_H_20_ClN_4_O_7_S 495.0736, Found 495.0733; HPLC: *t*_R_ = 25.51 min (96%,system 1-gradient 5-95); Log*D*_7.4 HPLC_: 2.10 (*t*_R_ = 13.67 ± 0.01 min).

*{1-[4-(N-Propionylsulfamoyl)phenyl]-5-(p-tolyl)-1H-pyrazol-3-yl}methyl nitrate* (**6c**): 10 mg silica sulfate catalyst was added to a solution of **4e** (156.7 mg, 0.43 mmol, 1.0 equiv) in chloroform (2 mL) followed by addition of propionyl chloride (100 µL, 1.03 mmol, 2.40 equiv). The mixture was heated to 70 °C and stirred for 21 h at this temperature. After cooling, the solvent was removed under reduced pressure, and the residue redissolved in EtOAc (10 mL) and filtered with a syringe filter (0.2 µm). The organic phase was washed with water (2 mL) and brine (2 mL) followed by drying with Na_2_SO_4_. Removal of the solvent under reduced pressure resulted in the intermediate product *N*-({4-[3-(chloromethyl)-5-(*p*-tolyl)-1*H*-pyrazol-1-yl]phenyl}sulfonyl)propionamide as a brown oil (83.2 mg, 45%): *R*_f_ (petroleum ether/EtOAc 50/50) = 0.35; δ _H_ (400 MHz, Chloroform-*d*) 1.07 (t, *J* 7.4, 3 H, C*H*_3_CH_2_), 2.27 (q, *J* 7.4, 2 H, CH_3_C*H*_2_), 2.38 (s, 3 H, CH_3_), 4.68 (s, 2 H, CH_2_Cl), 6.57 (s, 1 H, CH_pyrazole_), 7.00–7.20 (m, 4 H, CH_phenyl_), 7.46 (d, *J* 8.8 Hz, 2 H, CH_SO2-phenyl_), 7.84–8.09 (m, 2 H, CH_SO2-phenyl_), 8.42 (s, 1 H, NH); δ_C_ (101 MHz, Chloroform-*d*) 8.3, 21.5, 29.7, 38.7, 108.7, 124.8, 126.7, 128.8, 129.5, 129.8, 136.9, 139.5, 144.2, 145.3, 151.1, 171.2; HPLC: *t*_R_ = 11.5 min (100%, system 2). Silver nitrate (150 mg, 0.88 mmol, 6.29 equiv) was added to the intermediate product (60.4 mg, 0.14 mmol, 1.00 equiv) and the mixture was heated to reflux and stirred for 22 h. After cooling, acetonitrile (4 mL) was added followed by filtration through a syringe filter (0.2 µm). Purification by semi-preparative HPLC (system 4, gradient with 0.1% TFA in MeCN/0.1% TFA in water: *t*_0min_ 60/40–*t*_5min_ 60/40–*t*_25min_ 80/20–*t*_27min_ 95/5–*t*_32min_ 95/5–*t*_34min_ 60/40–*t*_41min_ 60/40; t*_R_*= 15.8–17.7 min) and freeze drying provided **6c** as a colorless solid (65 mg, 85%; 36% over two steps): mp: 72–74 °C (GalenIII); *R*_f_ (*n*-hexane/EtOAc 50/50) = 0.22 (tailing); δ _H_ (400 MHz, Chloroform-*d*) 1.08 (t, *J* 7.4, 3 H, C*H*_3_CH_2_), 2.28 (q, *J* 7.4, 2 H, CH_3_C*H*_2_), 2.38 (s, 3 H, CH_3_), 5.56 (s, 2 H, CH_2_ONO_2_), 6.59 (s, 1 H, H_pyrazole_), 7.10 (d, *J* 8.3, 2 H, CH_phenyl_), 7.17 (d, *J* 7.8, 2 H, CH_phenyl_), 7.47 (d, *J* 9.1, 2 H, CH_SO2-phenyl_), 8.02 (d, *J* 9.0, 2 H, CH_SO2-phenyl_), 8.29 (s, 1 H, NH); δ_C_ (101 MHz, Chloroform-*d*) 8.3, 21.5, 29.7, 68.2, 109.3, 124.9, 126.4, 128.8, 129.6, 129.8, 137.2, 139.7, 144.1, 145.4, 146.4, 171.2; MS (ESI^−^, M calculated for C_20_H_20_N_4_O_6_S = 444.46; system 5) *m*/*z* (%): 443.1 (100) [*M*-H]^−^; HRMS (ESI/QTOF) m/z: [M+H]^+^ Calcd for C_20_H_21_N_4_O_6_S 445.1177, Found 445.1174; HPLC: *t*_R_ = 11.4 min (100%, system 2); Log*D*_7.4 HPLC_: 1.82 (*t*_R_ = 12.38 ± 0.02 min).

*{5-(4-Methoxyphenyl)-1-[4-(N-propionylsulfamoyl)phenyl]-1H-pyrazol-3-yl}methyl nitrate* (**6d**): Propionyl chloride (70 µL, 0.72 mmol, 2.7 equiv) was added to a suspension of **4f** (100 mg, 0.26 mmol, 1.00 equiv) and silica sulfate catalyst (15 mg) in chloroform (2.0 mL). The mixture was heated to 60 °C and stirred for 19 h at this temperature. After cooling and removal of the solvent under a stream of nitrogen, acetonitrile (3 mL) and silver nitrate (180 mg, 1.06 mmol, 4.0 equiv) was added, and the resulting suspension was stirred at 77 °C for 26 h. After cooling and addition of acetonitrile (2 mL), the suspension was filtered through a syringe filter (0.2 µm). The crude product was purified by semi-preparative HPLC (system 4, gradient with 0.1% TFA in MeCN/0.1% TFA in water: *t*_0min_ 60/40–*t*_5min_ 60/40–*t*_25min_ 80/20–*t*_27min_ 95/5–*t*_32min_ 95/5–*t*_34min_ 60/40–*t*_41min_ 60/40). **6d** was obtained after freeze drying as a colorless solid (71.6 mg, 59%): mp: 77–80 °C (GalenIII); *R*_f_ (*n*-hexane/EtOAc 50/50) = 0.12 (tailing); δ _H_ (400 MHz, Chloroform-*d*) 1.08 (t, *J* 7.4, 3 H, C*H*_3_CH_2_), 2.28 (q, *J* 7.4, 2 H, CH_3_C*H*_2_), 3.84 (s, 3 H, OCH_3_), 5.55 (s, 2 H, CH_2_ONO_2_), 6.56 (s, 1 H, CH_pyrazole_), 6.88 (d, *J* 8.8, 2 H, CH_phenyl_), 7.14 (d, *J* 8.9, 2 H, CH_phenyl_), 7.48 (d, *J* 9.1, 2 H, CH_SO_2_-phenyl_), 8.03 (d, *J* 9.0, 2 H, CH_SO2-phenyl_), 8.13 (s, 1 H, NH); δ _C_ (101 MHz, Chloroform-*d*) 8.3, 29.7, 55.5, 68.2, 109.0, 114.6, 121.6, 125.0, 129.6, 130.3, 137.2, 144.1, 145.2, 146.4, 160.5, 171.1; MS (ESI^-^, M calculated for C_20_H_20_N_4_O_7_S = 460.11; system 5) *m*/*z* (%): 459.1 (100) [*M*-H]^−^; HRMS (ESI/QTOF) m/z: [M+H]^+^ Calcd for C_20_H_21_N_4_O_7_S 461.1126, Found 461.1126; Log*D*_7.4 HPLC_: 1.55 (*t*_R_ = 11.10 ± 0.02 min).

### 3.4. Determination of COX Inhibition

COX inhibition activity against ovine COX-1 and human COX-2 was determined using the fluorescence-based COX assay “COX Fluorescent Inhibitor Screening Assay Kit” (catalog number 700100; Cayman Chemical, Ann Arbor, MI, USA) according to the manufacturer’s instructions. Compounds **6a**–**d** were screened at a concentration of 100 µM, all others were assayed in a concentration range of 0.1 nM to 100 μM in duplicate.

### 3.5. Determination of Lipophilicity

The logD_7.4HPLC_ value was determined as previously reported by us [81] utilizing an HPLC method originally described by Donovan and Pescatore [58]. Hydrocortisone (*t*_R_ 10.66 min, log*D*_7.4_ 1.46 (mean of 1.55 [82] and log*D*_7.4_ 1.37 [82]), toluene (*t*_R_ 16.45 min, measured log*D*_7.4_ 2.70, Literature log*D*_7.4_ 2.72 [58]), and triphenylene (*t*_R_ 29.69 min, log*D*_7.4_ 5.49 [58]) served as references and the following HPLC system was used: Agilent 1100 HPLC (Agilent Technologies, Santa Clara, CA, USA; binary pump G1312A, autosampler G1313A, column oven G1316A, degasser G1322A, UV detector G1314A, γ detector Gabi Star (Raytest Isotopenmeßgeräte GmbH, Straubenhardt, Germany); column ODP-50 4B (Showa Denko K.K., Tokyo, Japan, Shodex Asahipak 50 × 4.6 mm); eluent: MeOH/phosphate buffer (10 mM, pH 7.4) gradient *t*_0min_ 30/70–*t*_25min_ 95/5–*t*_27min_ 95/5–*t*_28min_ 30/70–*t*_40min_ 30/70, flow rate = 0.6 mL/min.

### 3.6. Fluorometric NO^•^ Release Assay (Griess Assay)

Nitric oxide release of compounds within 24 h at 37 °C was measured using the fluorometric nitric oxide microplate assay kit ab65327 (Abcam, Cambridge, UK). For measurement of spontaneous release, 1.8 nmol of compounds were incubated in 75 µL Dulbecco’s PBS containing 0.05% *v/v* residual DMSO and 2.5 mM DTPA (pH = 7.1) for 24 h at 37 °C. Thiol-dependent release was measured accordingly in presence of 2.5 mM cysteine. Two series of nitrate standards were prepared (0.2−1 nmol) in 75 µL incubation buffer, in presence or absence of cysteine, respectively. According to the manufacturer’s instructions, all nitrate content in samples and standards was enzymatically converted into nitrite through incubation with nitrate reductase for 2 h at room temperature, and the resulting nitrite content (total nitric oxide) was determined through reaction with 2,3-diaminonaphthalene followed by measurement of fluorescence intensity at λ_ex/em_ = 360/450 nm using a microplate reader. Nitrite content in samples was calculated from linear regression of standards and expressed as relative nitric oxide release (%) normalized to the initial molar amount of compound. NONOate served as positive control.

### 3.7. Conversion in NO^•^-Assay Buffer followed by UPLC and HPLC-HRMS

The time-dependent conversion of NO-COXIBs **5a**–**f** and **6a**–**d** as well as reference compounds **4f** and celecoxib in the presence or absence of cysteine in assay buffer was investigated by following the degradation of intact compound and identification of selected degradation products by UPLC. For that, samples were incubated at 37 °C in the assay buffer in the presence or absence of cysteine and at the indicated time point; 5 µL assay buffer of each sample was withdrawn and diluted with 45 µL MeCN/water (50/50 *v/v*). The samples were thoroughly vortexed and then subjected to UPLC analysis. All samples of one time point were analyzed within a total analysis time of approximately 2.5 h. Conversion was monitored at 254 nm using the following UPLC system: column Aquity UPLC^®^ HSS C18 column (Waters Corporation, Milford, MA, USA; 100 × 2.1 mm, 1.8 µm, 130 Å), UPLC *H*-Class (Waters Corporation, Milford, MA, USA): quaternary gradient pump QSM, autosampler FTN, column heater CHA, and diode array detector PDAeλ, γ detector Gabi Star (Raytest Isotopenmeßgeräte GmbH, Straubenhardt, Germany), flow rate 0.4 mL/min, eluent: (A): 0.1% acetic acid in MeCN/MeOH 1/1/(B): 0.1% acetic acid in H_2_O; gradient: *t*_0min_ 45/55–*t*_0.5min_ 45/55–*t*_5.5min_ 95/5–*t*_7.0min_ 95/5–*t*_7.2min_ 45/55–*t*_8.5min_ 45/55). For the identification of degradation products, samples taken after 24 h were additionally subjected to HPLC-HRMS analysis using HPLC system 6. In brief, the NO-COXIBs were converted to the respective hydroxymethyl-substituted compounds and in the presence of cysteine additionally to the cysteine adduct, which could be identified by the respective *Δm/z* shifts. A detailed comparison of calculated and found m/z-signals is given in Supplementary Material Table S1, and copies of HPLC-HRMS chromatograms and selected spectra are given in Supplementary Material Figures S56–S66.

### 3.8. Gene Expression Analysis

mRNA was extracted from MPC-EV and MPC-HIF2α cells cultured as monolayers using the miRNeasy Mini Kit (Qiagen, Holden, Germany). cDNA was prepared using qScript cDNA Synthesis Kit (Quantabio, Beverly MA, USA) following the manufacturer’s instructions. Amplification of cDNA was performed on a CFX Connect Real-Time PCR Detection System (Bio-Rad, Hercules CA, USA) using the PerfeCTa SYBR Green Super Mix Low Rox (Quantabio, Beverly MA, MA, USA) and primers for *Ptgs1* (5′-ATGAGTCGAAGGAGTCTCTCG-3′, 5′-GCACGGATAGTAACAACAGGGA-3′), *Ptgs2* (5′-TGAGCAACTATTCCAAACCAGC-3′, 5′-GCACGTAGTCTTCGATCACTATC-3′), *Actb* (5′-GGCTGTATTCCCCTCCATCG-3′, 5′-CCAGTTGGTAACAATGCCATGT-3′), and *Rpl19* (5′-ATATGGGCATAGGGAAGAGG-3′, 5′-CTGTCTGCCTTCAGCTTGT-3′). Amplicons were generated in 40 cycles (95 °C 5 s, 60 °C 10 s) with 5 min at 95 °C for initial denaturation and characterized by melting curve analysis and on an agarose gel.

### 3.9. Spheroid Regrowth Assay

Genetically modified mouse pheochromocytoma (MPC) cells expressing a codon-optimized *Epas1* gene (encoding HIF2α) were cultured as three-dimensional tumor spheroids as published elsewhere [83]. External X-ray beam radiation treatment and [^177^Lu]LuCl_3_ radionuclide treatment was performed as reported previously [59] with some modifications. In brief, spheroids were irradiated with a 200 kV photon beam using a Maxishot X-ray system equipped with a Y.TU/320-D03 tube (YXLON, Hamburg, Germany), growth of spheroids (*n* = 6) with diameters between 300−400 µm was measured in response to one fixed radiation dose, 15 Gy for X-ray treatment, and 0.25 MBq/mL of [^177^Lu]LuCl_3_ for radionuclide treatment corresponding to a cumulative dose of approximately 2 Gy delivered for 6 days. The applied radiation doses were selected based on the half-maximal spheroid control doses (SCD_50_) as previously determined for MPC HIF2α spheroids by us [59]. Spheroids were considered as regrown at 100 µm increase in diameter compared to treatment start or at a diameter growth rate of 15 µm per day. Results are reported as spheroid regrowth probability (%).

### 3.10. Statistical Analyses

Statistical analyses were performed using Prism 8 (GraphPad, La Jolla, CA, USA). Data were visualized as mean ± SEM.

## 4. Summary and Conclusions

The aim of the present work was to extend the knowledge about NO-COXIBs based on the previously described lead **5a**. COX-inhibition potency of the newly synthesized COXIBs (**5b**–**d**,**f**) differing in the periphery was comparable to their lead **5a**, while the introduction of an *N*-propionamide residue at the sulfamoyl group of **6a**–**d** abolished inhibition potency as expected, as part of a prodrug strategy that lowered lipophilicity considerably. All synthesized compounds were found to release nitric oxide as determined by Griess assay, and differences in the time-dependent release were found to be mainly influenced by the substitution at the pyrazole. The effect of NO-COXIBs on genetically modified HIF2α-positive mouse pheochromocytoma (MPC-HIF2α) tumor spheroids was tested to evaluate the application of the compounds with respect to external beam irradiation or radionuclide therapy. In this artificial model, NO-COXIBs were found to have a radioprotective effect. This raises the question, if radioresponse to treatment would be similar or different in the in vivo situation of this cancer entity. Furthermore, future evaluation as radioprotectant if applied before radiation events on normal tissue should be considered based on the principal COX-2 selective inhibition and nitric oxide-releasing properties as well as the potential to use the respective more hydrophilic prodrugs introduced herein. In this regard, determining the optimal dosage at an optimal time window for treatment is a very important point to consider when planning the use of coxibs and only NO-COXIBs. This aspect has already been highlighted for other potential applications of coxibs and NO-COXIBs as adjuvants, for example in the modulation of inflammatory processes in bone healing [84].

## Figures and Tables

**Table 1:** COX inhibition and log*D*_7.4, HPLC_ data for compounds **4b**–**d** and **5b**–**d**.

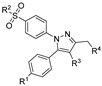	R^1^	R^2^	R^3^	R^4^	IC_50_ (COX-1) [µM]	IC_50_ (COX-2) [µM]	SI	Log*D*_7.4_
4a *	CH_3_	NH_2_	Cl	Cl	>100	0.22	>454	3.61
**4b**	OCH_3_	NH_2_	Cl	Cl	0.297	0.127	2.3	3.53
**4c**	CH_3_	CH_3_	Cl	Cl	>100	0.72	>138	3.90
**4d**	OCH_3_	CH_3_	Cl	Cl	>100	0.480	>208	3.84
4e *	CH_3_	NH_2_	H	Cl	>100	1.27	>78	3.14
**4f**	OCH_3_	NH_2_	H	Cl	>100	0.37	>270	3.06
5a *	CH_3_	NH_2_	Cl	ONO_2_	61.0	0.28	>217	3.76
**5b**	OCH_3_	NH_2_	Cl	ONO_2_	2.573	0.434	5.9	3.67
**5c**	CH_3_	CH_3_	Cl	ONO_2_	>100	2.120	>47	4.04
**5d**	OCH_3_	CH_3_	Cl	ONO_2_	>100	1.082	>92	4.02
5e *	CH_3_	NH_2_	H	ONO_2_	>100	0.89	>112	3.40
**5f**	OCH_3_	NH_2_	H	ONO_2_	>100	1.18	>84	3.32
6a **	CH_3_	NHCOEt	Cl	ONO_2_	>100	>100	-	2.27
6b **	OCH_3_	NHCOEt	Cl	ONO_2_	~100	>100	-	2.10
6c **	CH_3_	NHCOEt	H	ONO_2_	>100	>100	-	1.82
6d **	OCH_3_	NHCOEt	H	ONO_2_	>100	>100	-	1.55

* COX inhibition previously reported [49]; ** compounds **6a**–**d** were screened at a concentration of 100 μM.

## Data Availability

Not applicable.

## Supplementary Materials

The following supporting information can be downloaded at: https://www.mdpi.com/article/10.3390/molecules27196587/s1, Copies of NMR spectra, HPLC chromatograms and HRMS data after incubation in NO assay buffer, expression levels of Ptgs1 and Ptgs2 genes in genetically modified MPC cell lines, growth-rate of tumor spheroids in the presence of NO-COXIBS without radiation treatment, and detailed growth response of tumor spheroids in the presence of test compounds.

## Sample Availability

Samples of the compounds are not available from the authors.

## Appendix A. Details on NMR Analysis of Final Compounds

The applied chemical conversions starting from the hydroxymethyl to the nitroester substituted pyrazoles were found to result in characteristic NMR signal shifts in ^1^H, ^13^C, and qHSQC spectra using DMSO-*d_6_* as solvent. The hydroxymethyl-substituted compounds **3b**–**d** showed the characteristic signal shifts and coupling multiplicity of the pyrazole (~6.56 ppm, singulett), methylene (~4.51 ppm, doublett) and hydroxyl (~5.25 ppm, triplett for ^3^*J* coupling) protons. By qHSQC, the ^13^C NMR signal at 57.2 ppm could be assigned to the methylene group. Conversion to the 3-chloromethyl-4-chloropyrazoles **4b**–**d** resulted in loss of the ^1^H signal for the pyrazole ring and the hydroxyl group while the signal of the methylene group now appeared as a singulett and shifted to the downfield in ^1^H (~4.85 ppm) and upfield in ^13^C (~36.4 ppm) NMR. Finally, the NMR spectra of the nitroester derivatives **5b**–**d** were characterized by a further downfield shift of the methylene signals for both ^1^H (~5.74 ppm) and ^13^C (~66.2 ppm).
