# Supplementary material for: Exploring Nitric Oxide (NO)-Releasing Celecoxib Derivatives as Modulators of Radioresponse in Pheochromocytoma Cells
Source: Molecules. 2022 Oct 5;27(19):6587. doi: 10.3390/molecules27196587 (PMC9573605; doi:10.3390/molecules27196587)
Supplement: Supplementary file 1 [file molecules-27-06587-s001.zip › molecules-1924325-supplementary.pdf]

Supplementary Materials

Exploring nitric oxide (NO)- releasing celecoxib derivatives as modulators of radioresponse in pheochromocytoma cells

Florian Brandt [1,2,†], Martin Ullrich [1,†], Verena Seifert [1,2], Cathleen Haase-Kohn [1], Susan Richter [3], Torsten Kniess [1], Jens Pietzsch [1,2,*] and Markus Laube [1,*]

[1] Institute of Radiopharmaceutical Cancer Research, Department of Radiopharmaceutical and Chemical Biology, Helmholtz-Zentrum Dresden-Rossendorf, Bautzner Landstrasse 400, 01328 Dresden, Germany
[2] School of Science, Faculty of Chemistry and Food Chemistry, Technische Universität Dresden, Mommsenstrasse 4, 01062 Dresden, Germany
[3] Institute of Clinical Chemistry and Laboratory Medicine, University Hospital Carl Gustav Carus at the Technische Universität Dresden, Fetscherstraße 74, 01307 Dresden, Germany
* Correspondence: j.pietzsch@hzdr.de (J.P.); m.laube@hzdr.de (M.L.)
† These authors contributed equally to this work.

Table of contents

1. Copies of ^{1}H NMR and ^{13}C NMR spectra of new compounds 2

2. Copy of HPLC-HRMS chromatograms after incubation in NO-assay buffer 30

3. Expression levels of *Ptgs1* and *Ptgs2* genes in genetically modified MPC cell lines 36

4. Growth-rate of tumor spheroids in the presence of NO-COXIBS without radiation treatment ... 37

5. Detailed growth response of tumor spheroids in the presence of test compounds 38

1. Copies of ^{1}H NMR and ^{13}C NMR spectra of new compounds

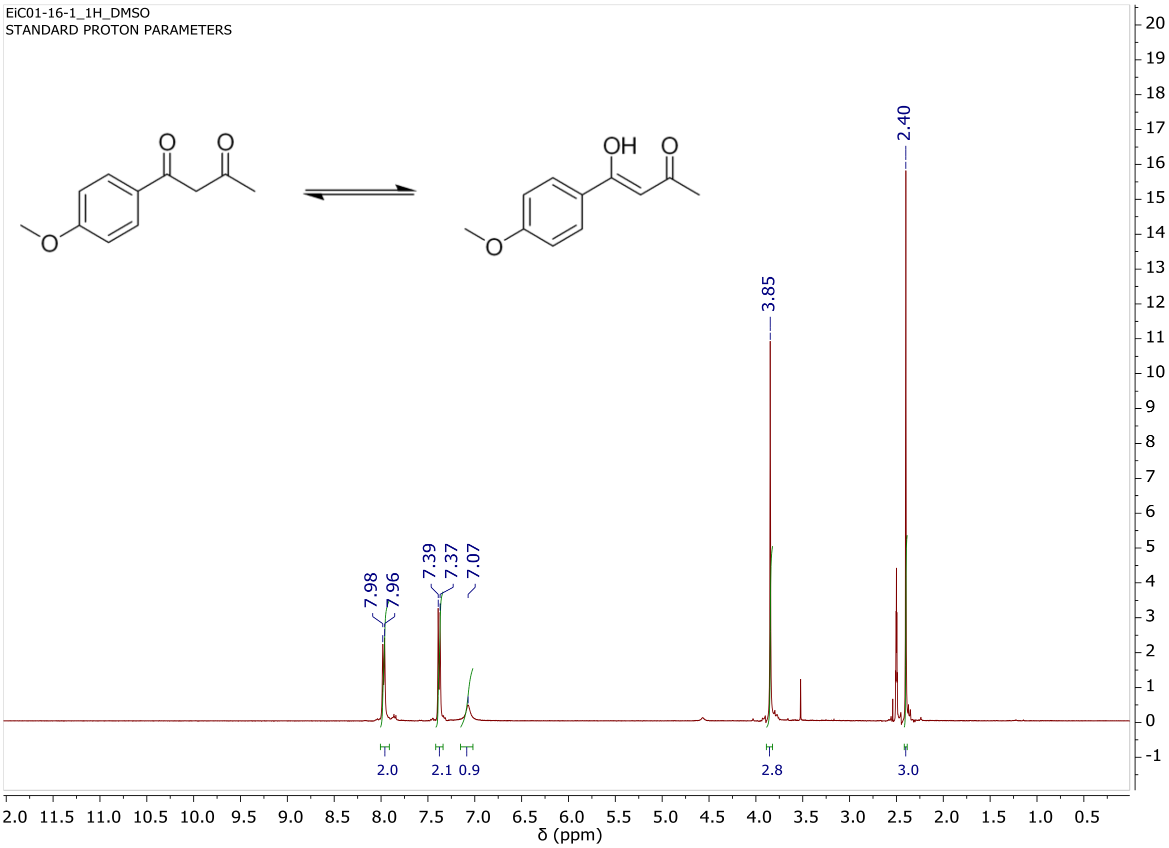

Figure S1. ^{1}H NMR spectrum of compound **1b** in DMSO-d_6

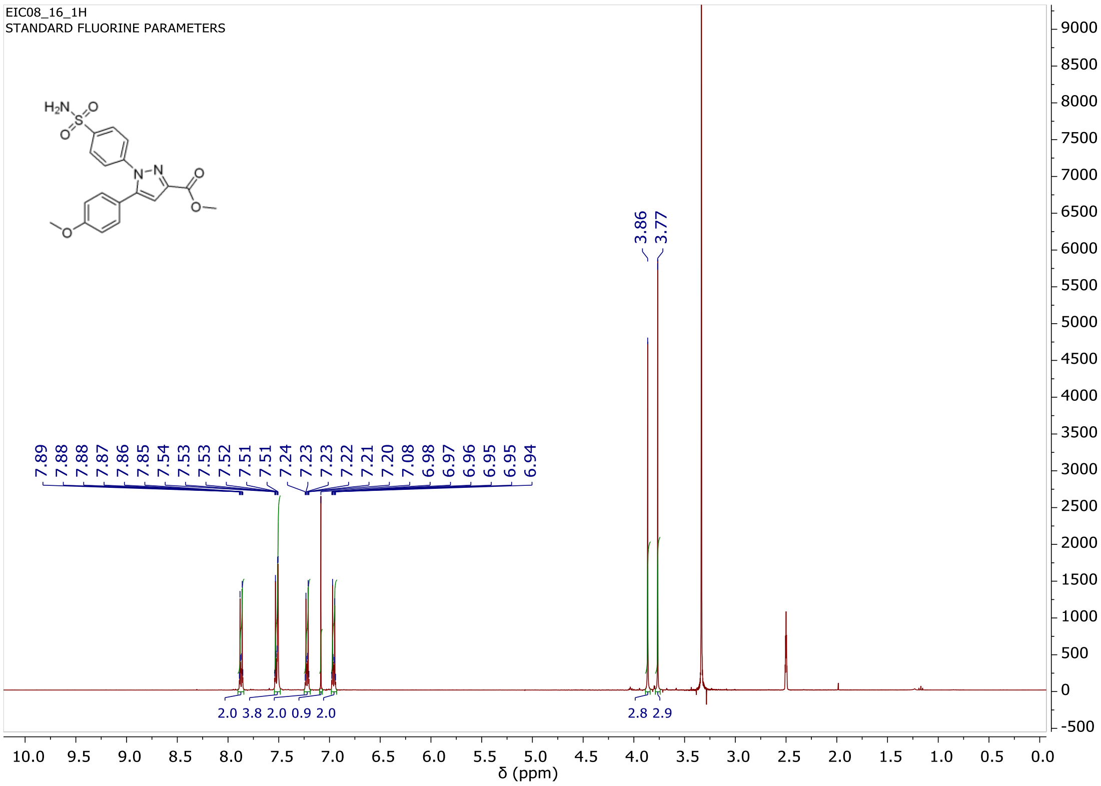

Figure S2. ^{1}H NMR spectrum of compound **2b** in DMSO-d_6

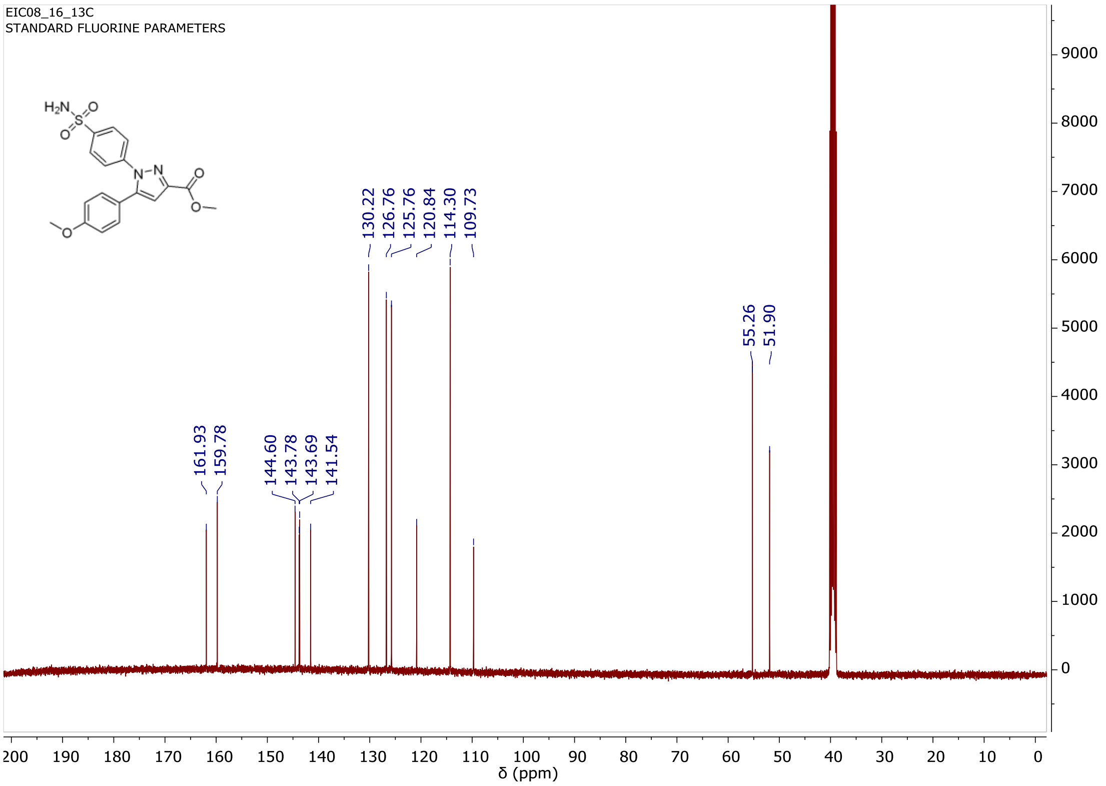

Figure S3. ^{13}C NMR spectrum of compound **2b** in DMSO-d_6

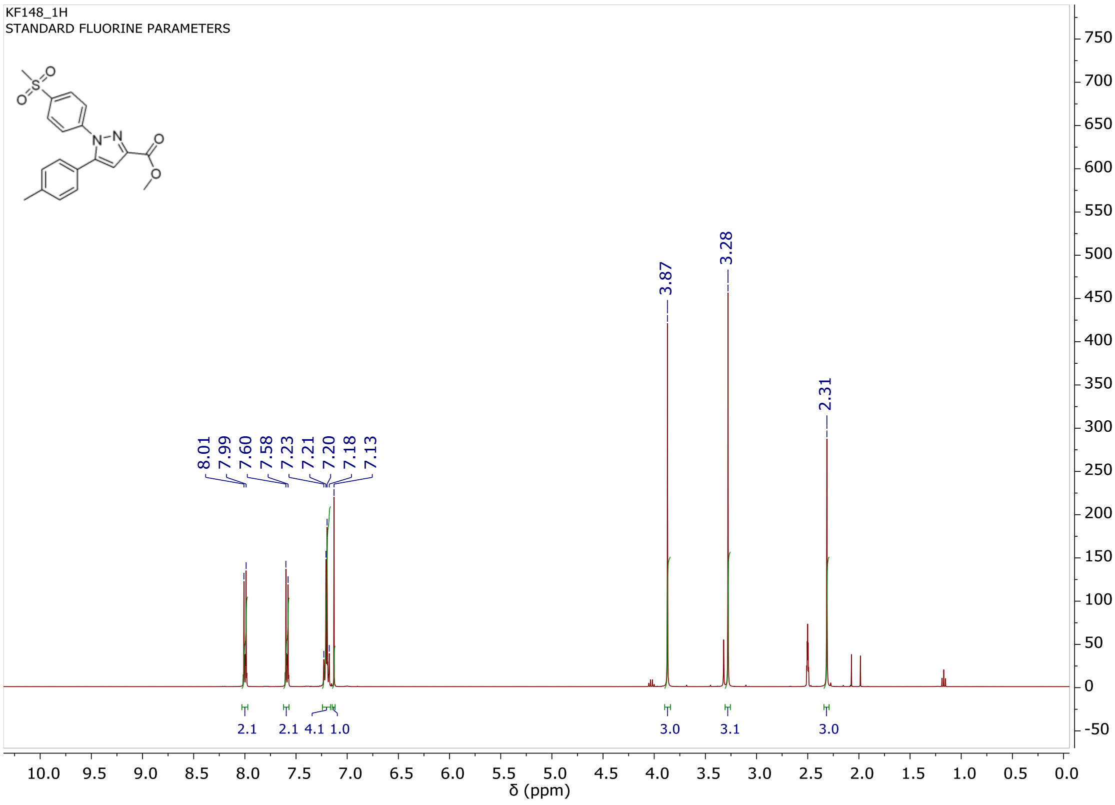

Figure S4. ^{1}H NMR spectrum of compound **2c** in DMSO-d_6

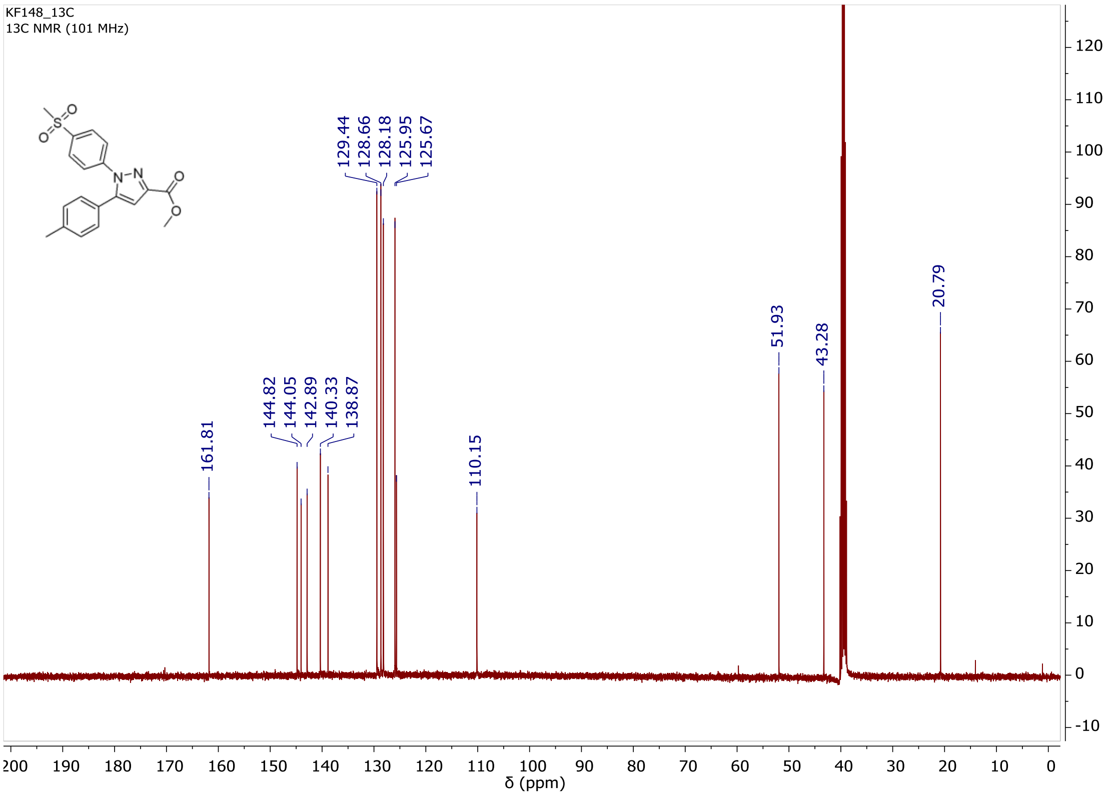

Figure S5. ^{13}C NMR spectrum of compound **2c** in DMSO-d_6

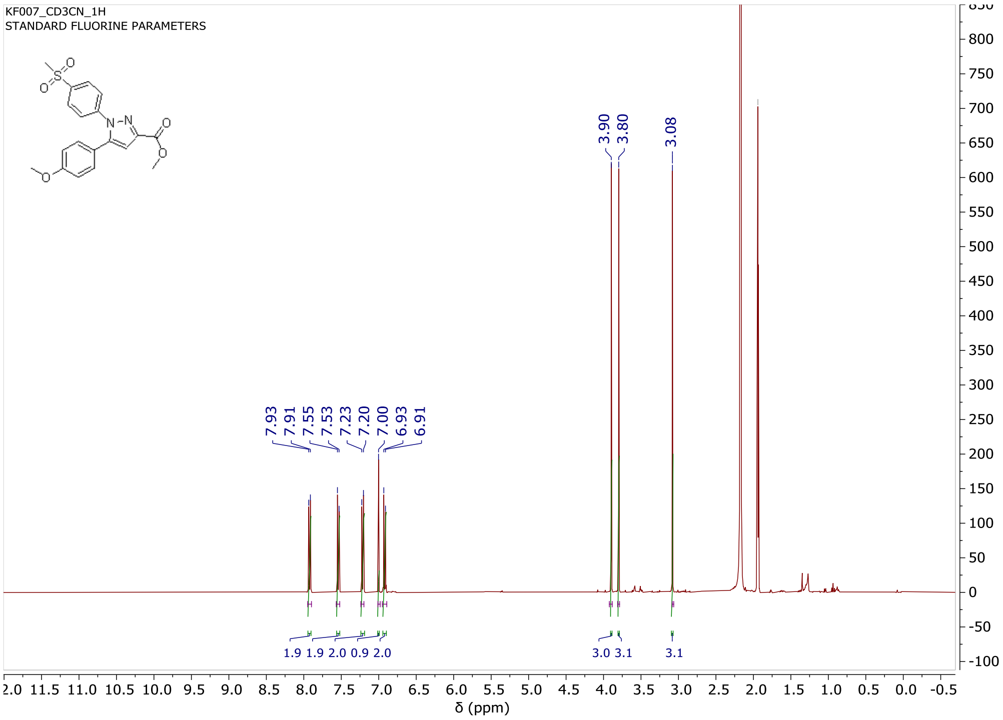

Figure S6. ^{1}H NMR spectrum of compound **2d** in CD_3CN

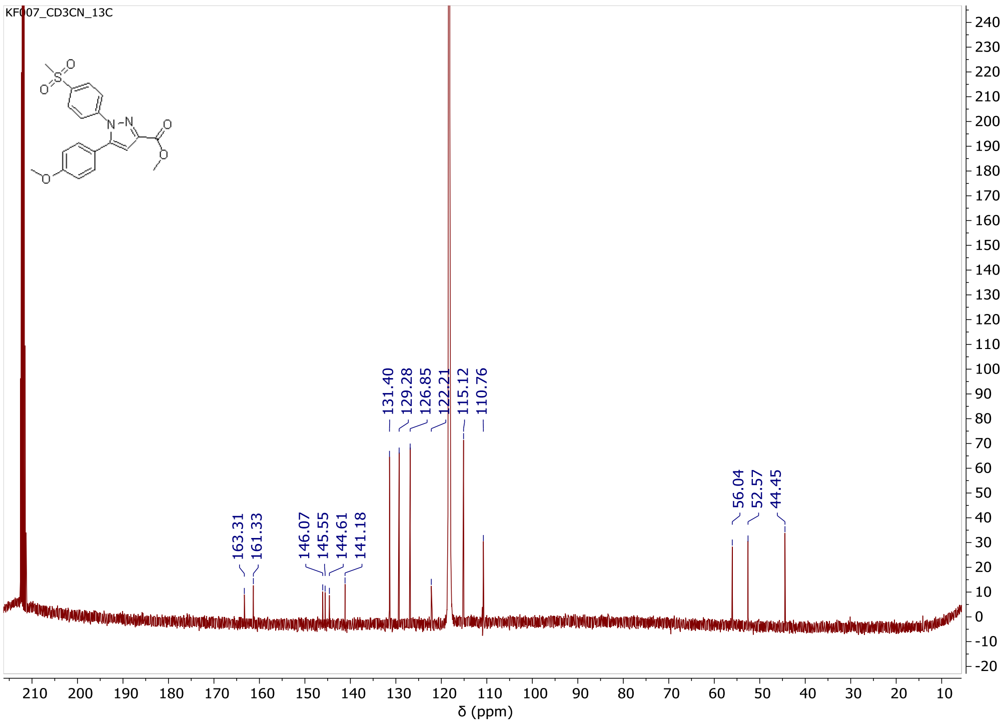

Figure S7. ^{13}C NMR spectrum of compound **2d** in CD_3CN

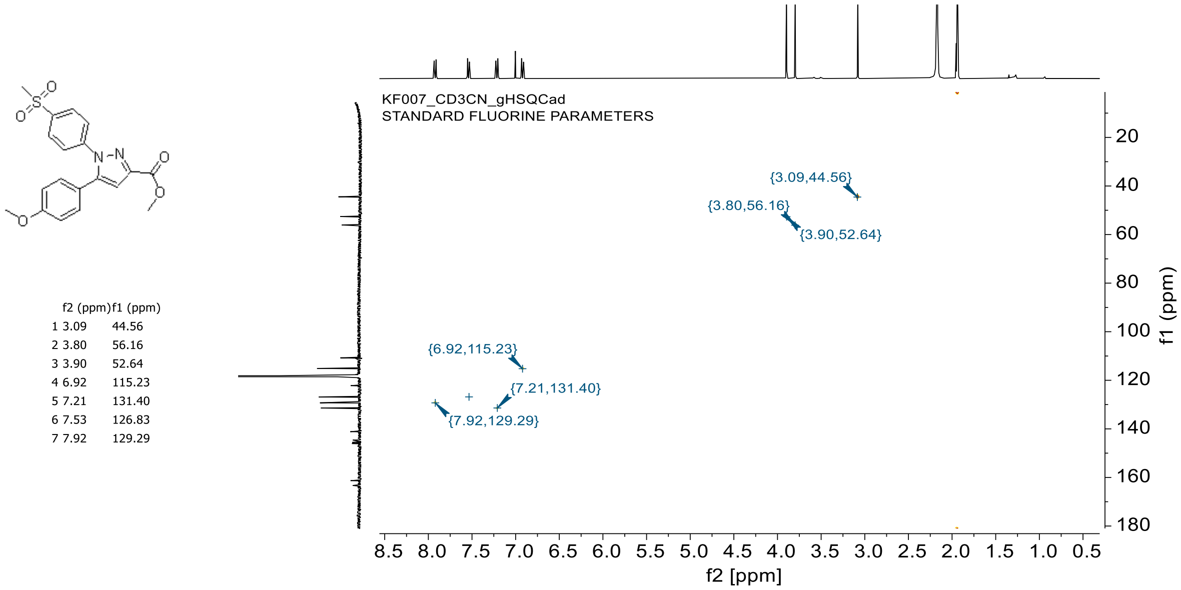

Figure S8. HSQC spectrum of compound **2d** in CD_3CN

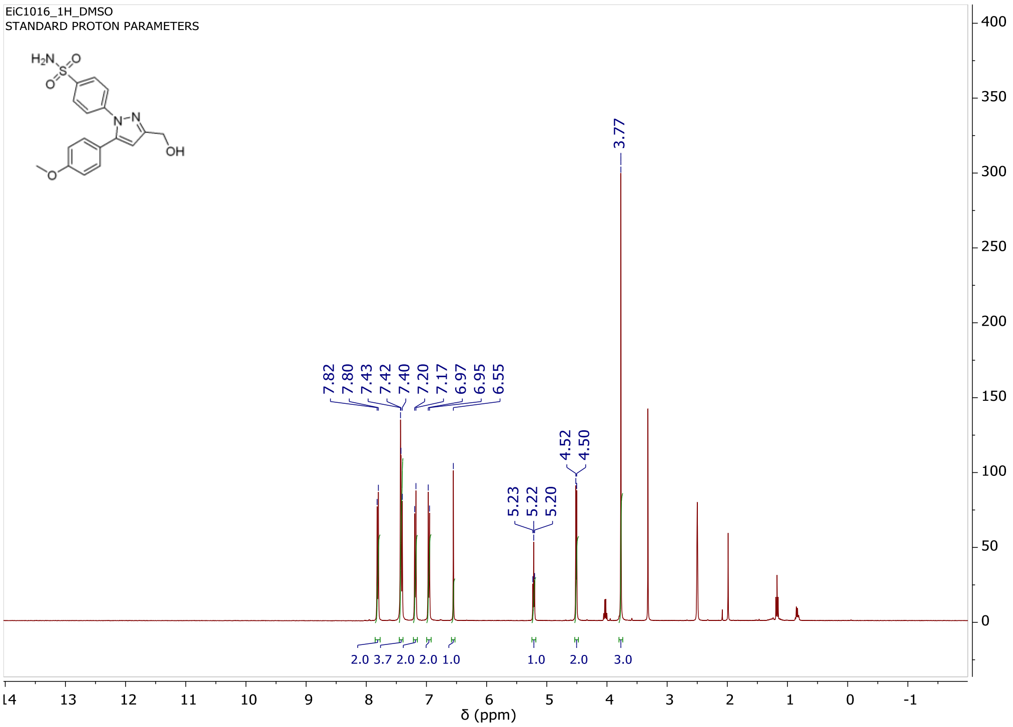

Figure S9. ^{1}H NMR spectrum of compound **3b** in DMSO-d_6

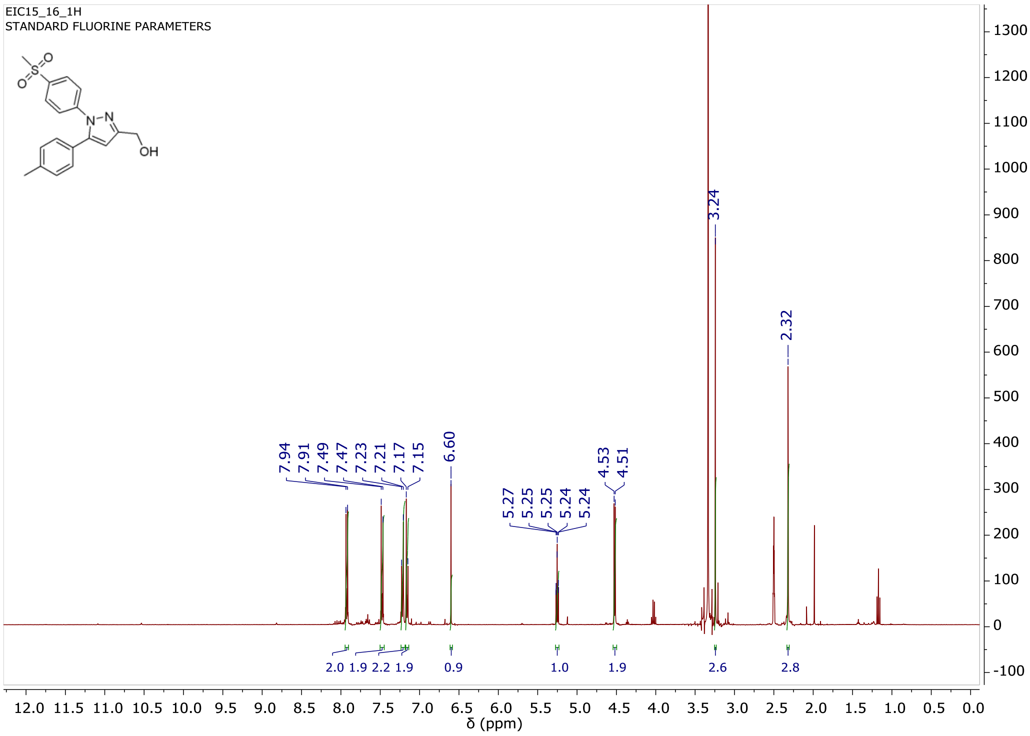

Figure S10. ^{1}H NMR spectrum of compound **3c** in DMSO-d_6

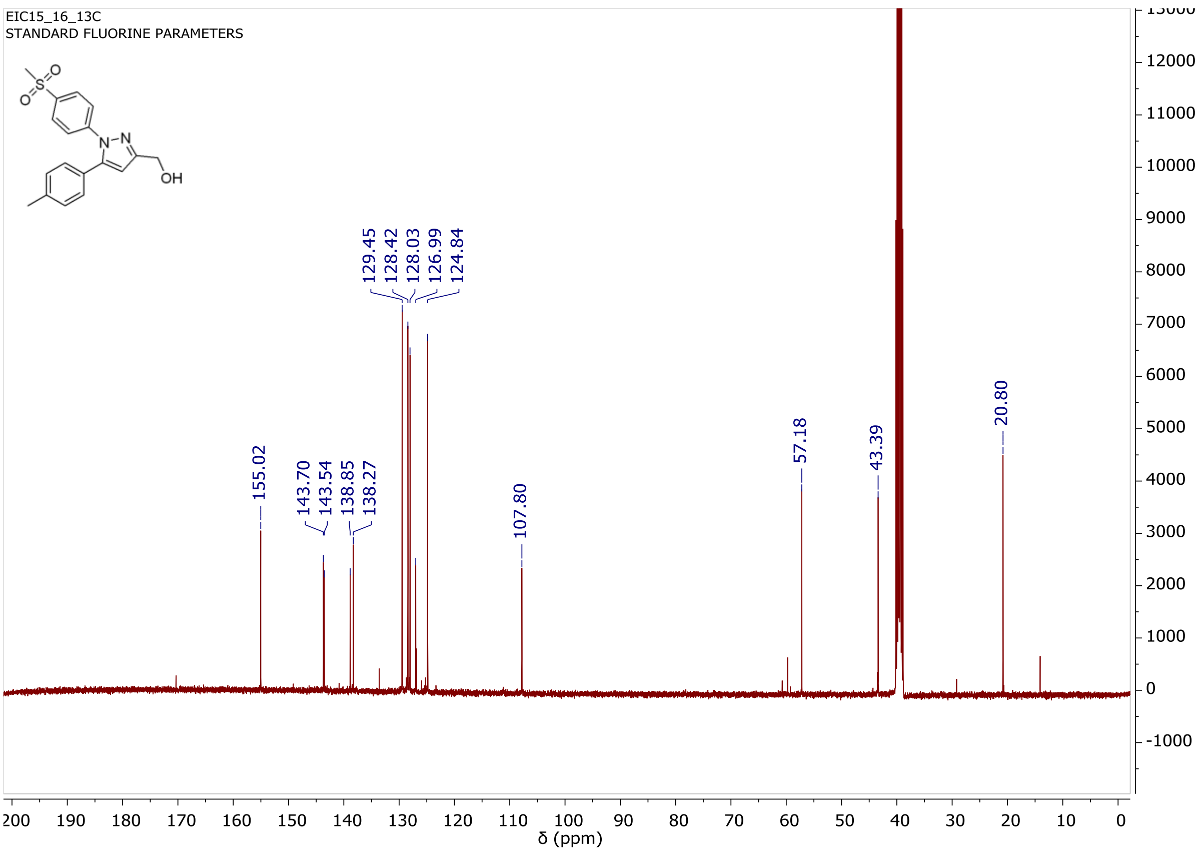

Figure S11. ^{13}C NMR spectrum of compound **3c** in DMSO-d_6

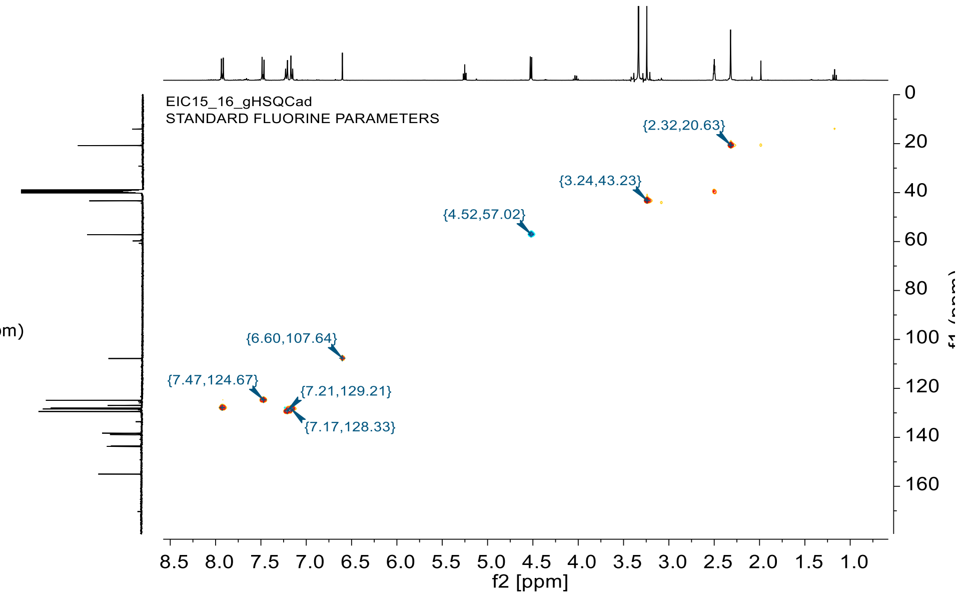

	f1 (ppm)	f2 (ppm)
1	129.21	7.21
2	128.33	7.17
3	127.85	7.93
4	124.67	7.47
5	107.64	6.60
6	57.02	4.52
7	43.23	3.24
8	20.63	2.32

Figure S12. HSQC spectrum of compound **3c** in DMSO-d_6

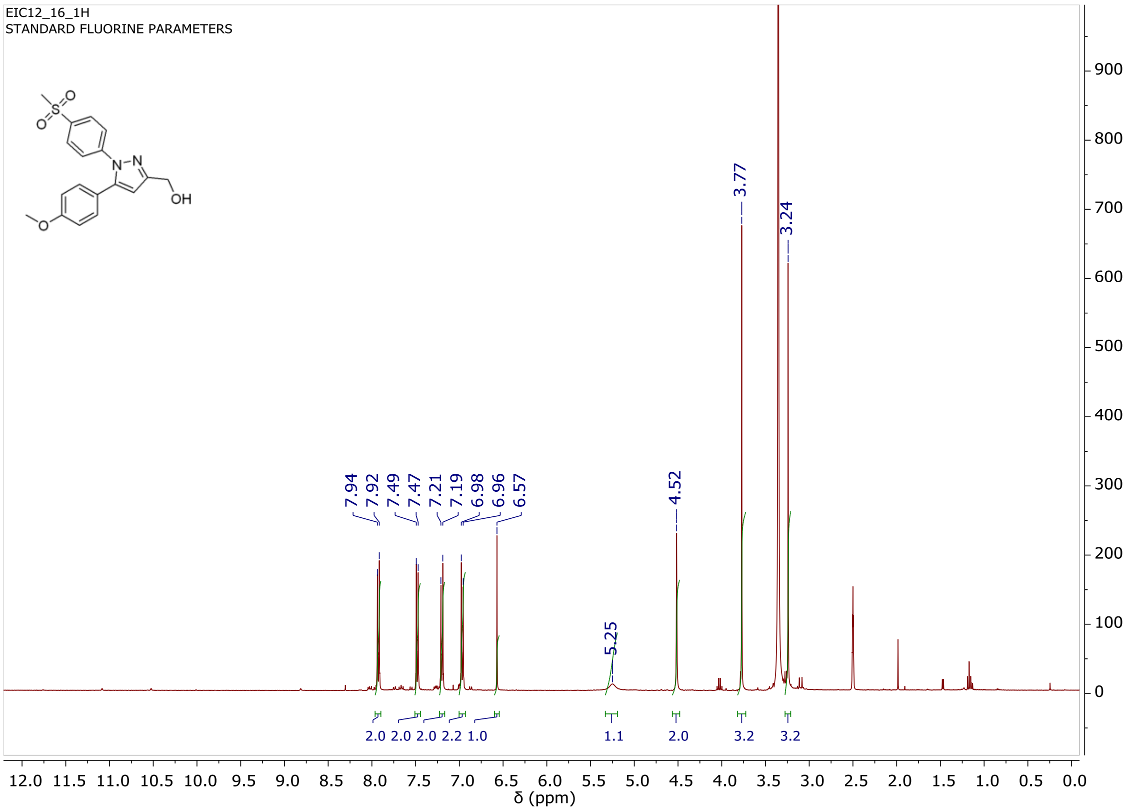

Figure S13. ^{1}H NMR spectrum of compound **3d** in DMSO-d_6

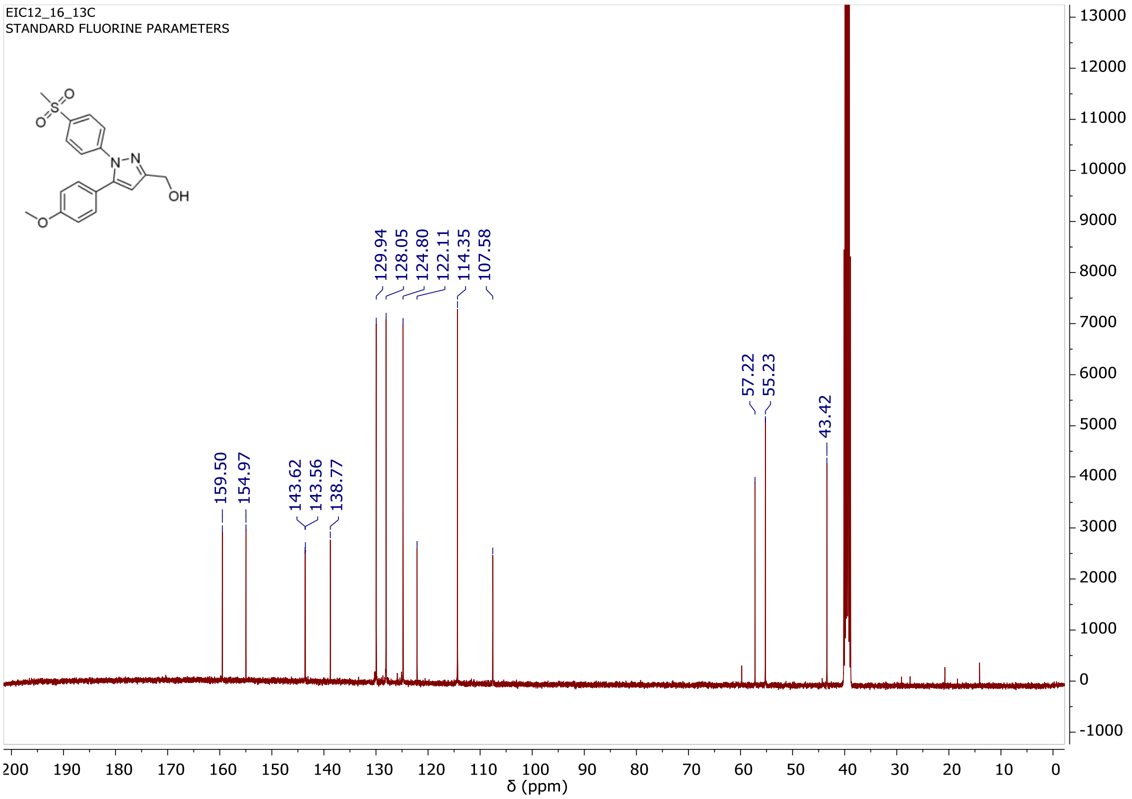

Figure S14. ^{13}C NMR spectrum of compound **3d** in DMSO-d_6

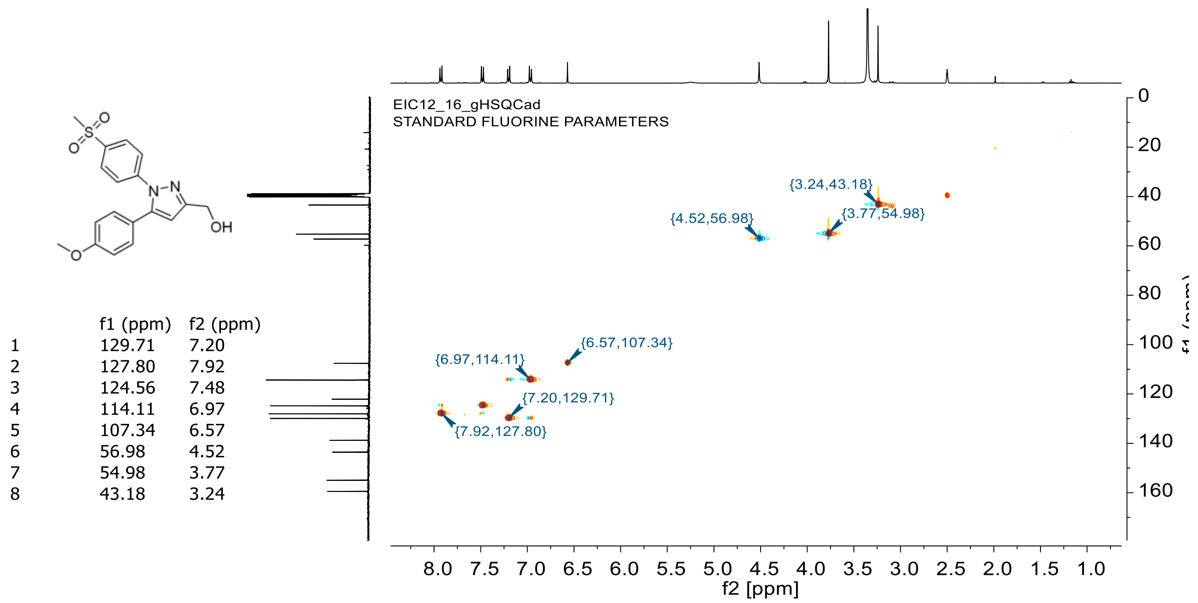

	f1 (ppm)	f2 (ppm)
1	129.71	7.20
2	127.80	7.92
3	124.56	7.48
4	114.11	6.97
5	107.34	6.57
6	56.98	4.52
7	54.98	3.77
8	43.18	3.24

Figure S15. HSQC spectrum of compound **3d** in DMSO-d_6

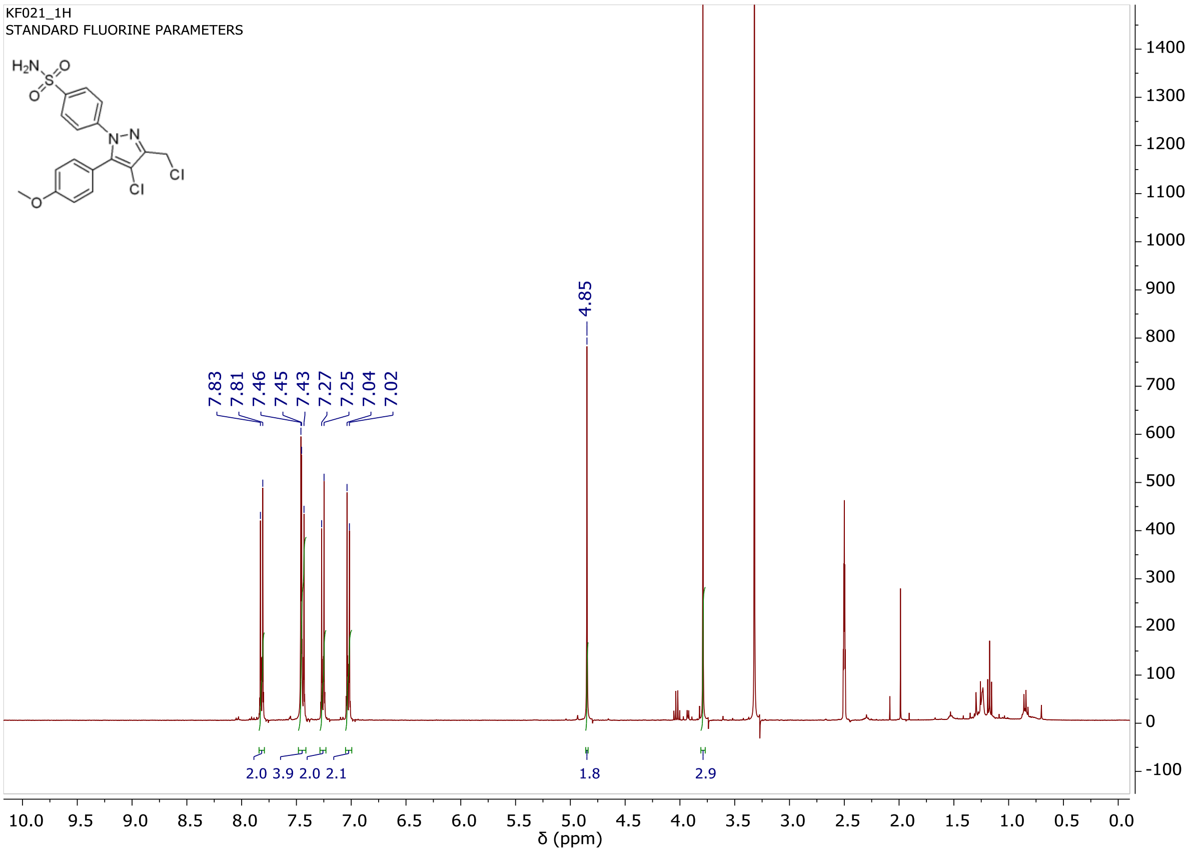

Figure S16. ^1H NMR spectrum of compound **4b** in DMSO-d_6

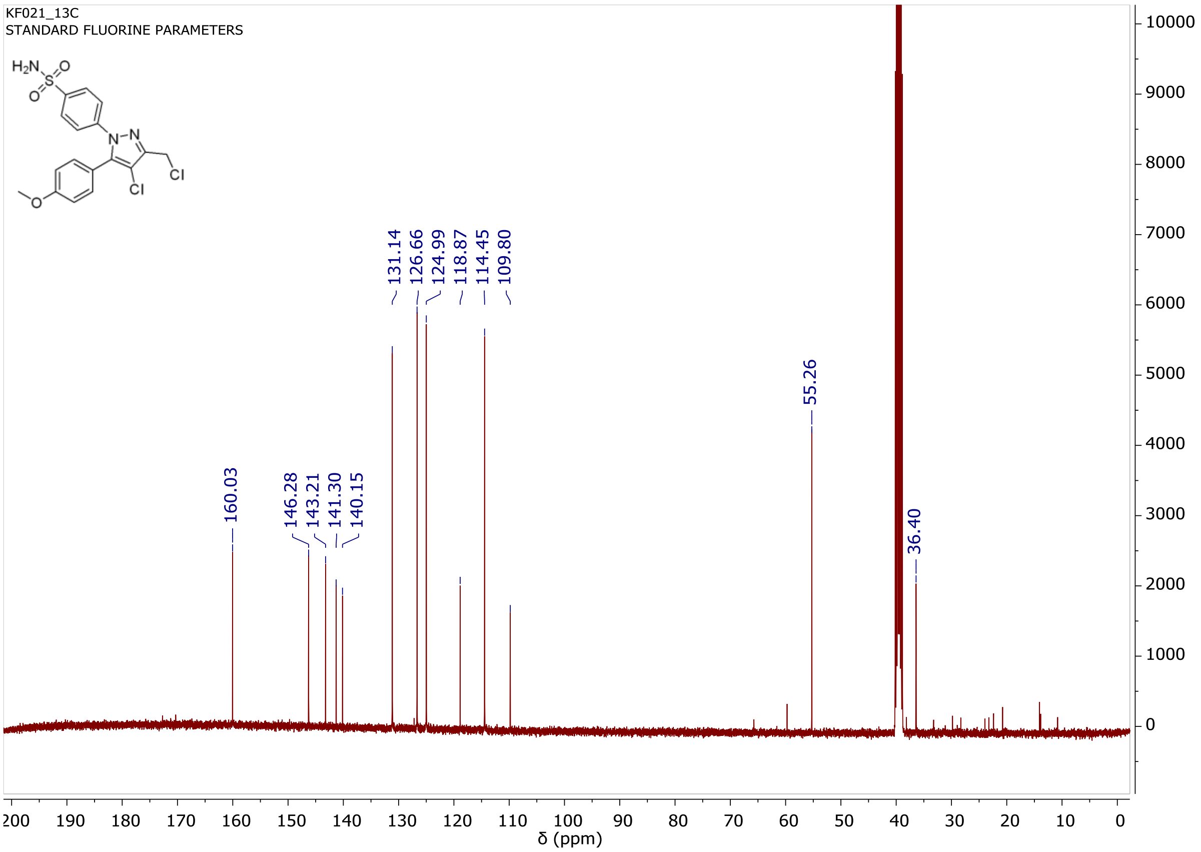

Figure S17. ^{13}C NMR spectrum of compound **4b** in DMSO-d_6

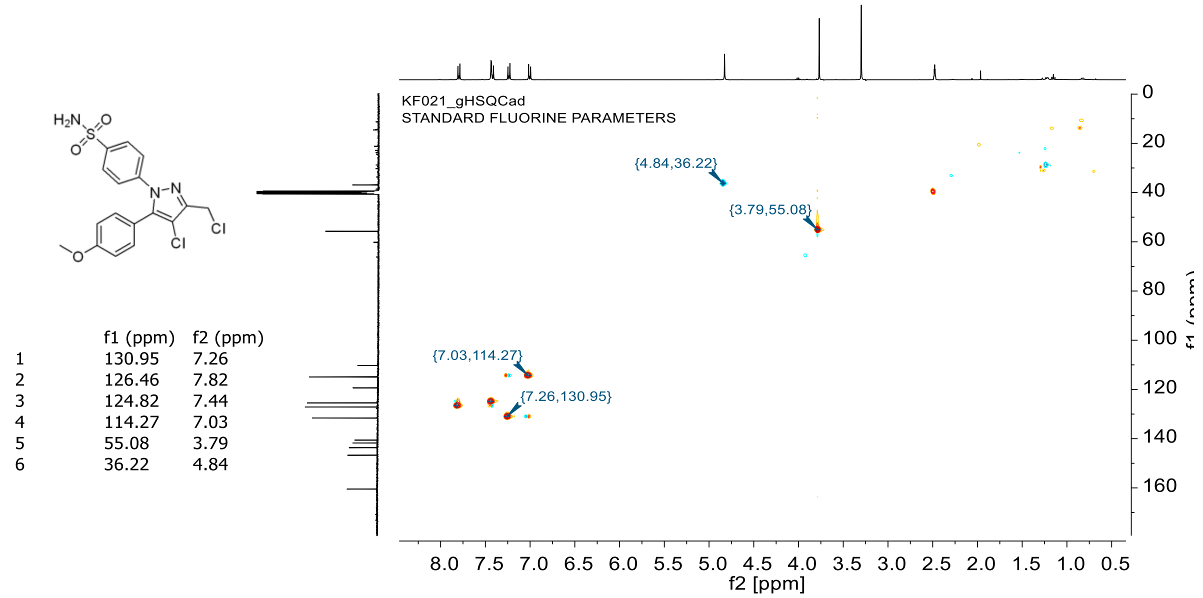

Figure S18. HSQC spectrum of compound **4b** in DMSO-d_6

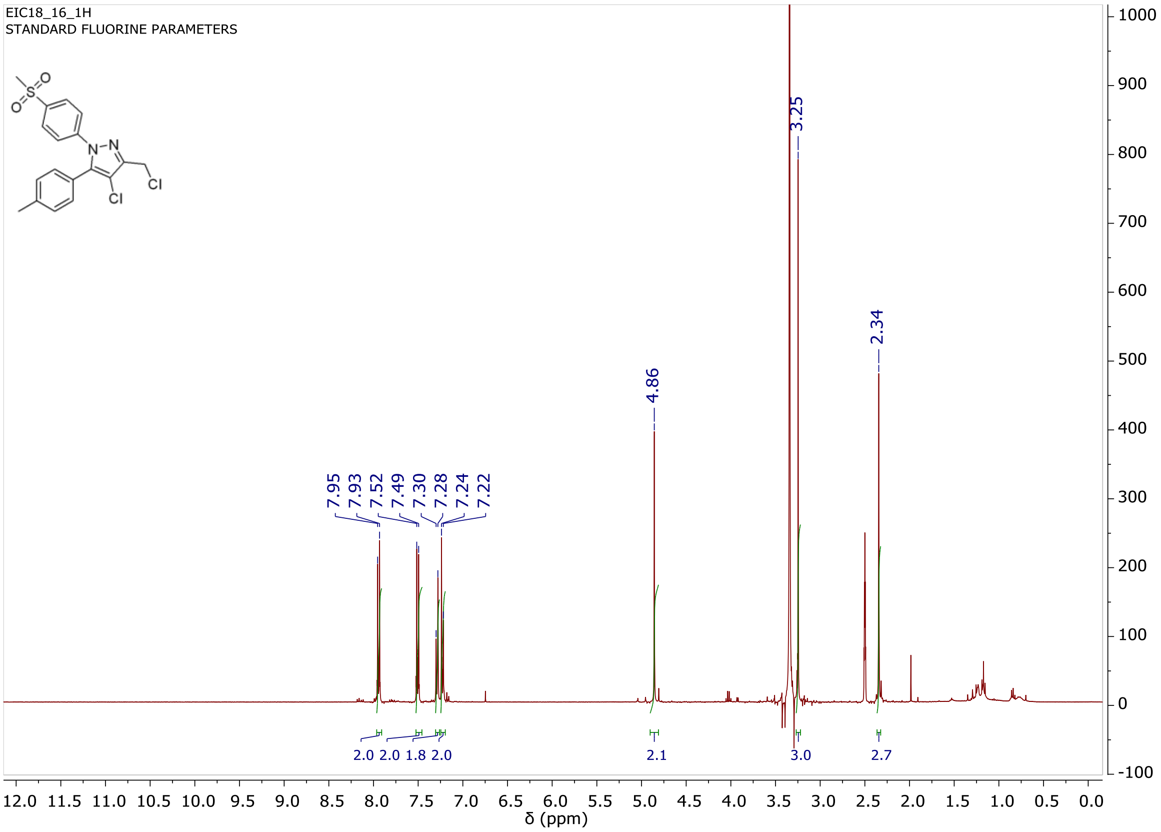

Figure S19. ^{1}H NMR spectrum of compound **4c** in DMSO-d_6

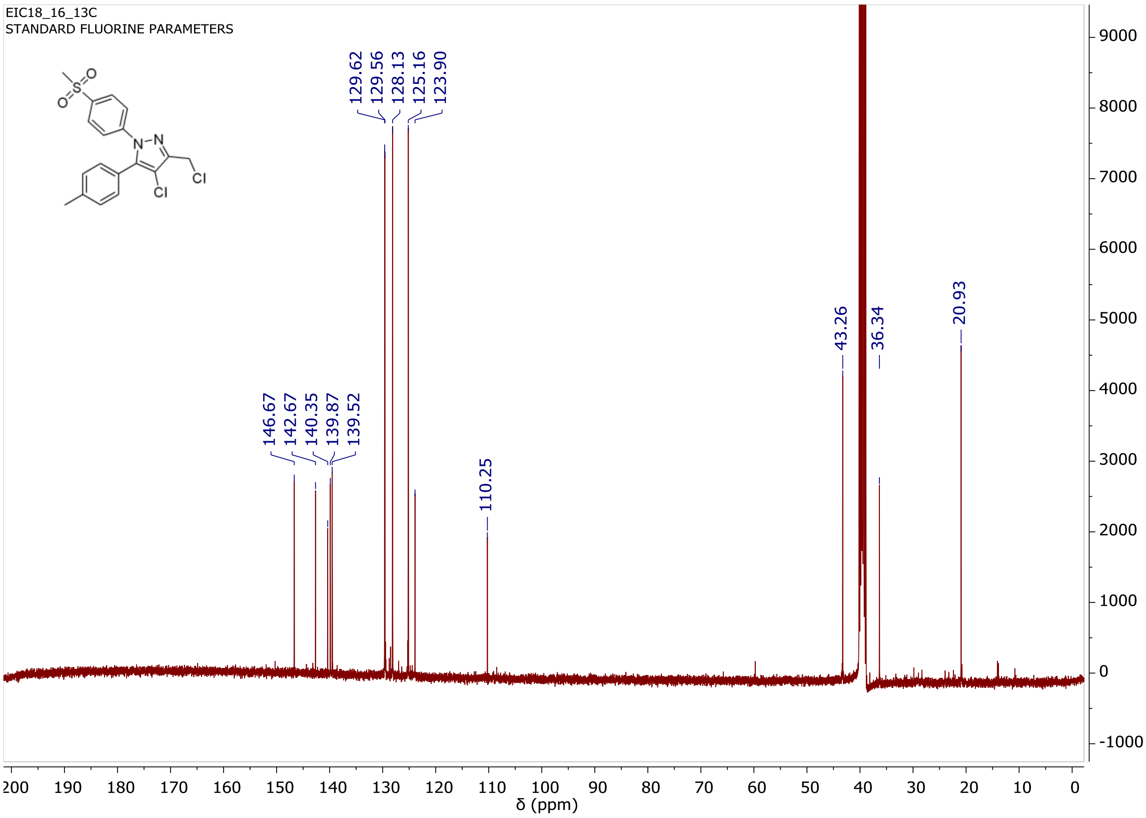

Figure S20. ^{13}C NMR spectrum of compound **4c** in DMSO-d_6

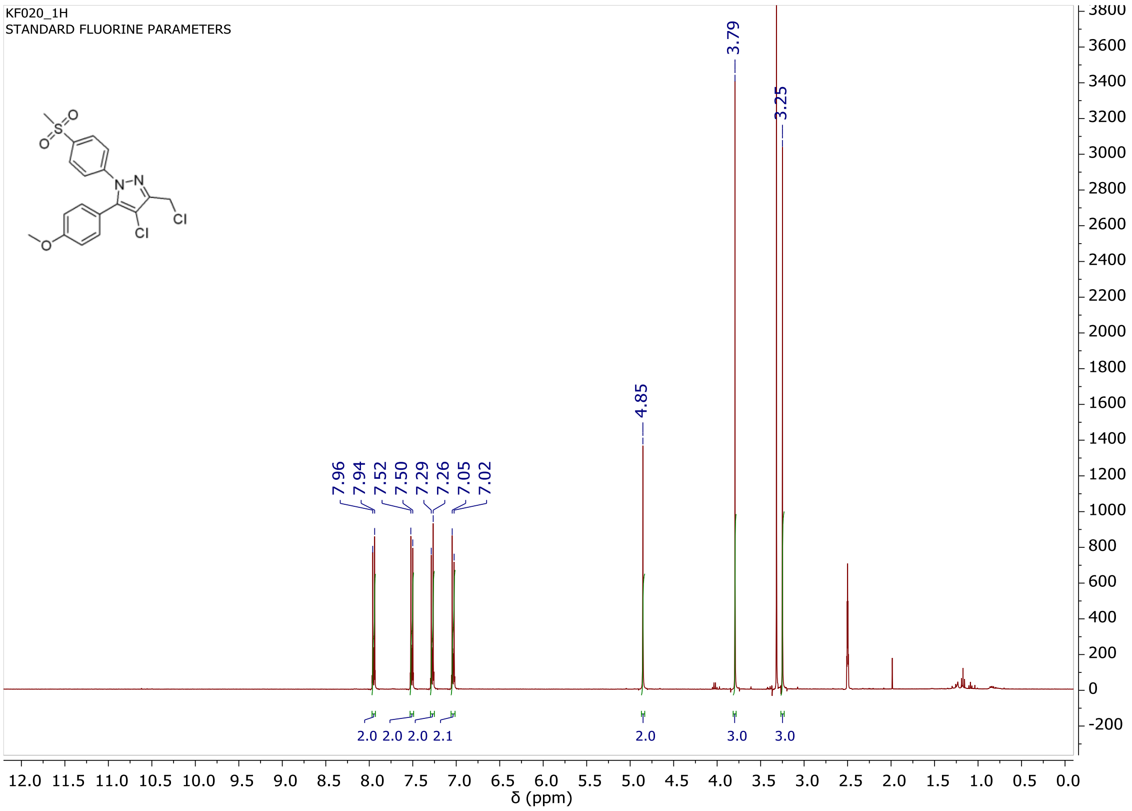

Figure S21. ^{1}H NMR spectrum of compound **4d** in DMSO-d_6

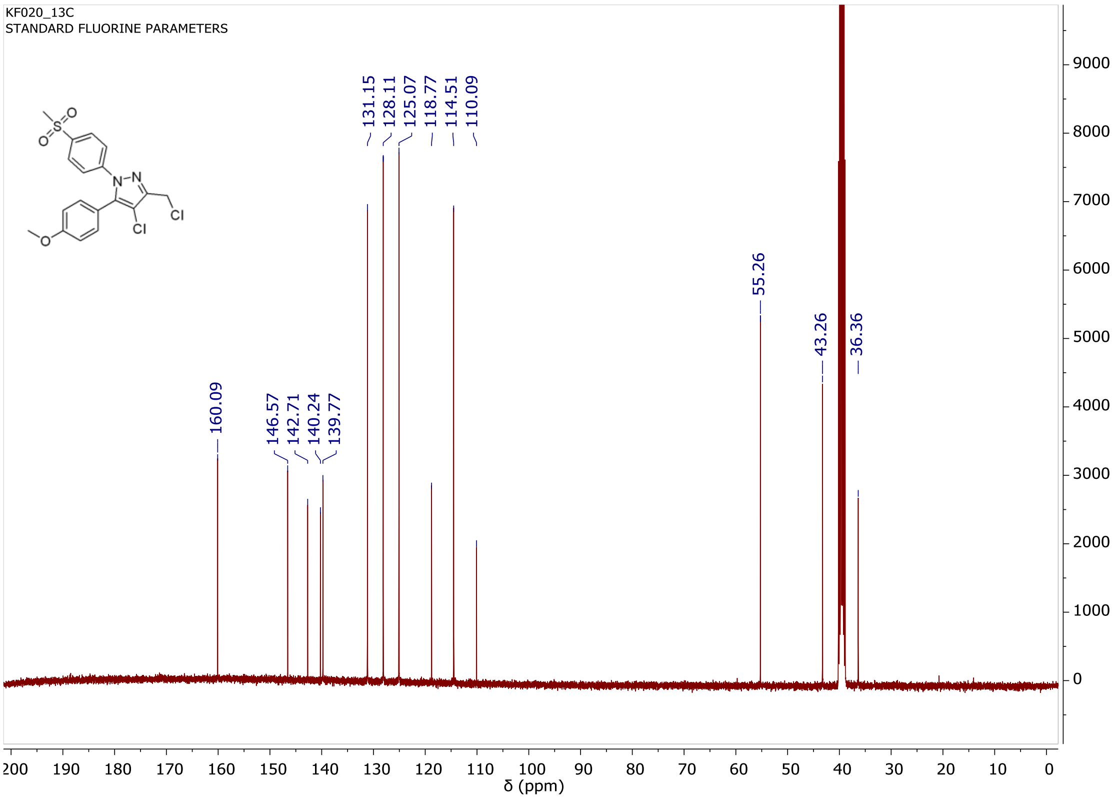

Figure S22. ^{13}C NMR spectrum of compound **4d** in DMSO-d_6

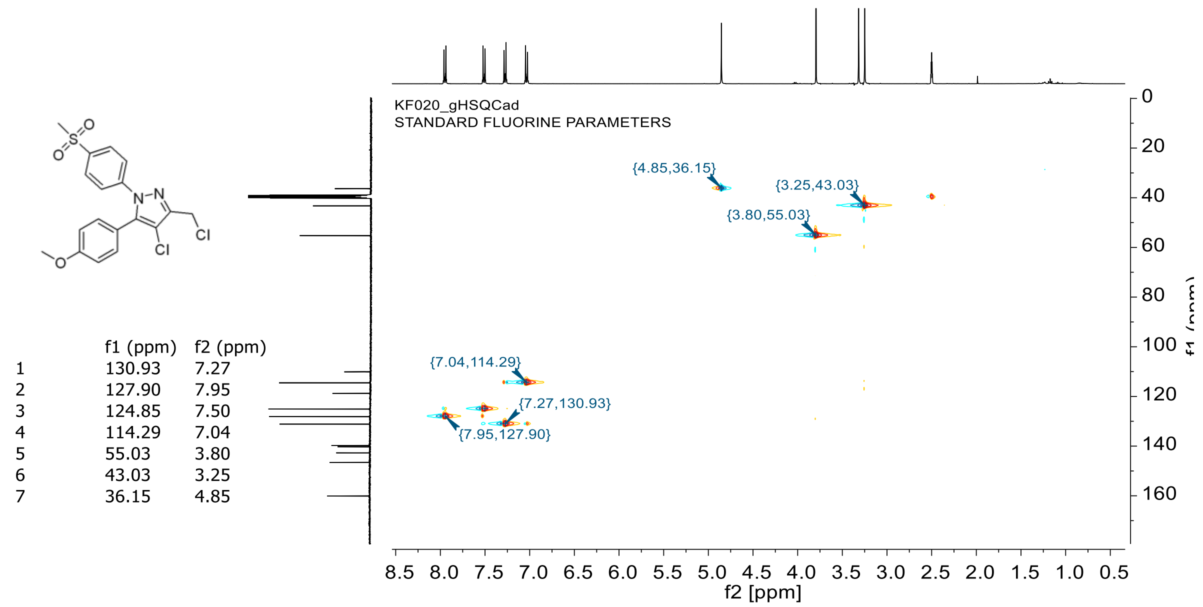

Figure S23. HSQC spectrum of compound **4d** in DMSO-d_6

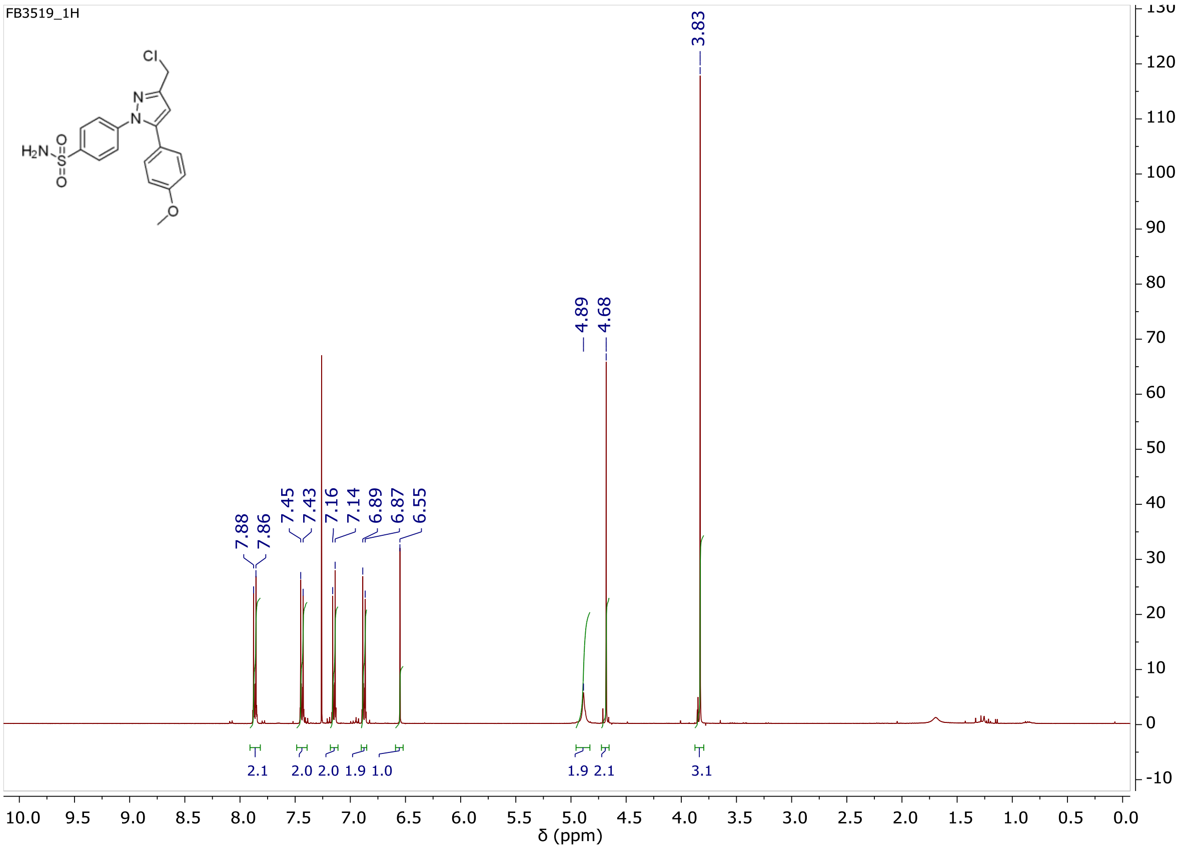

Figure S24. ^{1}H NMR spectrum of compound **4f** in $CDCl_3$

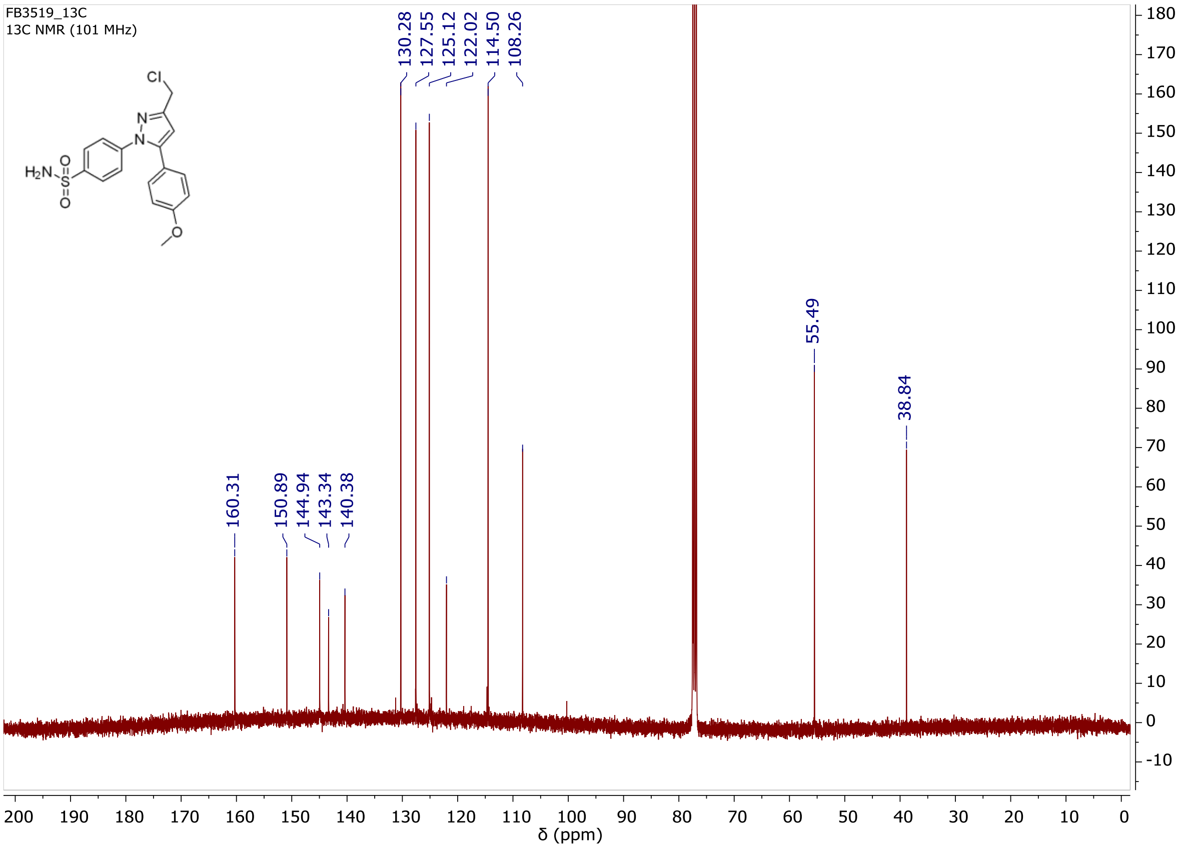

Figure S25. ^{13}C NMR spectrum of compound **4f** in $CDCl_3$

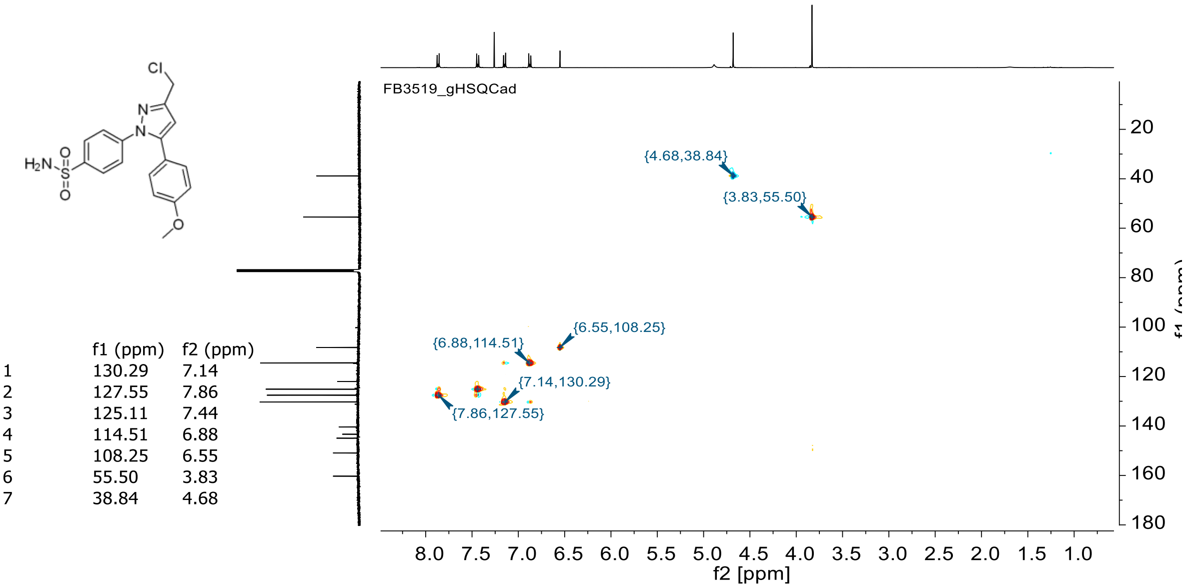

	f1 (ppm)	f2 (ppm)
1	130.29	7.14
2	127.55	7.86
3	125.11	7.44
4	114.51	6.88
5	108.25	6.55
6	55.50	3.83
7	38.84	4.68

Figure S26. HSQC spectrum of compound **4f** in $CDCl_3$

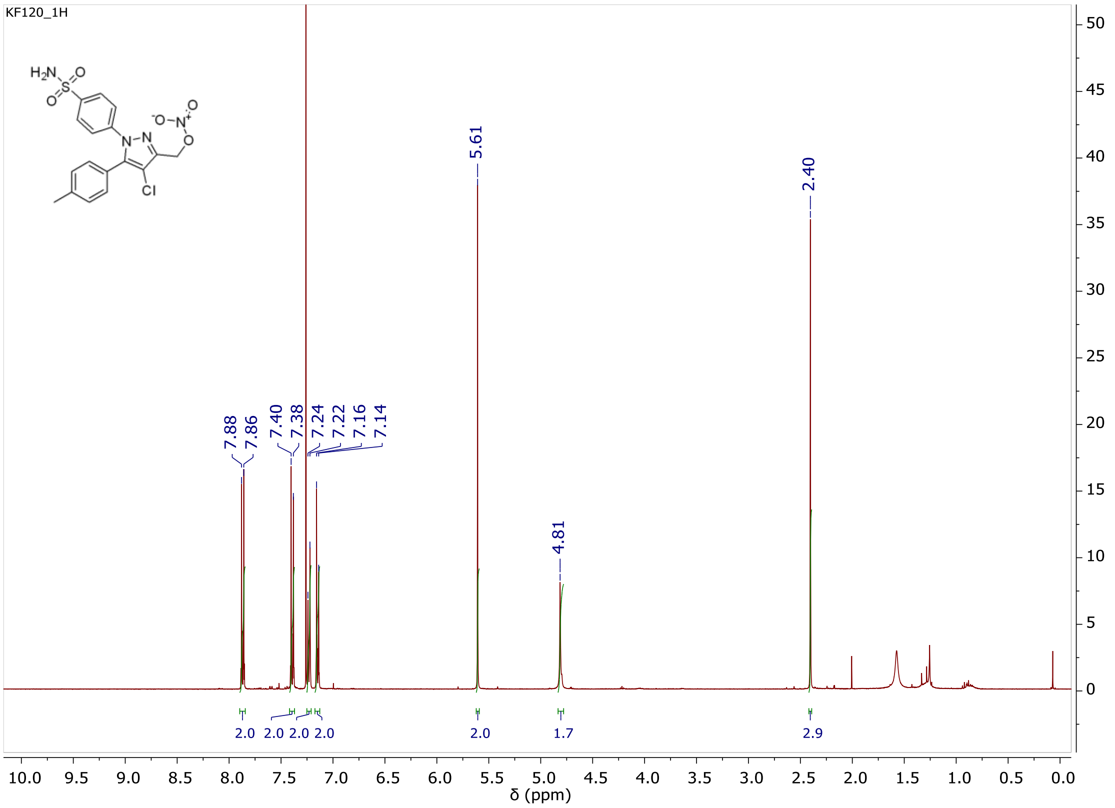

Figure S27. ^{1}H NMR spectrum of compound **5a** in $CDCl_3$

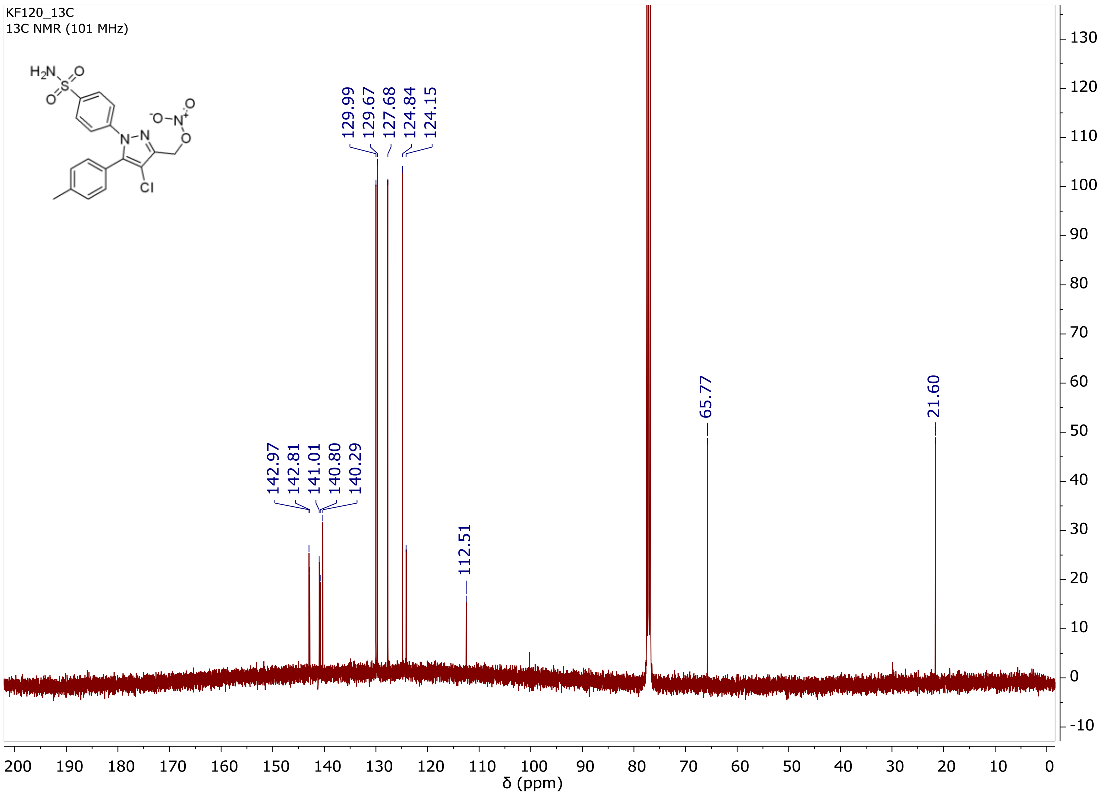

Figure S28. ^{13}C NMR spectrum of compound **5a** in $CDCl_3$

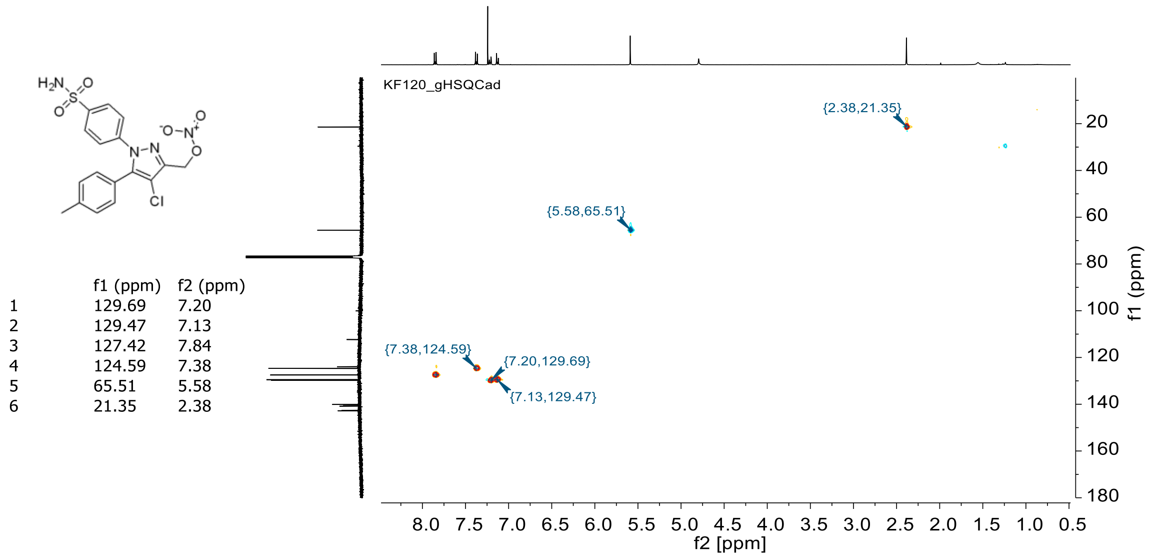

Figure S29. HSQC spectrum of compound **5a** in $CDCl_3$

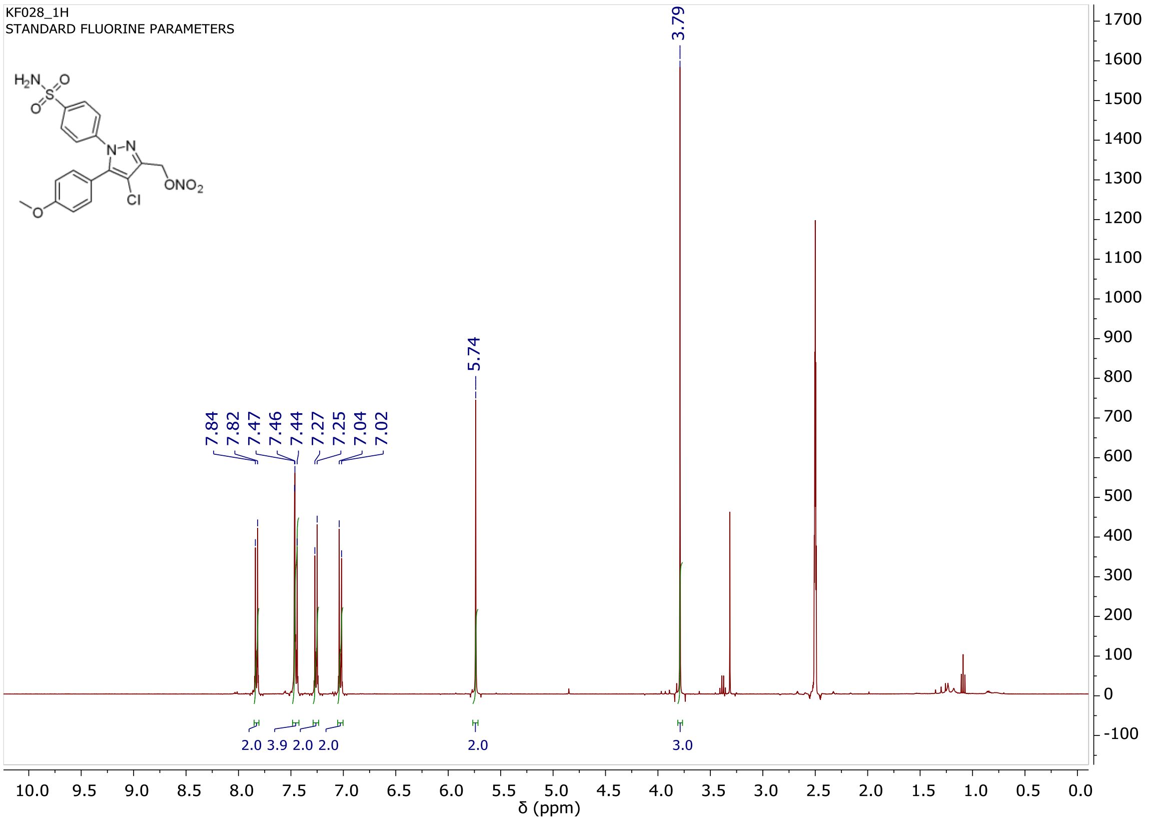

Figure S30. ^1H NMR spectrum of compound **5b** in DMSO-d_6

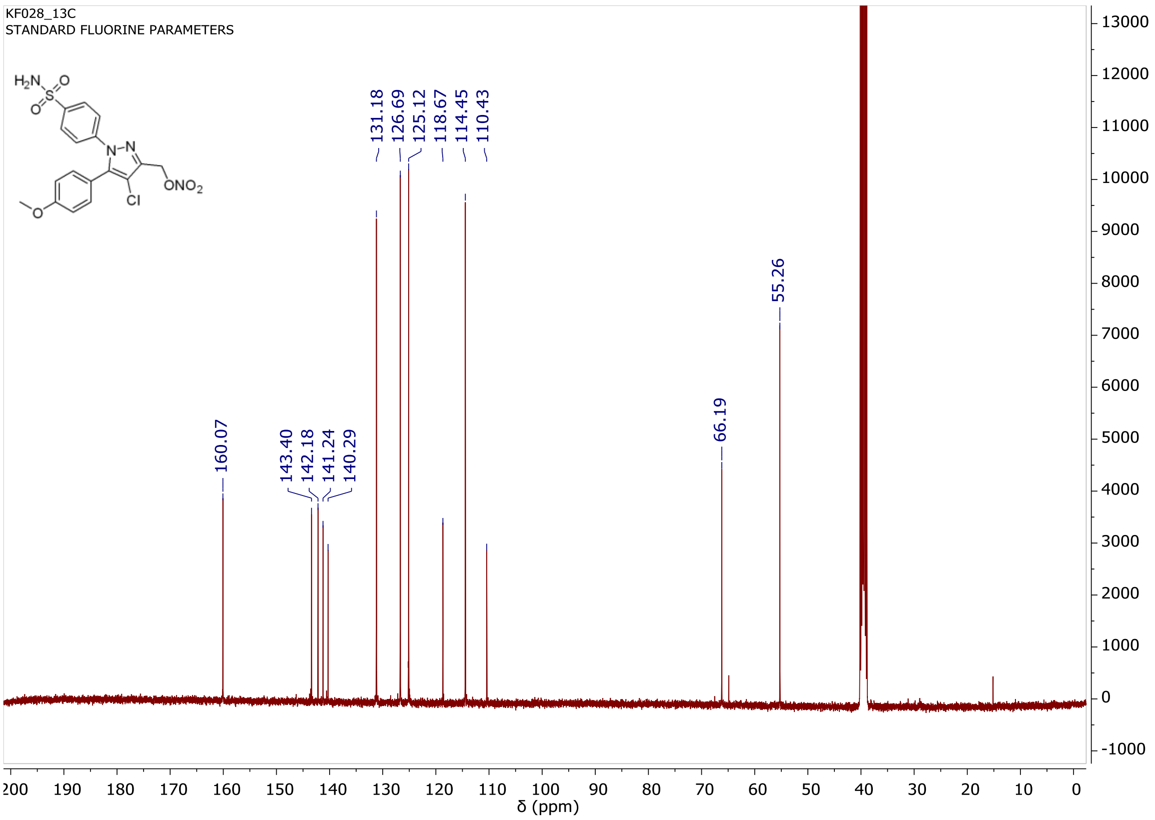

Figure S31. ^{13}C NMR spectrum of compound **5b** in DMSO-d_6

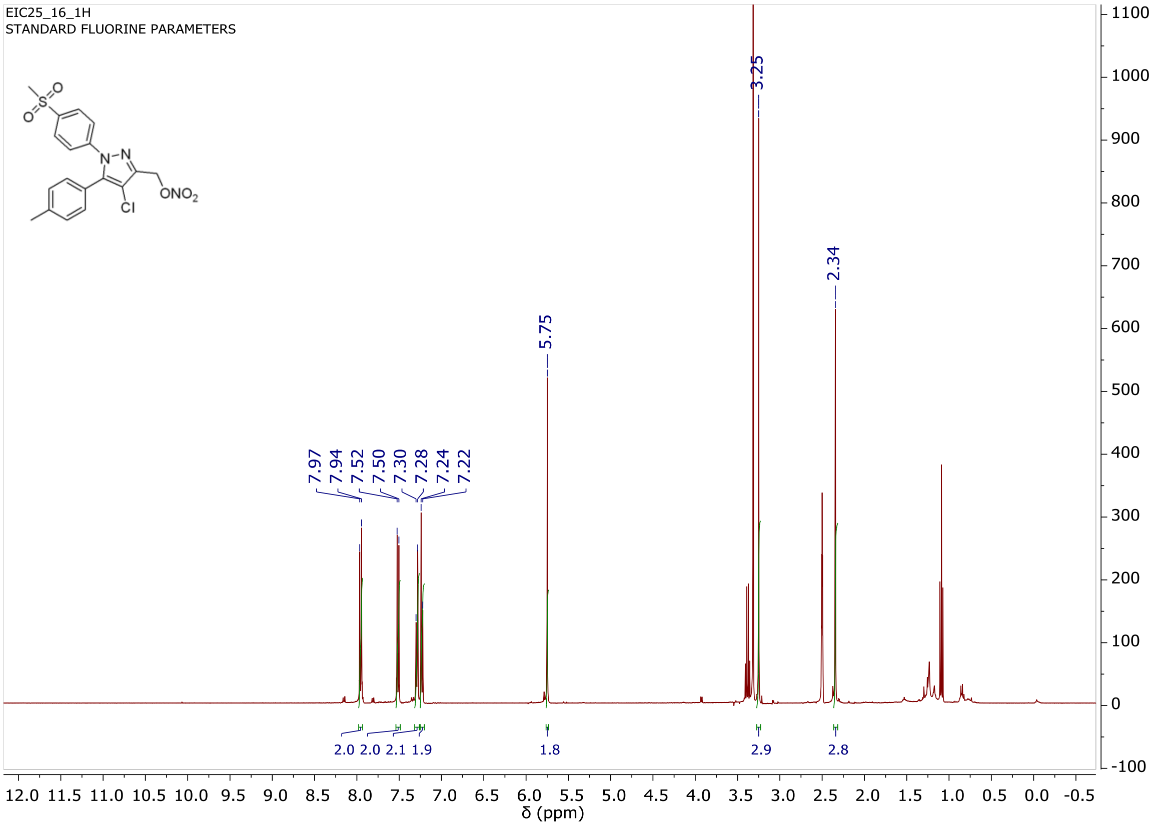

Figure S32. ^{1}H NMR spectrum of compound **5c** in DMSO-d_6

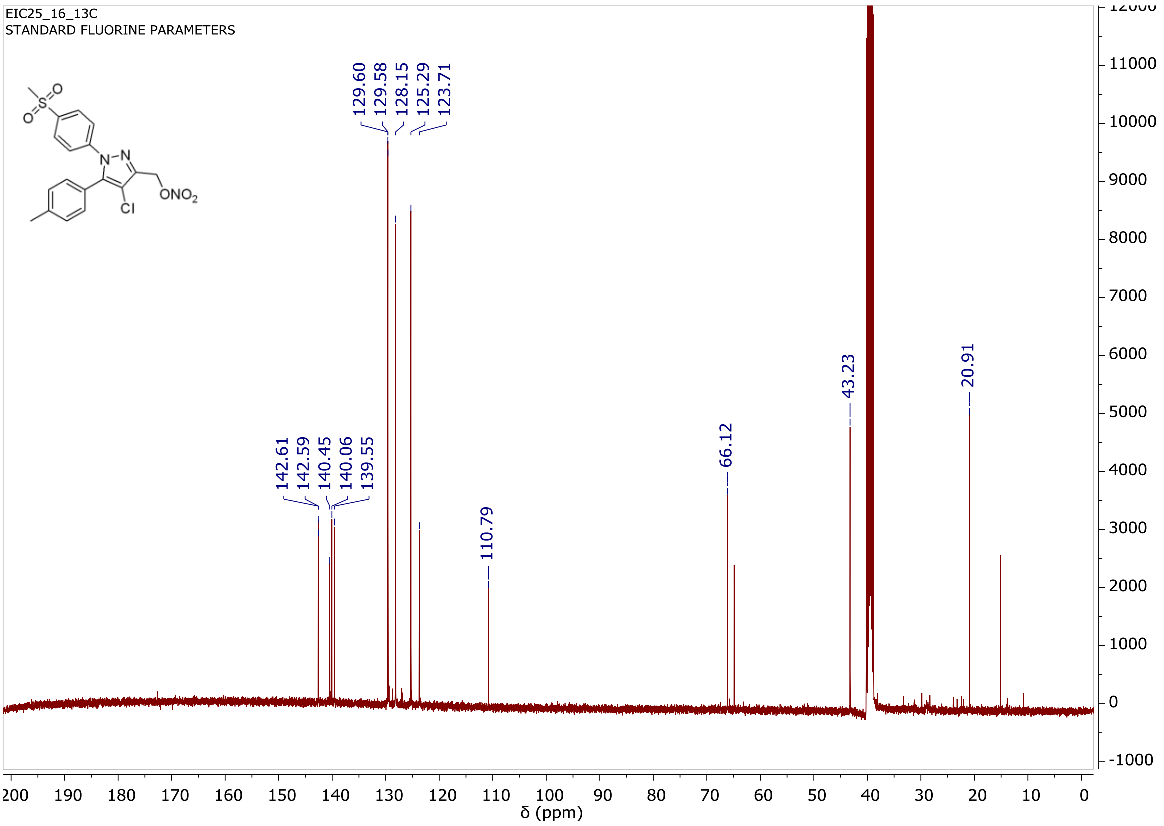

Figure S33. ^{13}C NMR spectrum of compound **5c** in DMSO-d_6

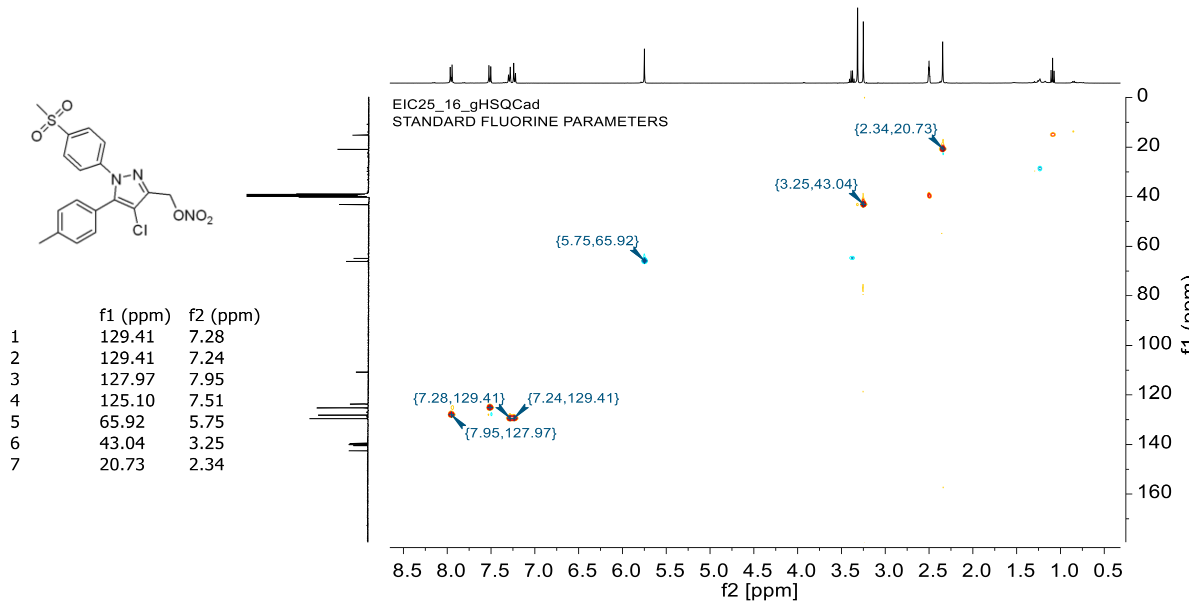

	f1 (ppm)	f2 (ppm)
1	129.41	7.28
2	129.41	7.24
3	127.97	7.95
4	125.10	7.51
5	65.92	5.75
6	43.04	3.25
7	20.73	2.34

Figure S34. HSQC spectrum of compound **5c** in DMSO-d_6

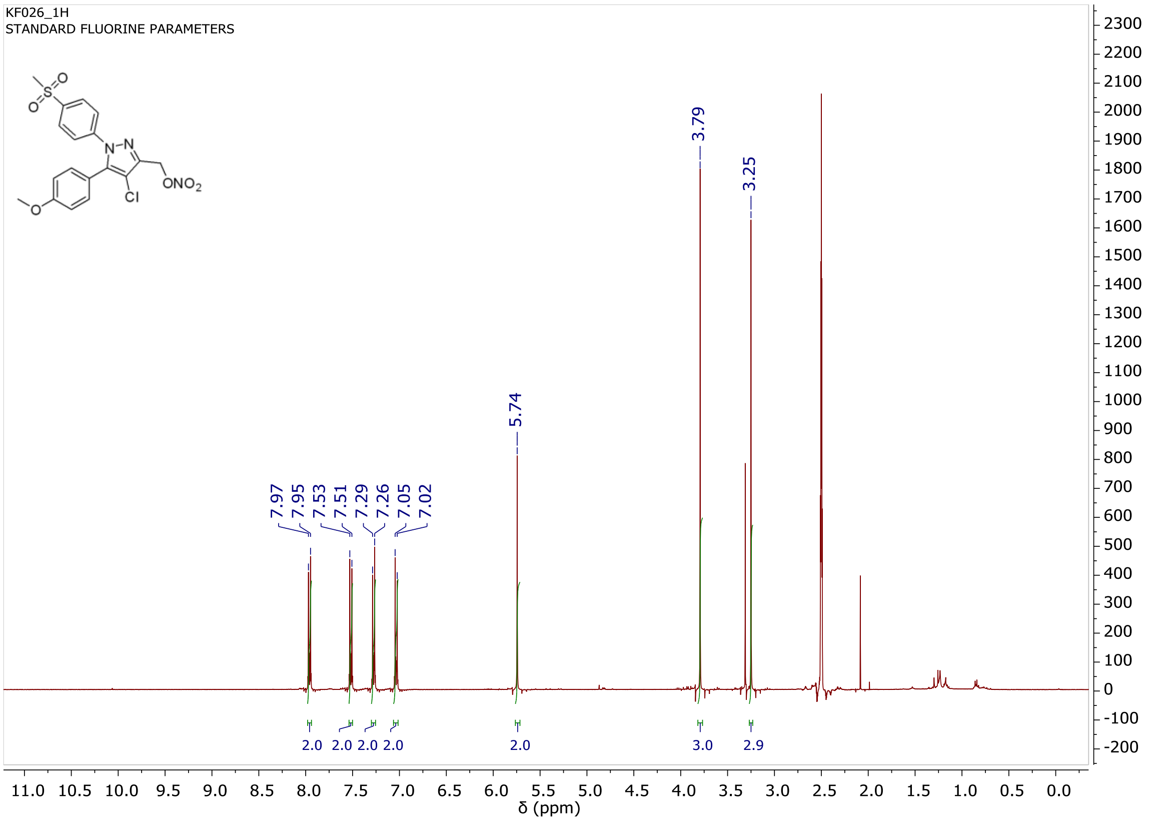

Figure S35. ^{1}H NMR spectrum of compound **5d** in DMSO-d_6

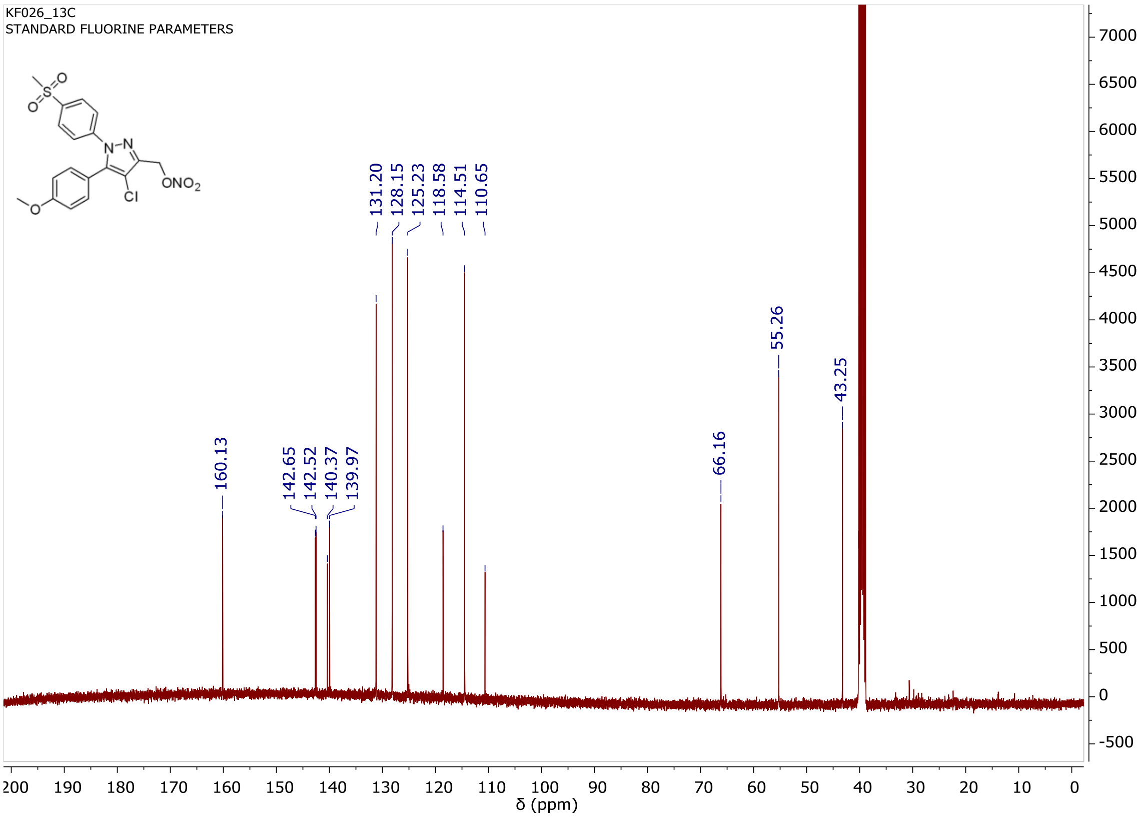

Figure S36. ^{13}C NMR spectrum of compound **5d** in DMSO-d_6

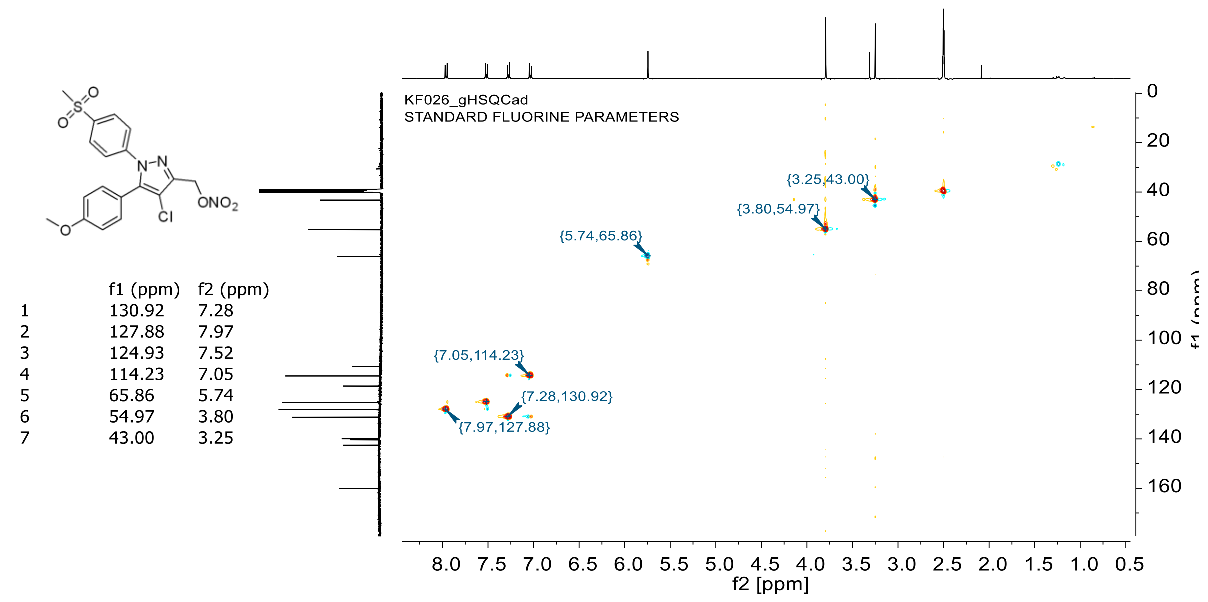

Figure S37. HSQC spectrum of compound **5d** in DMSO-d_6

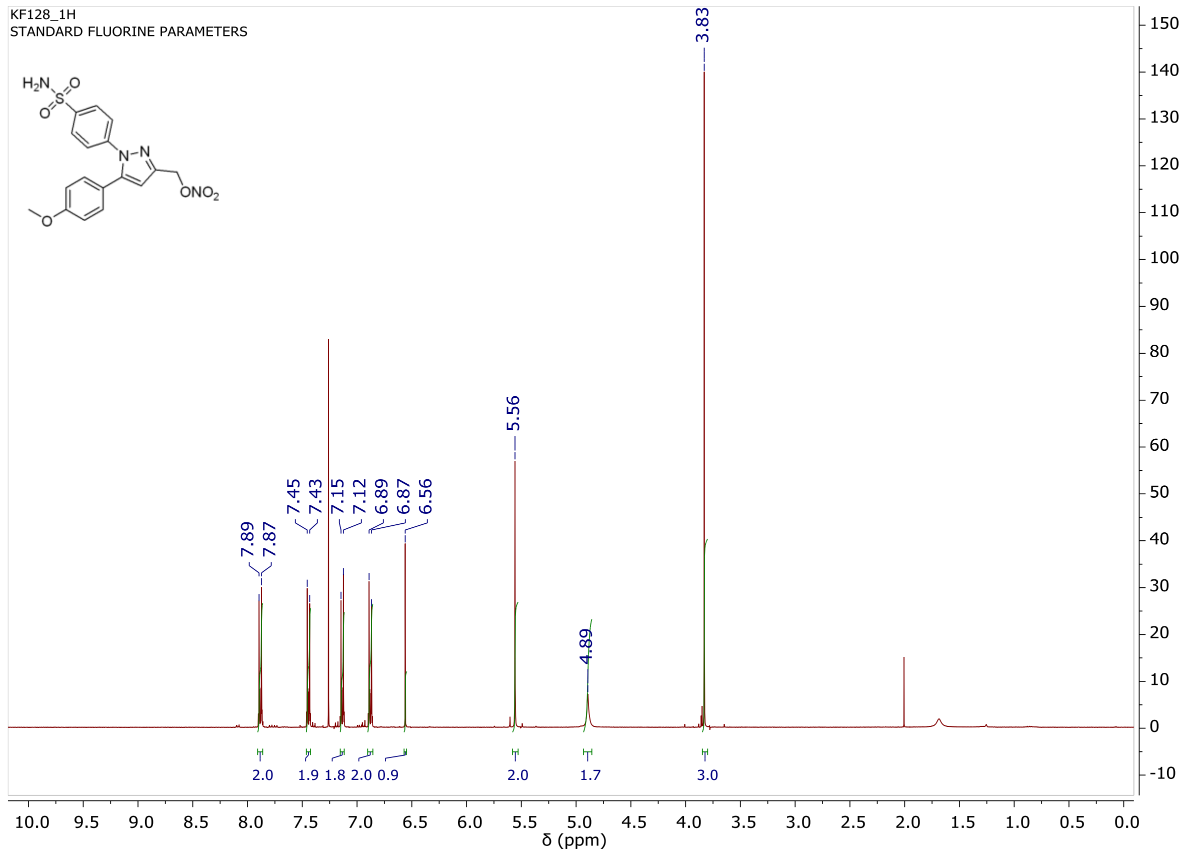

Figure S38. ^{1}H NMR spectrum of compound **5f** in $CDCl_3$

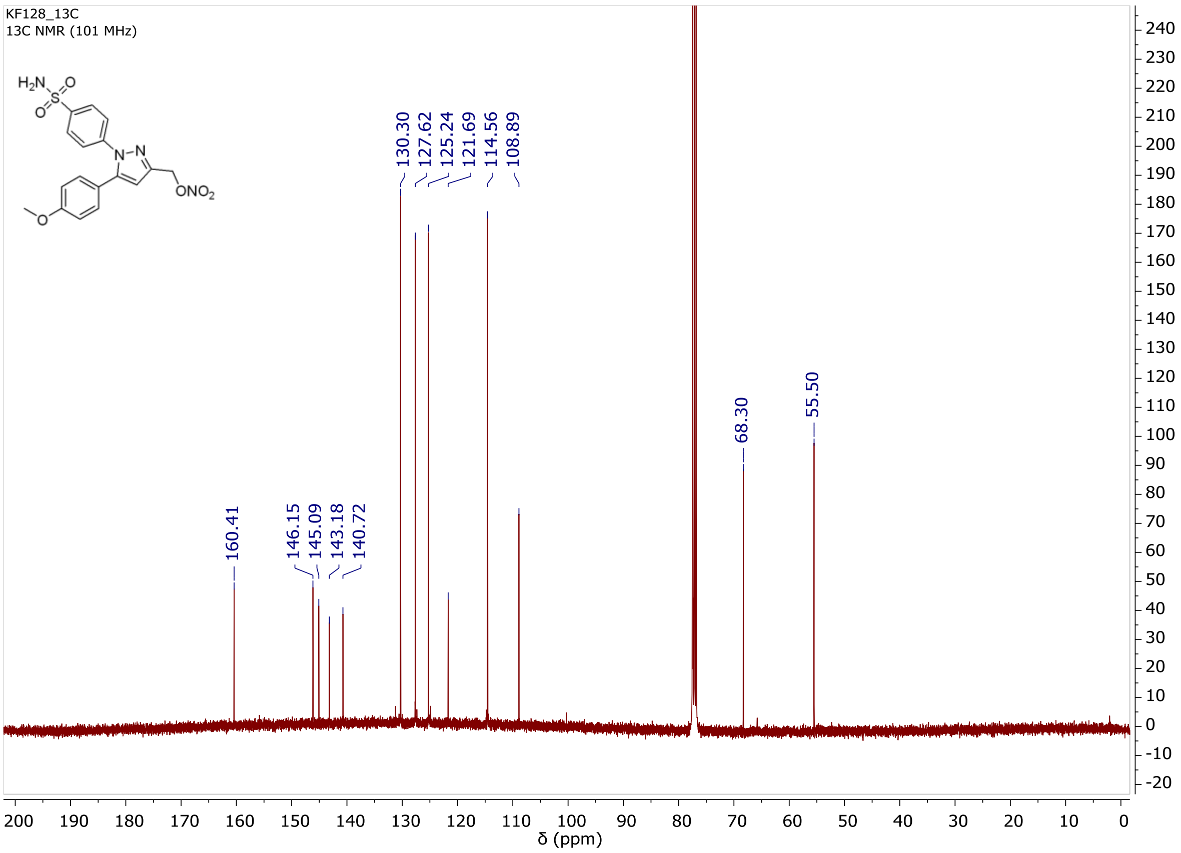

Figure S39. ^{13}C NMR spectrum of compound **5f** in $CDCl_3$

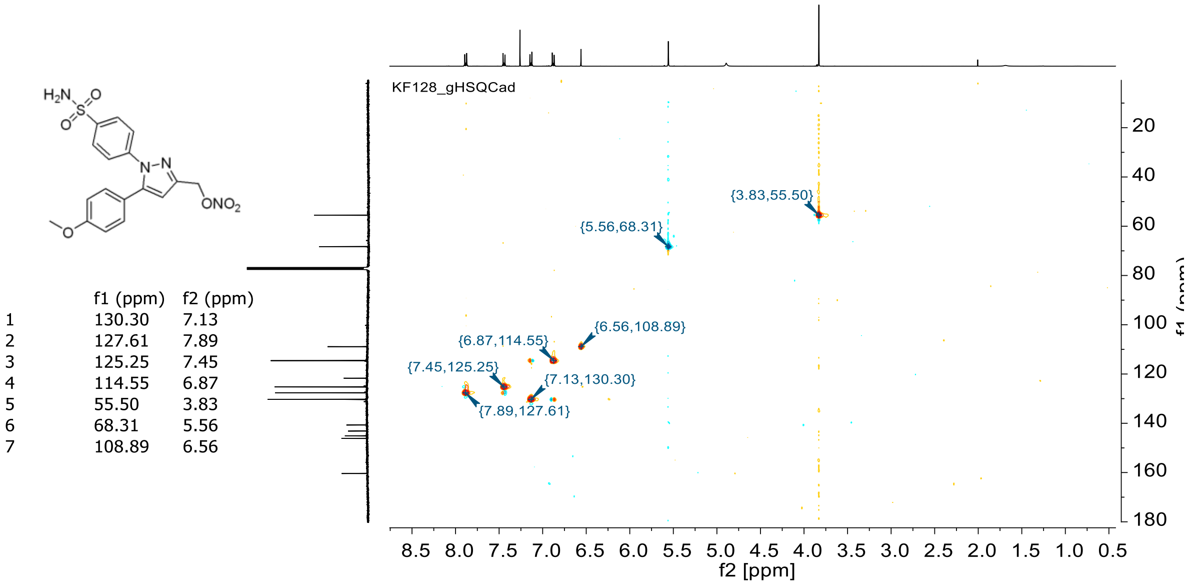

Figure S40. HSQC spectrum of compound **5f** in $CDCl_3$

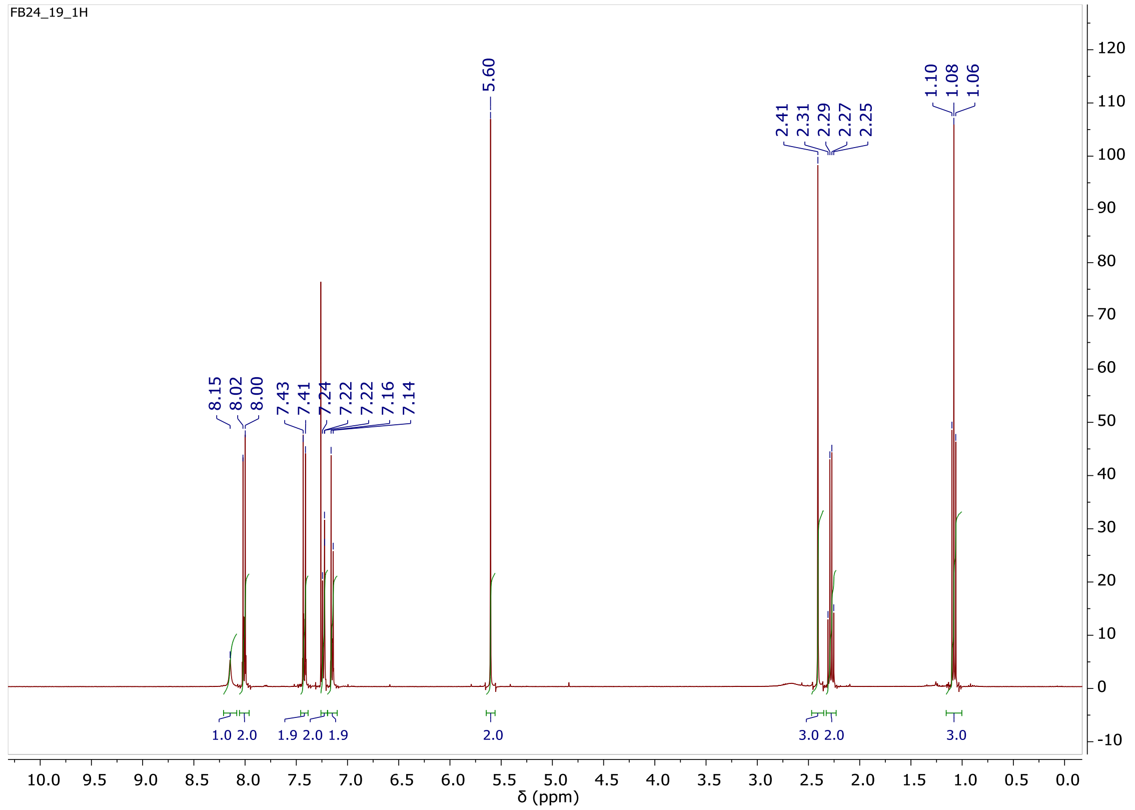

Figure S41. ^{1}H NMR spectrum of compound **6a** in $CDCl_3$

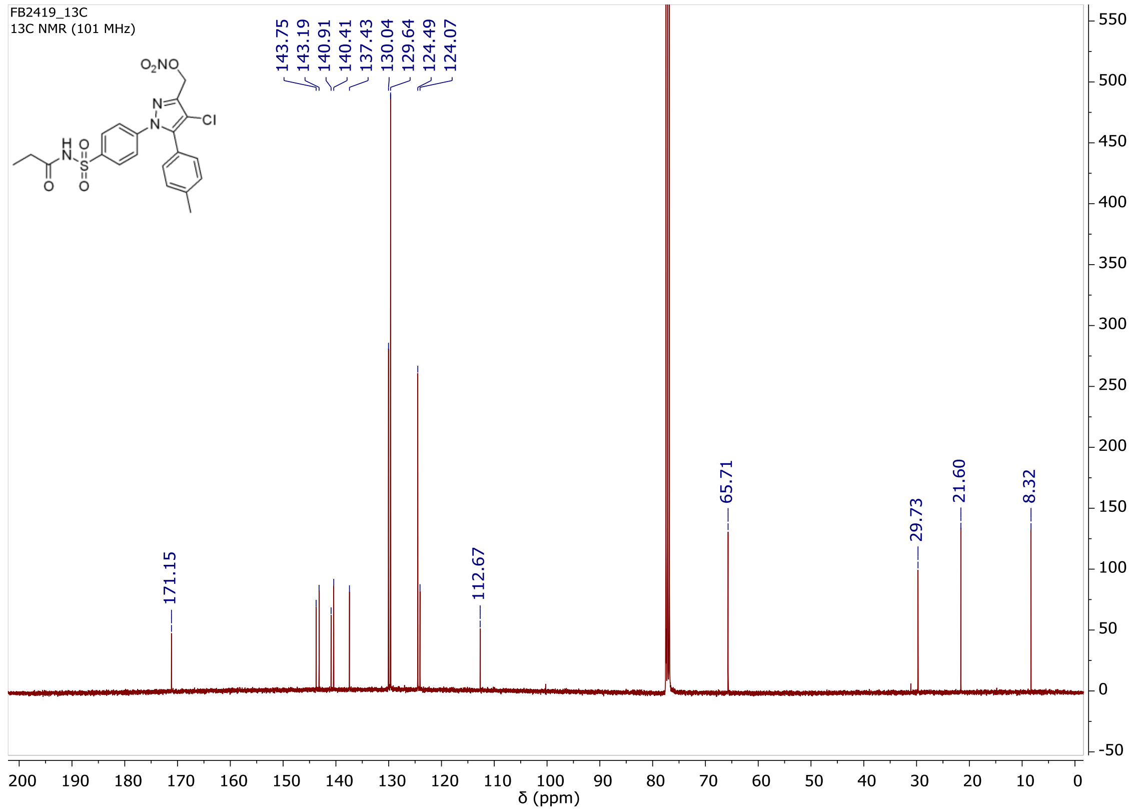

Figure S42. ^{13}C NMR spectrum of compound **6a** in $CDCl_3$

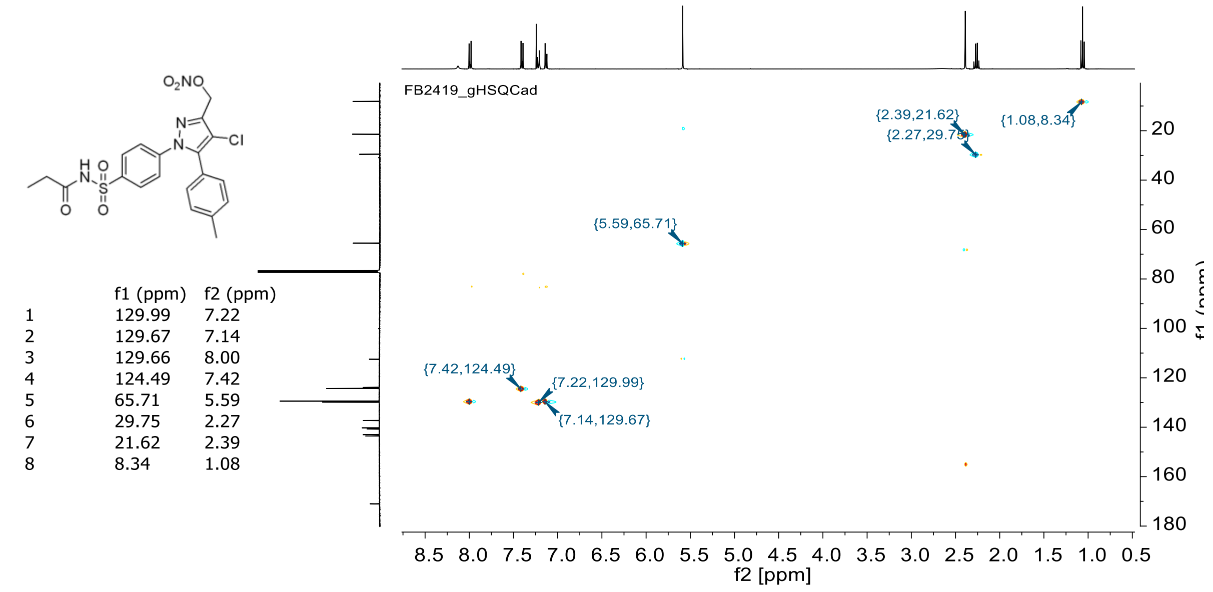

Figure S43. HSQC spectrum of compound **6a** in $CDCl_3$

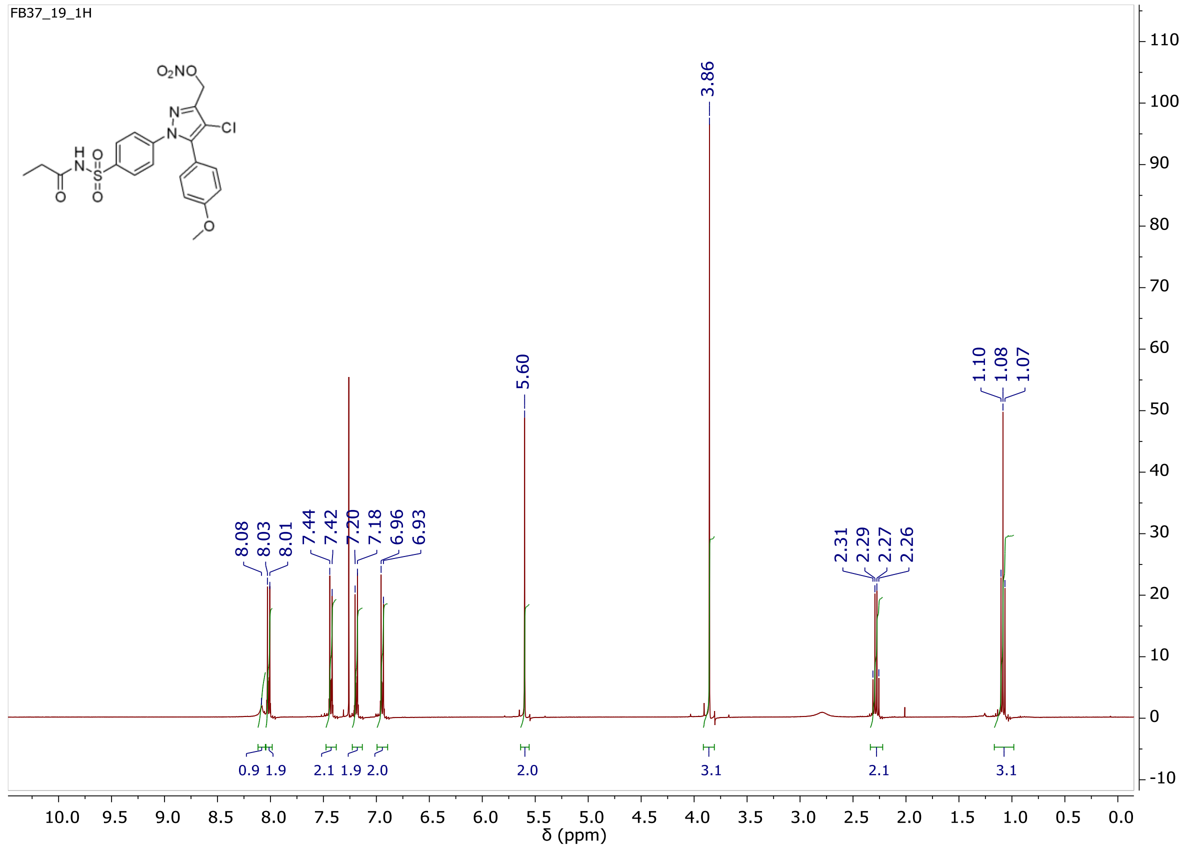

Figure S44. ^{1}H NMR spectrum of compound **6b** in $CDCl_3$

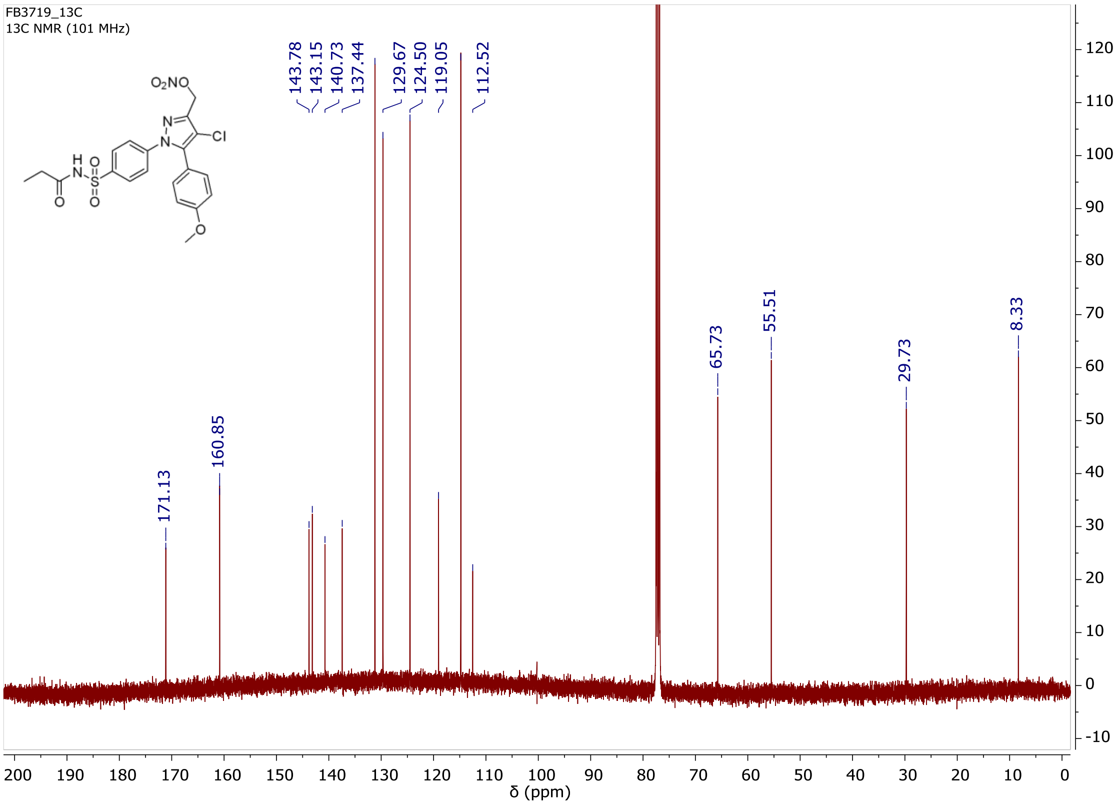

Figure S45. ^{13}C NMR spectrum of compound **6b** in $CDCl_3$

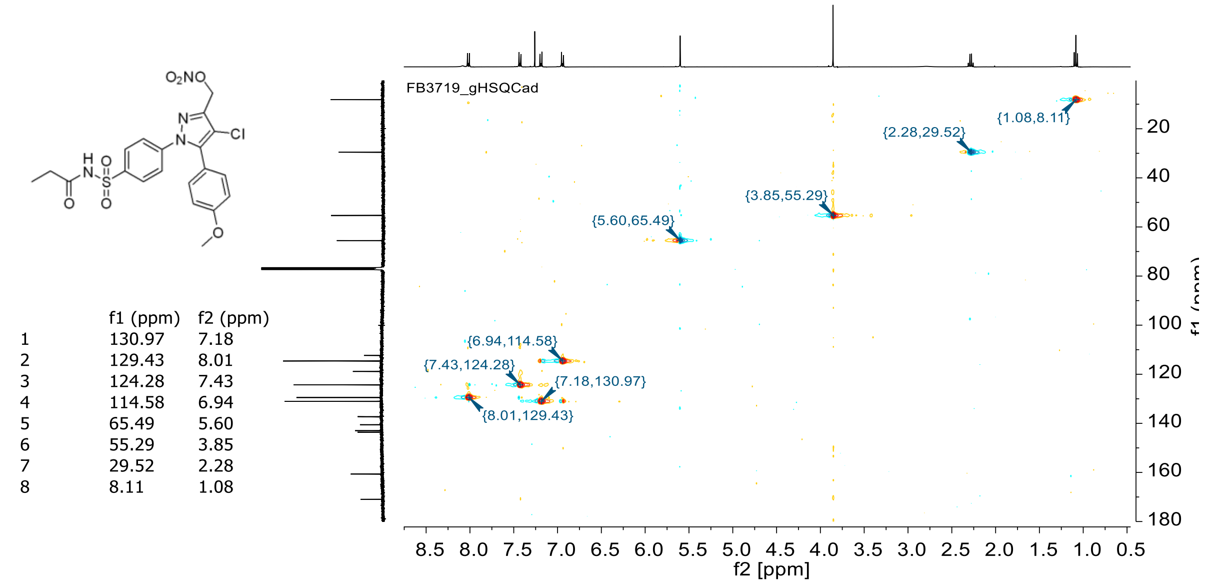

Figure S46. HSQC spectrum of compound **6b** in $CDCl_3$

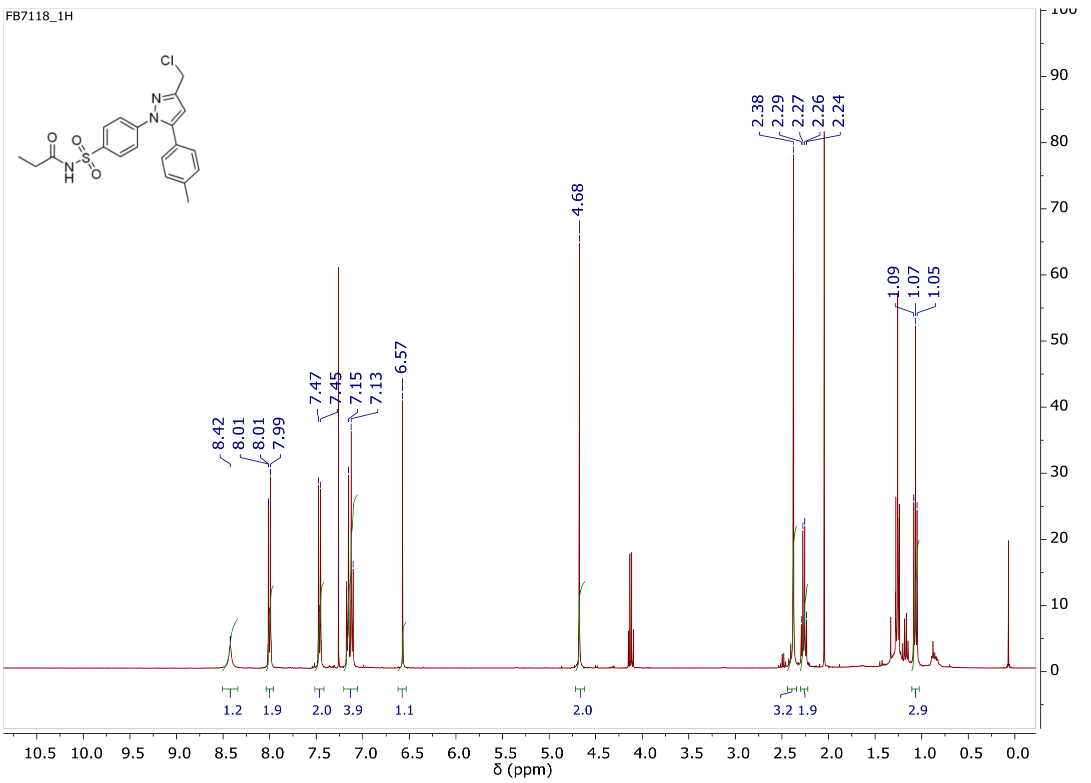

Figure S47. ^{1}H NMR spectrum of *N*-((4-(3-(chloromethyl)-5-(*p*-tolyl)-1*H*-pyrazol-1-yl)phenyl)-sulfonyl)propionamide in $CDCl_3$ obtained as intermediate product in the synthesis of compound **6c**

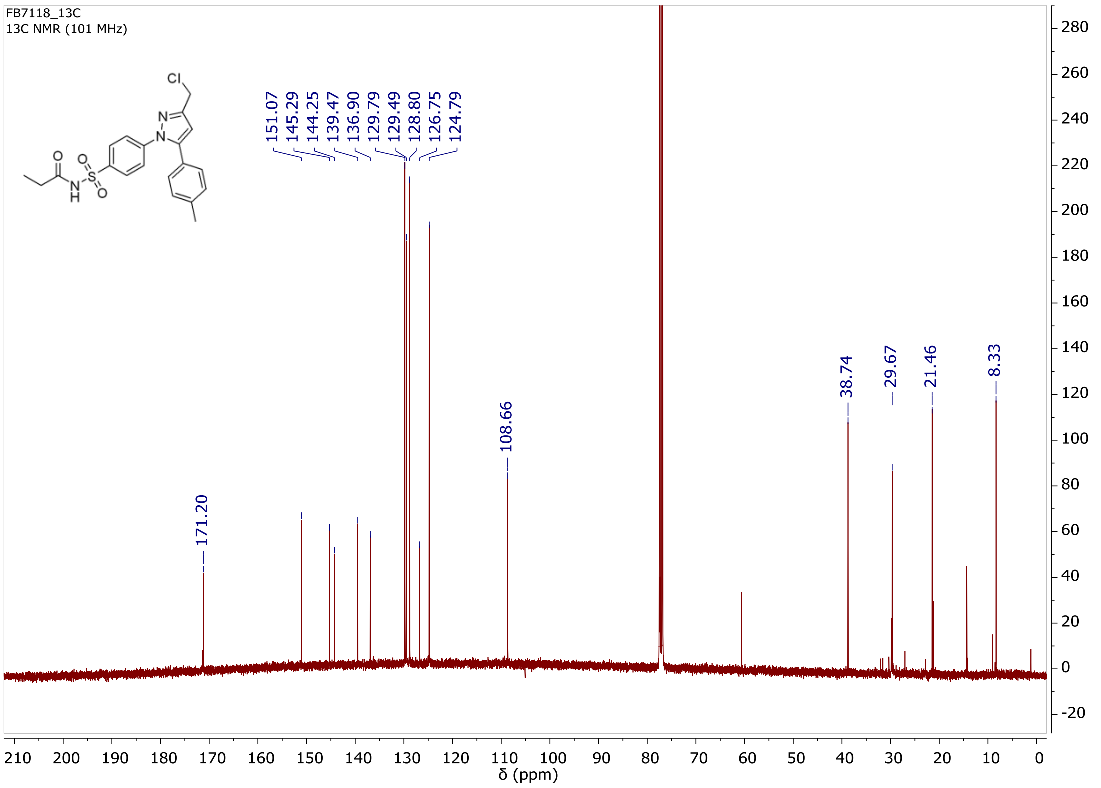

Figure S48. ^{13}C NMR spectrum of *N*-((4-(3-(chloromethyl)-5-(*p*-tolyl)-1*H*-pyrazol-1-yl)phenyl)-sulfonyl)propionamide in $CDCl_3$ obtained as intermediate product in the synthesis of compound **6c**

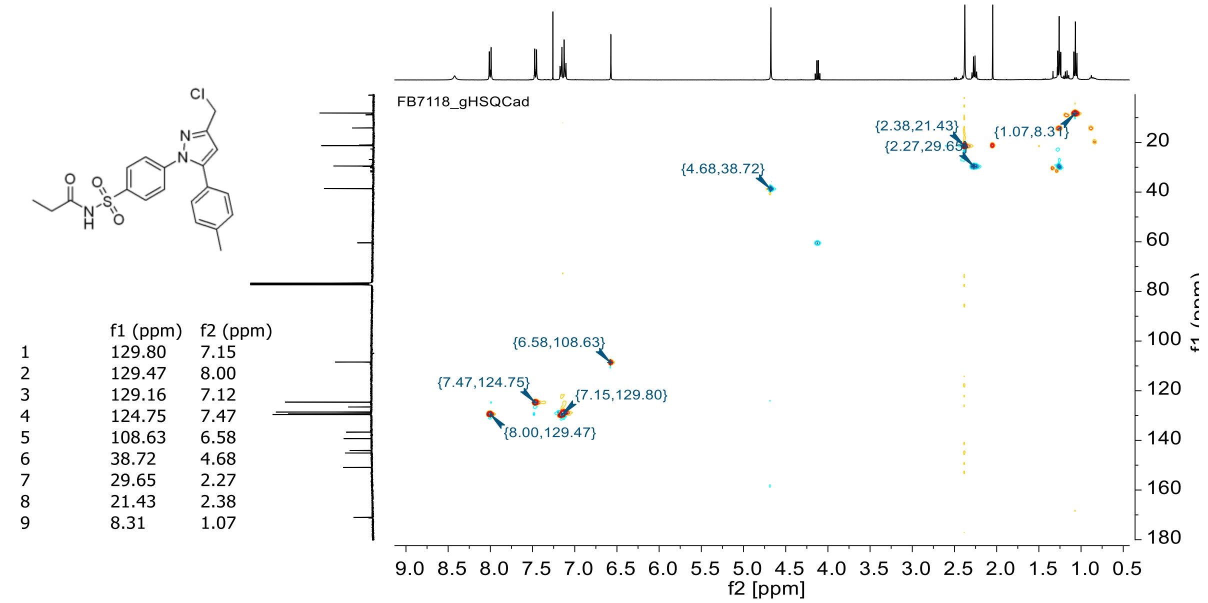

Figure S49. HSQC spectrum of *N*-((4-(3-(chloromethyl)-5-(*p*-tolyl)-1*H*-pyrazol-1-yl)phenyl)-sulfonyl)propionamide in $CDCl_3$ obtained as intermediate product in the synthesis of compound **6c**

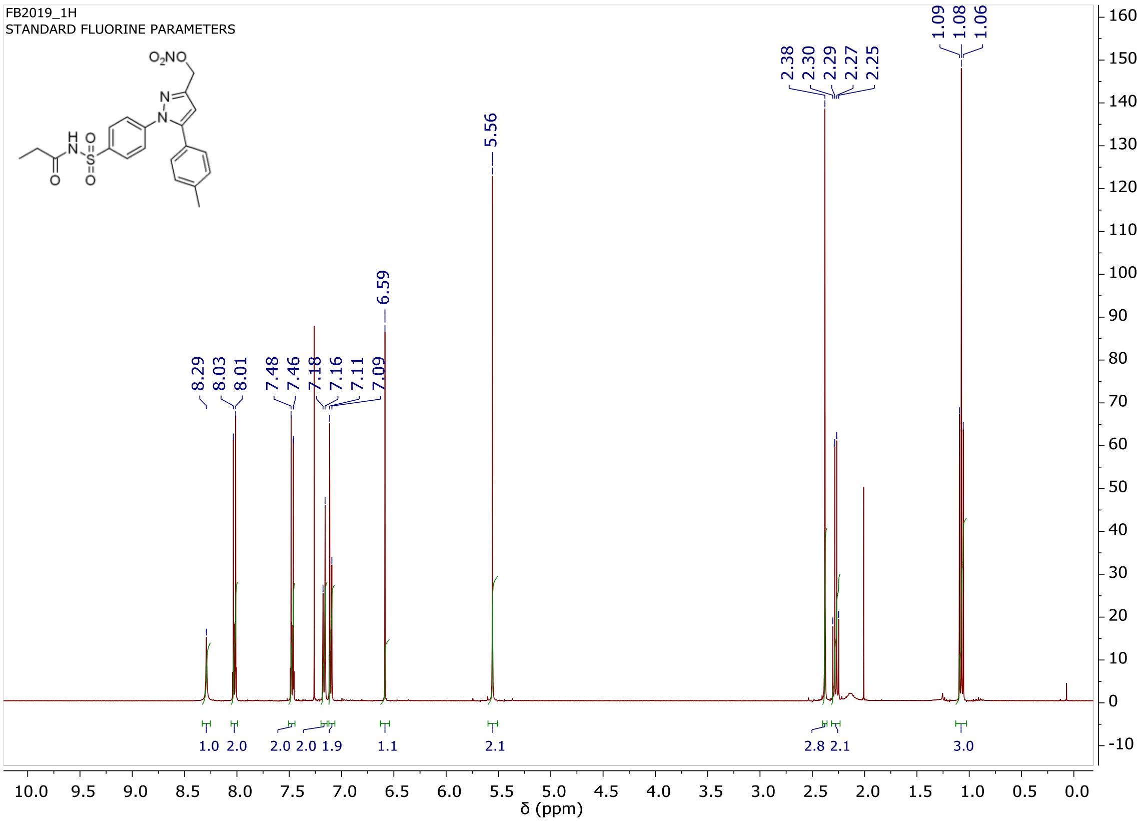

Figure S50. ^{1}H NMR spectrum of compound **6c** in $CDCl_3$

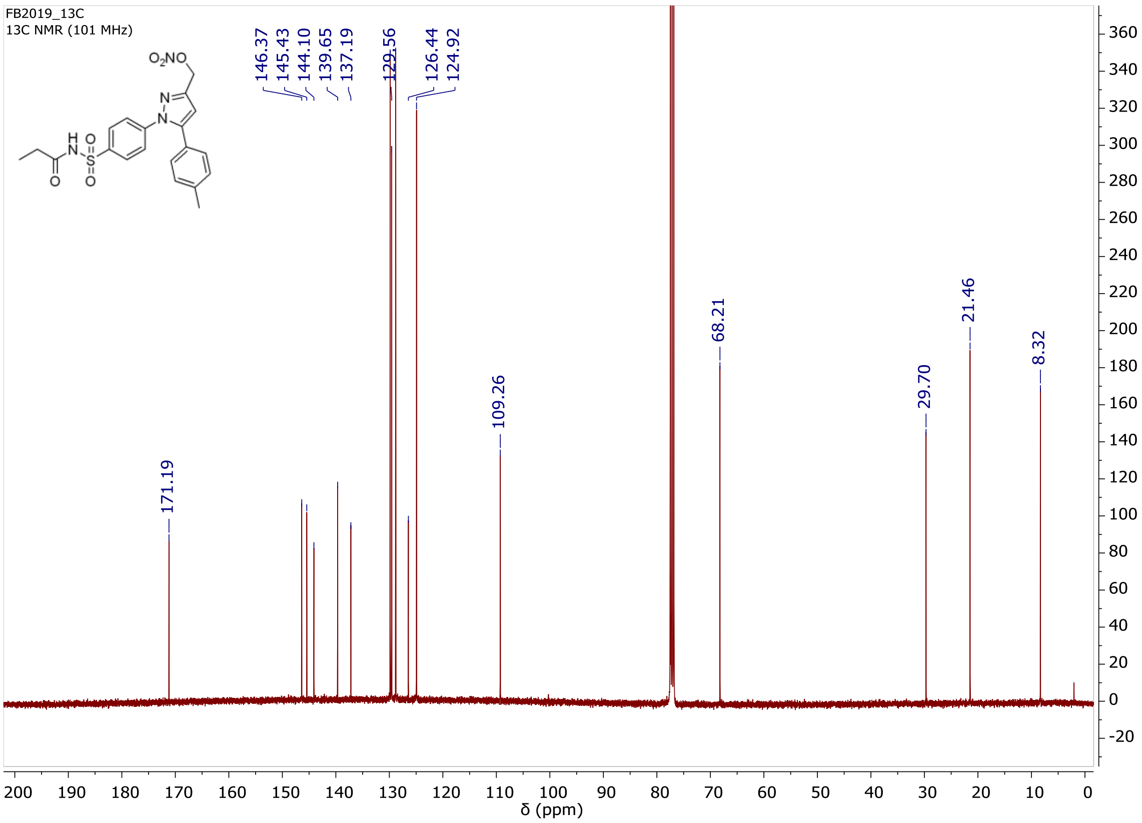

Figure S51. ^{13}C NMR spectrum of compound **6c** in $CDCl_3$

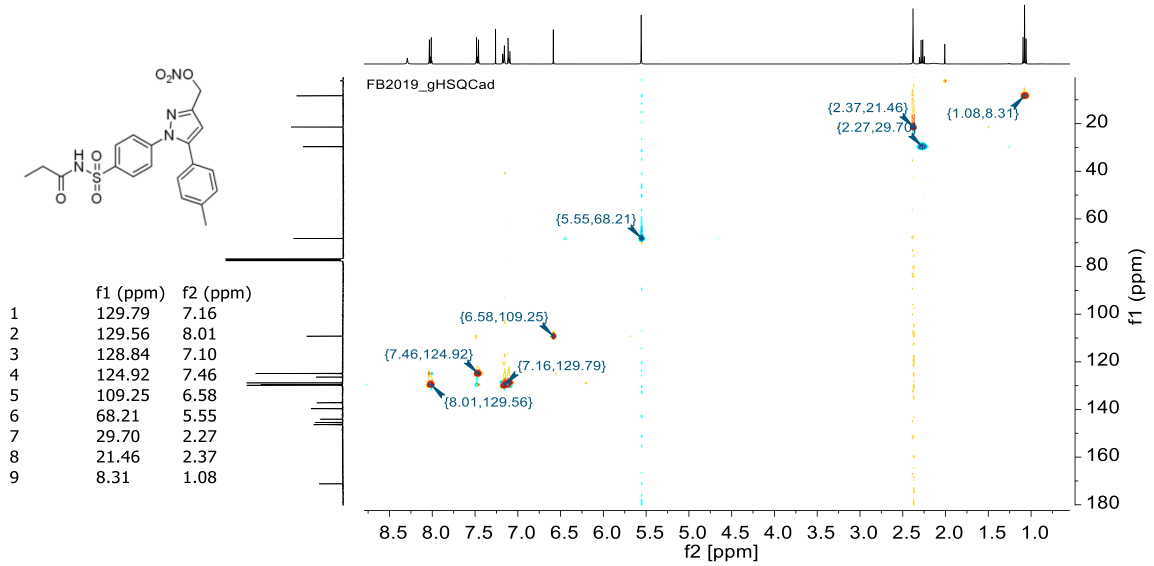

Figure S52. HSQC spectrum of compound **6c** in $CDCl_3$

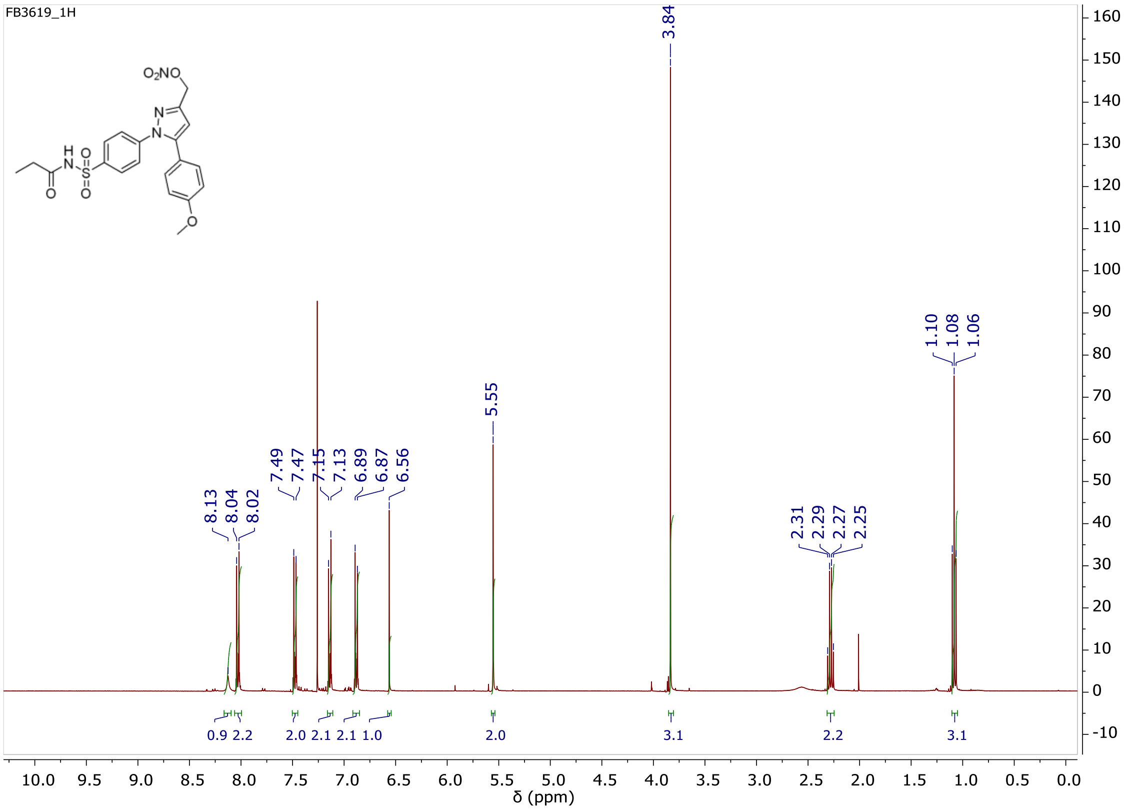

Figure S53. ^{1}H NMR spectrum of compound **6d** in $CDCl_3$

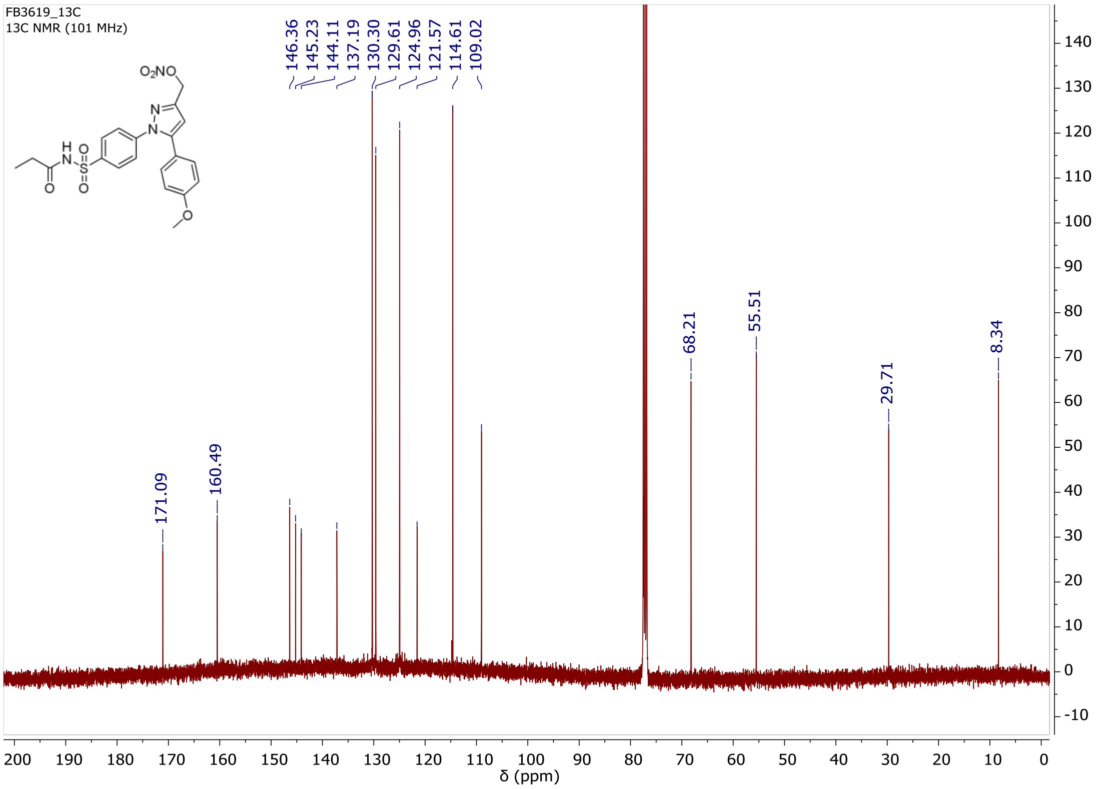

Figure S54. ^{13}C NMR spectrum of compound **6d** in $CDCl_3$

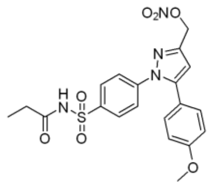

	f1 (ppm)	f2 (ppm)
1	130.27	7.14
2	129.61	8.03
3	124.95	7.48
4	114.58	6.88
5	109.00	6.57
6	68.18	5.55
7	55.49	3.84
8	29.71	2.28
9	8.32	1.08

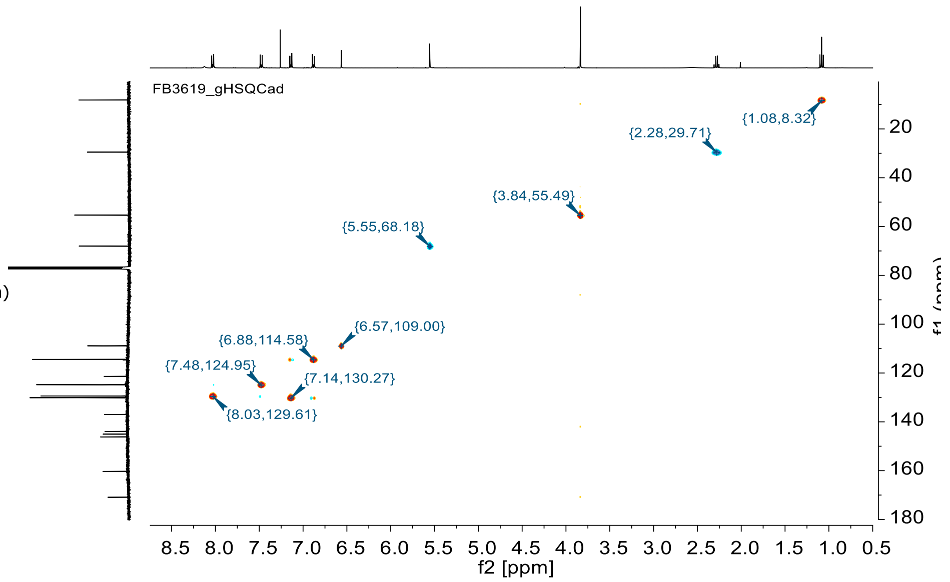

Figure S55. HSQC spectrum of compound **6d** in $CDCl_3$

2. Copy of HPLC-HRMS chromatograms after incubation in NO-assay buffer

Table S1. Overview on calculated and found HRMS data

	Exact Mass	M+H		M-NO2+2H		M+Cys-NO3	
		calculated	found	calculated	found	calculated	found
5a	422,0452	423,0530	423,0517	378,0680	378,0671	481,0771	481,0761
5b	438,0405	439,0483	439,0469	394,0636	394,0622	497,0724	497,0712
5c	421,0499	422,0577	422,057	377,0727	377,0719	480,0818	480,0809
5d	437,0448	438,0526	438,0519	393,0676	393,0668	496,0767	496,0758
5e	388,0841	389,0919	389,0913	344,1069	344,1058	447,1160	447,1147
5f	404,0791	405,0869	405,0864	360,1019	360,1012	463,1110	463,1099
6a	478,0714	479,0792	479,0783	434,0942	434,0936	537,1033	537,1027
6b	494,0663	495,0741	495,0731	450,0892	450,0885	553,0982	553,0972
6c	444,1104	445,1182	445,1175	400,1332	400,1327	503,1423	503,1412
6d	460,1053	461,1131	461,1115	416,1281	416,1269	519,1372	519,1359

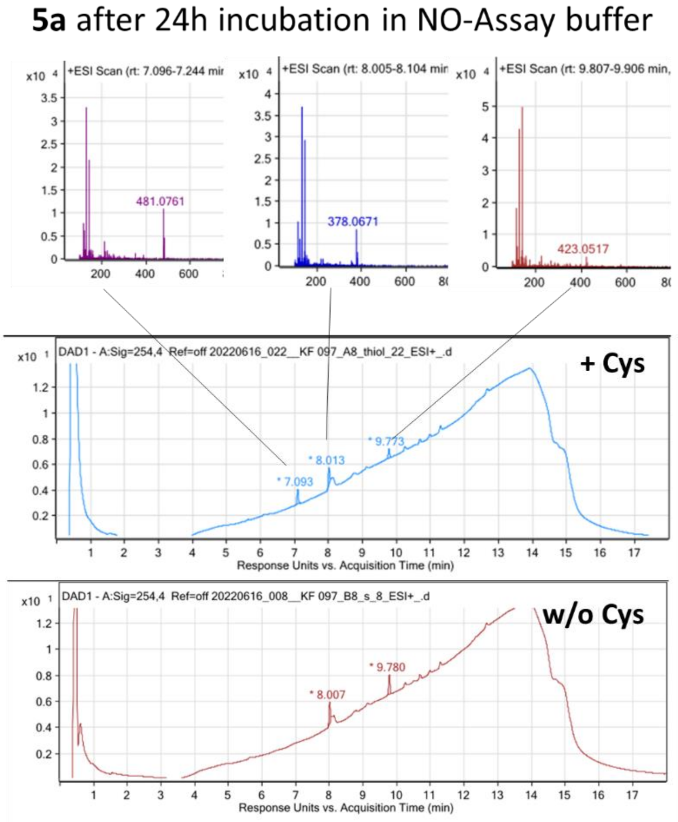

Figure S56. ***HPLC-DAD/HRMS data from 24h incubation of 5a in NO-Assay buffer***

5b after 24h incubation in NO-Assay buffer

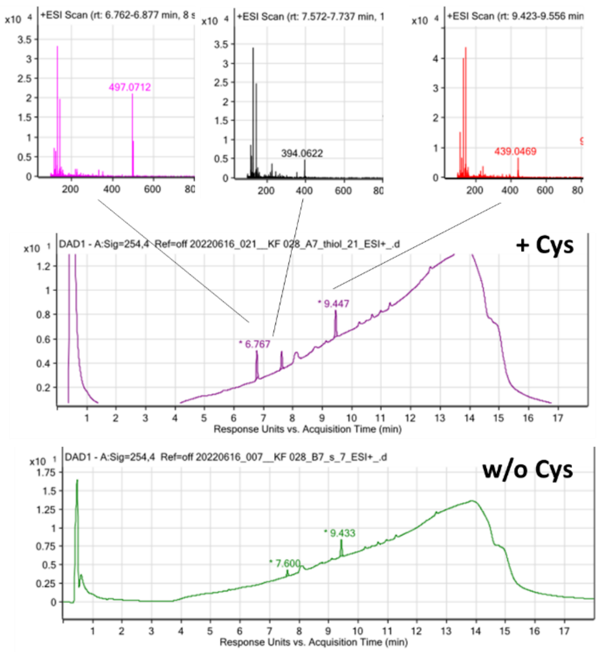

Figure S57. *HPLC- DAD/HRMS data from 24h incubation of **5b** in NO-Assay buffer*

5c after 24h incubation in NO-Assay buffer

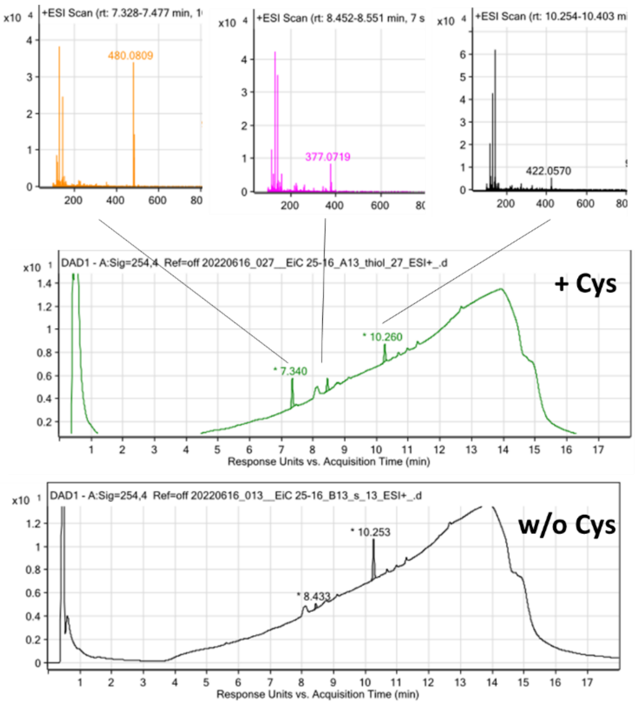

Figure S58. *HPLC-DAD/HRMS data from 24h incubation of **5c** in NO-Assay buffer*

5d after 24h incubation in NO-Assay buffer

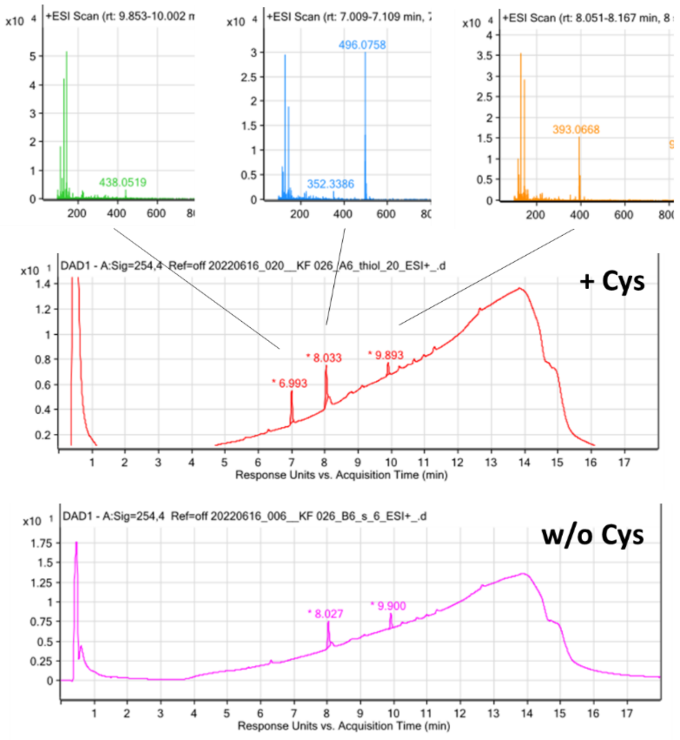

Figure S59. *HPLC-DAD/HRMS data from 24h incubation of **5d** in NO-Assay buffer*

5e after 24h incubation in NO-Assay buffer

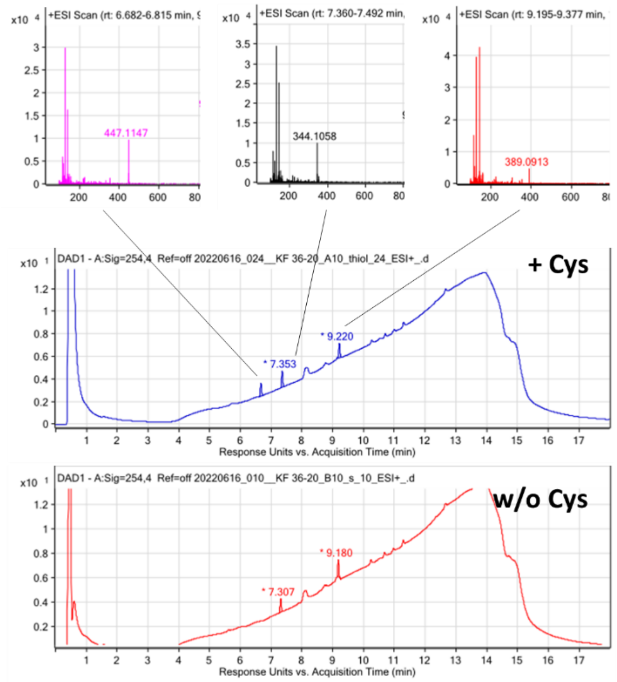

Figure S60. *HPLC-DAD/HRMS data from 24h incubation of **5e** in NO-Assay buffer*

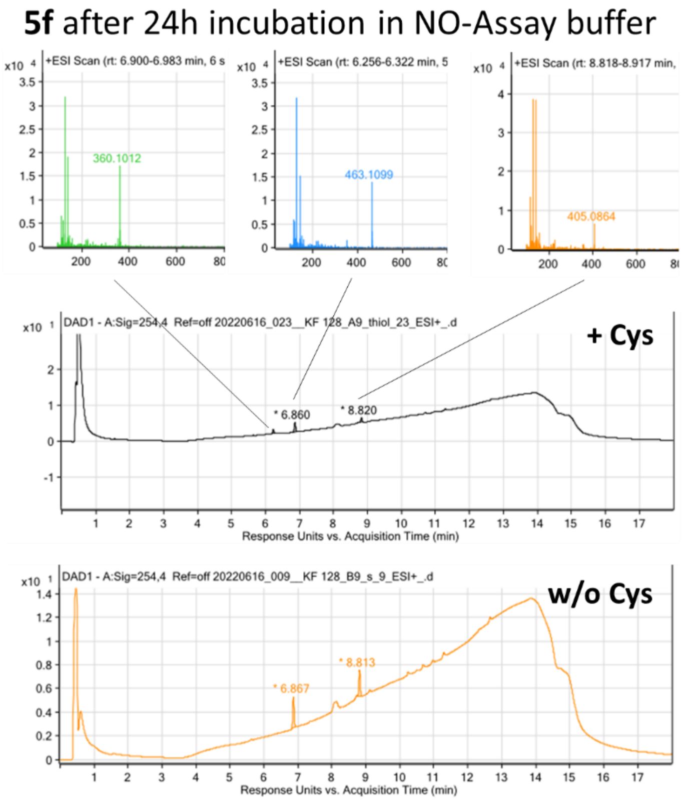

Figure S61. *HPLC-DAD/HRMS data from 24h incubation of **5f** in NO-Assay buffer*

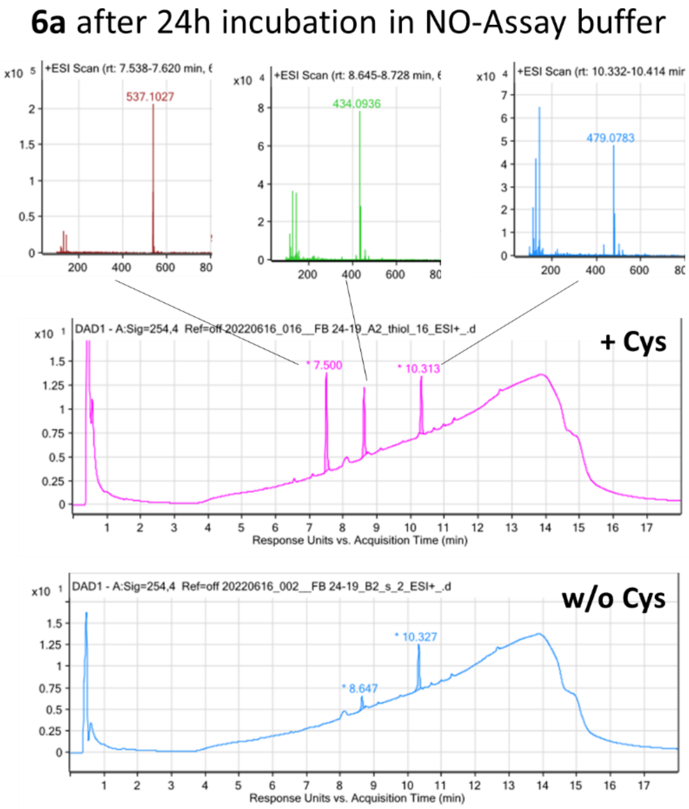

Figure S62. *HPLC-DAD/HRMS data from 24h incubation of **6a** in NO-Assay buffer*

6b after 24h incubation in NO-Assay buffer

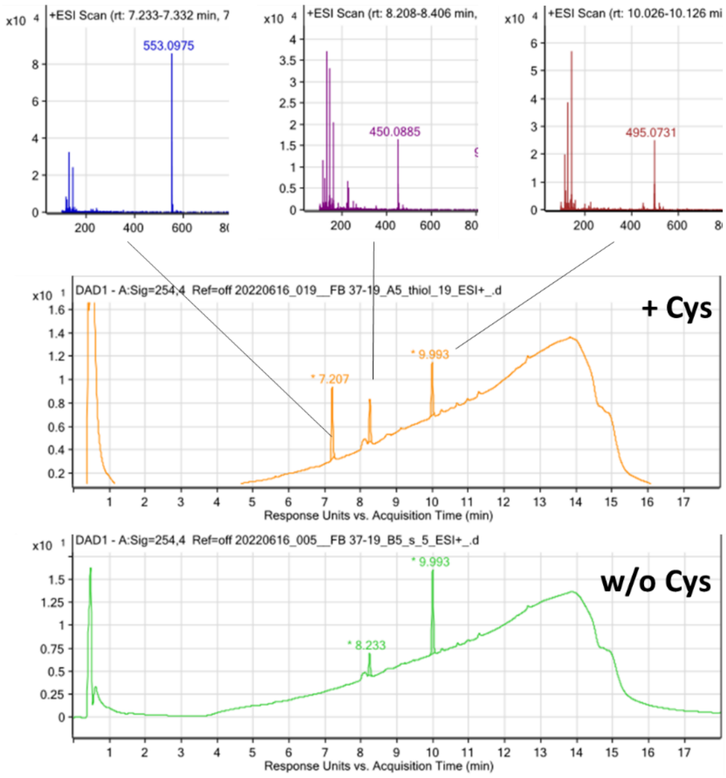

Figure S63. *HPLC-DAD/HRMS data from 24h incubation of **6b** in NO-Assay buffer*

6c after 24h incubation in NO-Assay buffer

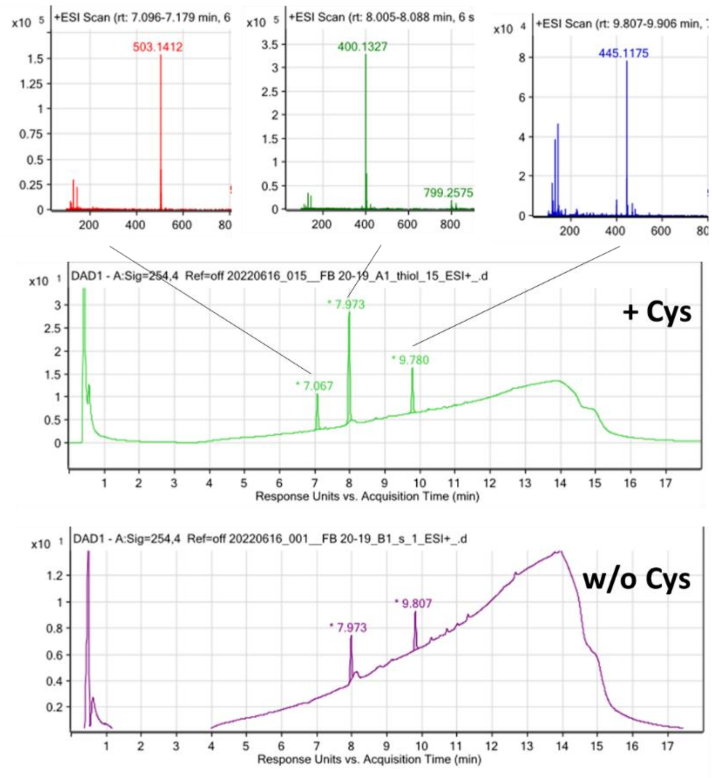

Figure S64. *HPLC-DAD/HRMS data from 24h incubation of **6c** in NO-Assay buffer*

6d after 24h incubation in NO-Assay buffer

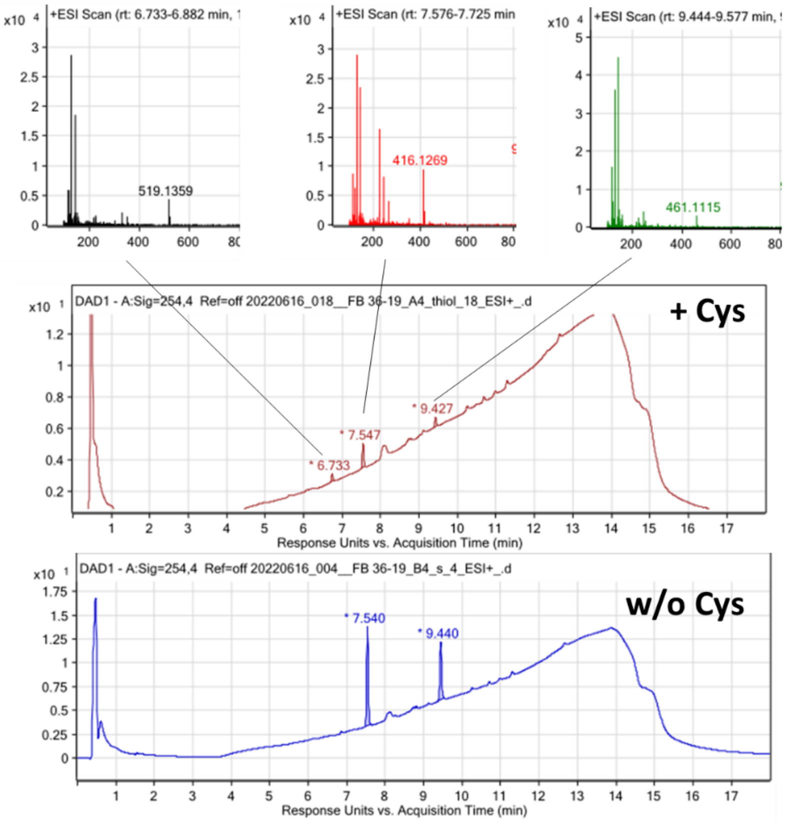

Figure S65. *HPLC-DAD/HRMS data from 24h incubation of **6d** in NO-Assay buffer*

Blank 24h incubation in NO-Assay buffer

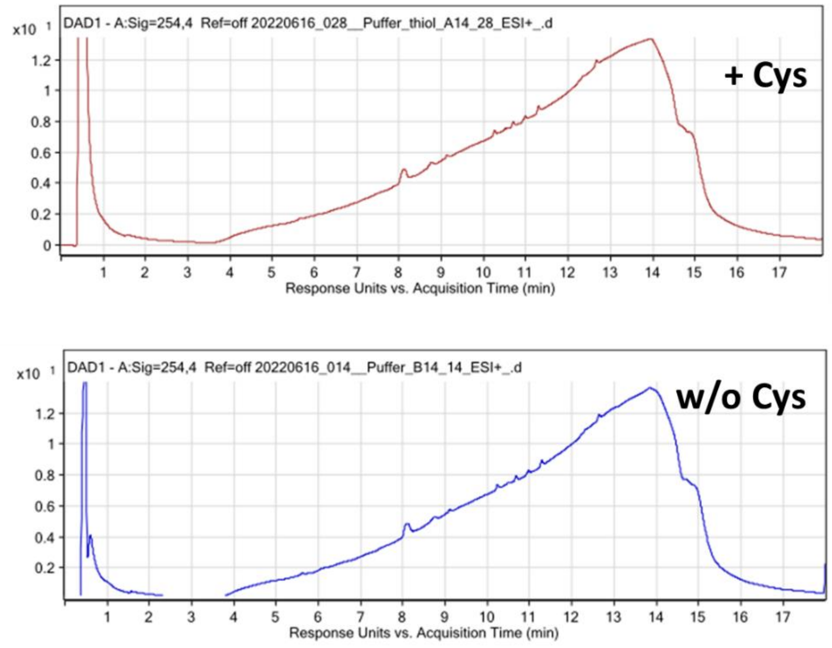

Figure S66. *HPLC-DAD/HRMS data from 24h incubation without inhibitor in NO-Assay buffer*

3. Expression levels of *Ptgs1* and *Ptgs2* genes in genetically modified MPC cell lines

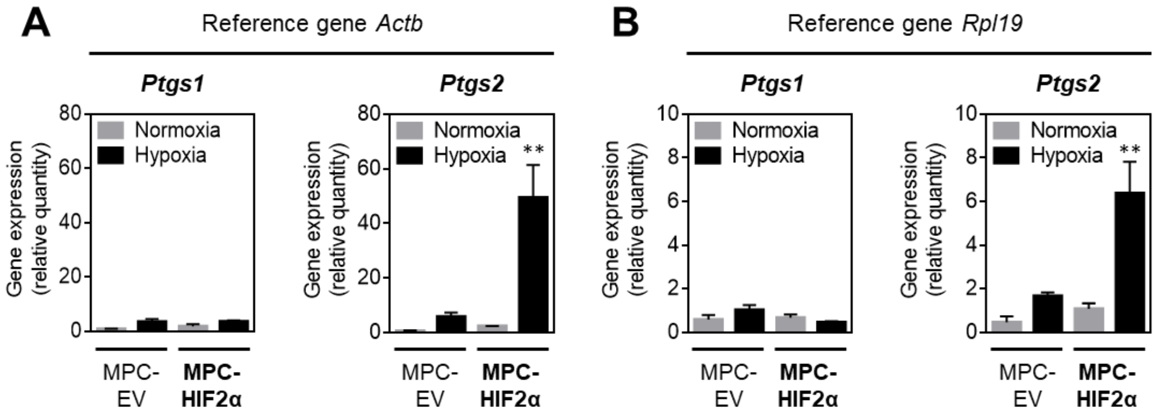

Figure S67: Expression levels of *Ptgs1* and *Ptgs2* genes encoding COX-1 and COX-2, respectively, in genetically modified MPC cell lines; *n* = 3; relative gene expression measured relative to the reference genes *Actb* (A) and *Rpl19* (B); all ΔΔCt values were normalized to MPC-EV sample 1; (EV) 'empty vector'; (HIF2α) expression vector containing a codon-optimized *Epas1* gene encoding HIF2α; Hypoxia: cells were cultured for 24h at 1% oxygen in the gas phase; significance of differences compared to MPC-EV, Normoxia: ** $P < 0.01$

4. Growth-rate of tumor spheroids in the presence of NO-COXIBS without radiation treatment

Compound	*Diameter growth rate [µm/day] ± SEM*
w/o	58.6 ± 4.3
celecoxib	57.3 ± 4.0
5a	54.1 ± 5.2
5c	60.4 ± 5.3
5d	61.2 ± 5.5
5f	61.4 ± 5.2
6a	64.1 ± 6.1
6b	63.1 ± 5.3
6c	60.0 ± 5.5
6d	63.7 ± 5.9

Table S2. **Effects of NO-COXIBs on spheroid growth;** diameter growth rates of non-irradiated MPC-HIF2a spheroids in presence of 10 µM of compounds determined from linear regression between 0 and 9 days after treatment start; (w/o) vehicle control treated with DMSO

5. Detailed growth response of tumor spheroids in the presence of test compounds

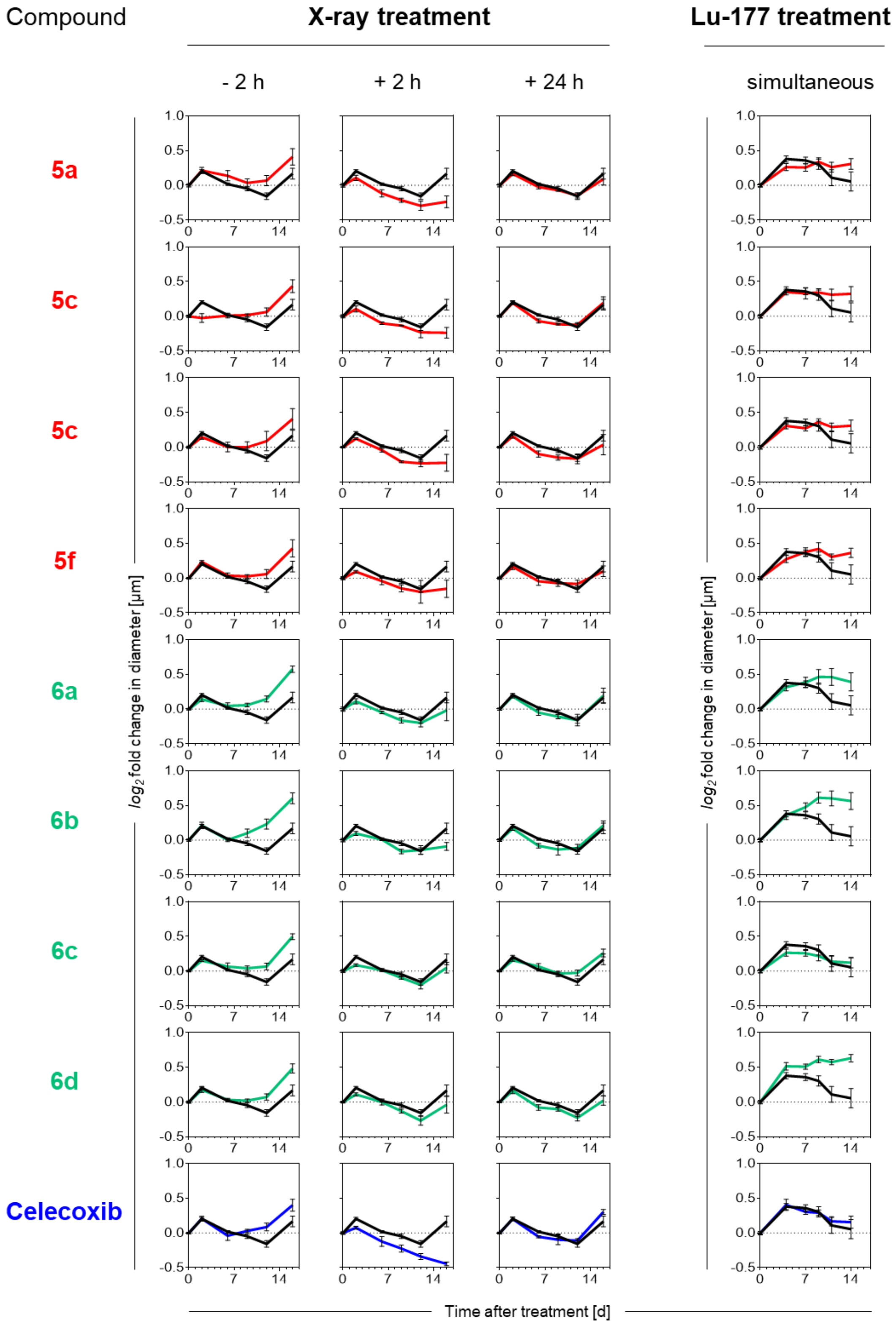

Figure S68. Effects of NO-COXIBs on radiation treatments of MPC-HIF2α tumor spheroids; (A) Growth responses to X-ray treatment with a radiation dose of 15 Gy in combination with 10 µM NO-COXIBs added to the medium at indicated time points before or after irradiation as well as to

[^{177}Lu]$LuCl_3$ treatment with an initial activity concentration of 0.25 MBq/mL in combination with NO-COXIBs; *log₂* fold changes in diameter indicate the number of diameter doublings compared to treatment start. Black lines indicate growth response in the vehicle (w/o) treated control. (Ce) celecoxib.
